# Cellular heterogeneity contributes to subtype-specific expression of ZEB1 in human glioblastoma

**DOI:** 10.1371/journal.pone.0185376

**Published:** 2017-09-25

**Authors:** Philipp Euskirchen, Josefine Radke, Marc Sören Schmidt, Eva Schulze Heuling, Eric Kadikowski, Meron Maricos, Felix Knab, Ulrike Grittner, Norman Zerbe, Marcus Czabanka, Christoph Dieterich, Hrvoje Miletic, Sverre Mørk, Arend Koch, Matthias Endres, Christoph Harms

**Affiliations:** 1 Dept. of Neurology, Charité –Universitätsmedizin Berlin, Berlin, Germany; 2 Dept. of Experimental Neurology, Charité –Universitätsmedizin Berlin, Berlin, Germany; 3 Berlin Institute of Health (BIH), Berlin, Germany; 4 Dept. of Neuropathology, Charité –Universitätsmedizin Berlin, Berlin, Germany; 5 German Cancer Consortium (DKTK), Partner Site Charité Berlin, Berlin, Berlin, Germany; 6 Center for Stroke Research Berlin, Charité –Universitätsmedizin Berlin, Berlin, Germany; 7 Dept. for Biostatistics and Clinical Epidemiology, Charité –Universitätsmedizin Berlin, Berlin, Germany; 8 Dept. of Pathology, Charité –Universitätsmedizin Berlin, Berlin, Germany; 9 Dept. of Neurosurgery, Charité –Universitätsmedizin Berlin, Berlin, Germany; 10 Computational RNA Biology and Ageing Group, Max-Planck-Institute for the Biology of Ageing, Cologne, Germany; 11 Dept. of Biomedicine, University of Bergen, Bergen, Norway; 12 Department of Pathology, Haukeland University Hospital, Bergen, Norway; 13 Deutsches Zentrum für Herz-Kreislauf-Forschung (DZHK), Standort Berlin, Berlin, Germany; 14 Deutsches Zentrum für Neurodegenerative Erkrankungen (DZNE), Standort Berlin, Berlin, Germany; Swedish Neuroscience Institute, UNITED STATES

## Abstract

The transcription factor ZEB1 has gained attention in tumor biology of epithelial cancers because of its function in epithelial-mesenchymal transition, DNA repair, stem cell biology and tumor-induced immunosuppression, but its role in gliomas with respect to invasion and prognostic value is controversial. We characterized ZEB1 expression at single cell level in 266 primary brain tumors and present a comprehensive dataset of high grade gliomas with Ki67, p53, IDH1, and EGFR immunohistochemistry, as well as EGFR FISH. ZEB1 protein expression in glioma stem cell lines was compared to their parental tumors with respect to gene expression subtypes based on RNA-seq transcriptomic profiles. ZEB1 is widely expressed in glial tumors, but in a highly variable fraction of cells. In glioblastoma, ZEB1 labeling index is higher in tumors with EGFR amplification or IDH1 mutation. Co-labeling studies showed that tumor cells and reactive astroglia, but not immune cells contribute to the ZEB1 positive population. In contrast, glioma cell lines constitutively express ZEB1 irrespective of gene expression subtype. In conclusion, our data indicate that immune infiltration likely contributes to differential labelling of ZEB1 and confounds interpretation of bulk ZEB1 expression data.

## Introduction

Glioblastoma (GBM) is the most frequent primary brain tumor in adults and characterized by rapid, highly infiltrative growth and early recurrence after treatment. Despite intensive research and multimodal treatment including surgical resection, radiotherapy and chemotherapy, patients today still face a dismal prognosis with a median survival of 15 months [1].

In tumors of epithelial origin, epithelial-mesenchymal transition (EMT), i.e. phenotypic plasticity towards an invasive phenotype, is considered an important mechanism in mediating cell motility necessary for invasion and metastasis. EMT is observed physiologically (e.g. during embryonic development) and under pathological conditions (e.g. in carcinomas). At the molecular level, this process is induced by key transcription factors such as ZEB1 as well as *twist* and *snail* homologs [2]. Mechanistically, all EMT inducers regulate cadherins, integrins and extracellular matrix proteins, which may be used as molecular markers of EMT. Additionally, ZEB1 has been reported to confer stemness properties to cancer cells by inducing expression of Sox2 and Bmi1 transcription factors via suppression of miR-200 family microRNAs [3]. In addition to this canonical EMT signaling, ZEB1 has lately been shown to be involved in DNA damage response [4] and modulation of tumor induced immunosuppression [5]. For epithelial cancers, this has led to a model of tumor progression in which ZEB1 serves as a molecular marker. ZEB1 and EMT have intensively been studied in carcinomas and has sparked several investigations of ZEB1 in primary brain tumors where it has been proposed to mediate response to hypoxia and increase proliferation, cell migration and invasion [6–9]. Recent experimental data conflictingly propose either a specialized role for ZEB1 in the invasive egde [9] or as universal effector of oncogenic signaling from various driver events [10].

We here provide a comprehensive characterization of ZEB1 in a large series of human glioblastoma with respect to clinical and molecular traits. Our results suggest a constitutive role for ZEB1 in astroglial tumors and identify immune infiltration as a contributor to ZEB1 intratumoral heterogeneity.

## Materials and methods

### Patient samples

A tissue microarray (TMA) of 243 human high grade glioma samples has been described before [11]. Briefly, patient samples diagnosed with glioblastoma and WHO grade III glioma were collected from 1998 to 2008. Three tissue cores per tumor were included in the TMA. Mean patient age at primary diagnosis of GBM was 63.1 ± 10.9 years and median survival of GBM cases was 9.1 months.

The generation of the primary glioma cell lines from patient material used in this study was approved by the local ethics committee (Charité –Universitätsmedizin Berlin, EA1/265/12) with informed consent given by all study participants and has been described before [12]. Briefly, fresh surgical samples were dissociation mechanically and enzymatically, washed and subjected to erythrocyte lysis before culture under neurosphere conditions.

### Cell culture

Cells were grown in Neurobasal medium (Life technologies) supplemented with 0.5X N2 supplement (Life Technologies) and 0.5X B27 (Life Technologies) as described [13], but with 20 ng ml^−1^ EGF (Peprotech), 20 ng ml^−1^ bFGF (Peprotech). Spheroids were dissociated using Accutase (Life Technologies). For adherent cell culture, 8-well chamber slides (Ibidi, Martinsried, Germany) were coated with 10 μg/ml laminin (Sigma, cat.no. L2020) for three hours or overnight [14]. Wells were washed three times with PBS and stored at 4°C until use.

### Immunohistochemistry

Immunohistochemical staining of tissue microarray and full biopsy 4 μm thick FFPE tissue sections was performed on a VENTANA Benchmark XT automated staining instrument according to the manufacturer's instructions. Slides were de-paraffinized using EZ prep solution (Ventana Medical Systems, Tucson, AZ) for 30 minutes at 75°C. Antigen retrieval was accomplished on the automated stainer using CC1 solution (Ventana Medical Systems, Tucson, AZ) for 60 minutes at 95°C. The anti-ZEB1 primary antibody (HPA027524, Sigma-Aldrich, dilution 1:300, characterized as part of the Human Protein Atlas [15]) was applied and developed using the iVIEW DAB Detection Kit (Ventana Medical Systems, Tucson, AZ). All slides were then counterstained with hematoxylin for 4 minutes.

For EGFR immunohistochemistry, slides were de-paraffinized using Xylol and antigen retrieval was performed by incubation at room temperature with Proteinase K (DAKO, S302080-2) for 7 minutes. Slides were then blocked for 20 minutes with 5% BSA in TBS-T and incubated with the EGFR primary antibody (Santa Cruz, sc-120, 1:500) for one hour at room temperature and then overnight at 4°C. After treating slides with 3% hydrogen peroxide for 5 minutes, incubation with secondary antibody (1:100) for one hour at room temperature followed. Finally, slides were covered with ABC-solution for 30 minutes, DAB chromogen for two minutes and then counterstained with hematoxylin for 90 seconds.

Slides were scanned using the Pannoramic 250 Flash scanner (3DHistech). Digitalization was performed with extended depth of focus (EDF) using 5 layers with a distance of 6μm each. Subsequently, whole slide images were converted to JPEG2000 format or accessed via the ImageJ Microscope plugin for ROI selection and image export [16].

For double immunofluorescent staining of ZEB1 and IDH1 R132H, anti-mouse Alexa 488 conjugated and anti-rabbit Cy3 conjugated secondary antibodies (Dianova, 1:100) were used. For quantification, multichannel images were acquired from ten fields of view and nuclei were automatically identified using CellProfiler [17]. Image montages were generated for each nucleus and presented in a blinded fashion to the experimenter using custom ImageJ scripts for manual dichotomization. For mutant IDH1, a nuclear or nuclear envelope staining pattern was required for positivity.

### Fluorescence in situ hybridization

Fluorescence in situ hybridization was performed using Vysis EGFR/CEP-7 FISH probes (Abbott Molecular, 01N35-020) and Histology FISH Accessory Kit (Dako, K579911), following the manufacturer’s protocol. Briefly, slides were incubated in pre-treatment solution at 95–99°C for 10 minutes and digested with pepsin for 5 minutes before adding the FISH probe, followed by denaturation (66°C, 10 min) and hybridization (45°C, 120 min). Slides were counterstained with DAPI for 5 minutes.

### Tissue microarray analysis

Individual spot images were extracted from whole slide images using a custom ImageJ gridding plugin. A region of interest (ROI) was defined automatically by determining the largest continuous non-white area using custom ImageJ scripts and refined manually to exclude artifacts and necrotic areas for each spot. For ZEB1 nuclear staining, ROIs were subjected to automated object recognition of single nuclei followed by intensity threshold based classification using CellProfiler [17]. Quantification of Ki67 and p53 stainings was performed by determining the area fraction of DAB positive cells using the ImmunoRatio ImageJ plugin [18]. For EGFR cytoplasmic staining, the mean intensity of the entire ROI was calculated. Manual scoring of EGFR amplification by FISH and pEGFR expression has been published before [11]. IDH1 mutation status was determined manually based upon IDH1 R132H specific antibody staining. The median of three replicate tissue spots was used for all graphs.

### Transcriptome sequencing and GBM subtype calling

RNA sequencing has been performed previously to generate transcriptomic profiles [12]. Briefly, mRNA was purified using poly(A) selection and strand-specific libraries were prepped for HiSeq 2000 paired-end sequencing (Illumina). After sample demultiplexing, reads were aligned to the hg19 reference genome using TopHat, version 1.4.1, [19] and gene expression estimates were calculated using cufflinks, version 2.0.2 [20]. To assign a gene expression subtype to the parental tumor of each cell line, we used the original 840 gene signature proposed by Verhaak and colleagues, where each subtype (Classical, Mesenchymal, Neural, and Proneural) is represented by a set of 210 distinguishing genes [21]. After performing a z-score transformation on FPKM gene-level expression values, samples were subjected to single sample gene set enrichment analysis (ssGSEA) as implemented by the ssGSEAProjection algorithm in the GenePattern software suite [22]. In ssGSEA, an enrichment score is calculated for each of the classification gene sets and samples were assigned to the subtype with the highest score [23].

### Quantitative realtime PCR

Total RNA was isolated from cells using RNeasy columns (Qiagen, Hilden, Germany) according to the manufacturer's instructions and quantified spectrometrically. 100–500 ng RNA were reverse-transcribed by 200 units MuLV reverse transcriptase (Promega) using random hexamer and oligo dT primers in 25 μl total reaction volume.

Real-time PCR was performed on a realplex epGradient S PCR cycler (Eppendorf). For a 25 μl reaction, 12.5 μl 2X QuantiTect SYBRGreen mastermix (Qiagen), 0.8 μl of a 10 μM forward and reverse primer mix and 2 μl template were used. 40 cycles of 15 s denaturation at 94°C, 30 s annealing at the temperature indicated below and 30 s extension at 72°C were performed. Melting curve analysis was used to exclude primer-dimer formation and ensure proper amplification. The following primer pairs were used: ZEB1 (F: GATGATGAATGCGAGTCAGATGC, R: CTGGTCCTCTTCAGGTGCC, T_A_ 60°C) [24], HPRT1 (F: TGAGGATTTGGAAAGGGTGT, R: GAGCACACAGAGGGCTACAA, T_A_ 58°C), and GAPDH (F: GGCATGGACTGTGGTCATGAG, R: TGCACCACCAACTGCTTAGC, T_A_ 58°C) [25]. Results were analyzed using the ∆∆Ct-method and normalized to the geometric mean of two housekeeping genes (HPRT1, GAPDH) [26]. Reactions were performed in duplicates and three independent experiments were performed from different passages per cell line.

### Immunocytochemistry and image cytometry

10^4^ cells were seeded on laminin-coated chamber slides (ibidi) and allowed to attach for 24 hours. Cells were fixed with 4% paraformaldehyde for 10min (PFA, Sigma), permeabilized for 4min in 0.5% Triton-X 100 (Carl Roth) and blocked for 15min in 5% serum from the secondary antibody species. Rabbit polycloncal anti-ZEB1 antibody (HPA027524, Sigma-Aldrich, dilution 1:100) and, for detection, Alexa 647-conjugated secondary antibodies (Life Technologies, dilution 1:500) were incubated for 45 min at 37°C. Nuclei were counterstained with DAPI at room temperature for 5 min. Imaging was performed using an IX81 inverted fluorescence microscope (Olympus).

For quantification, 25 fields of view were imaged for each condition using a motorized stage. After illumination field correction, nuclei were detected in the DAPI channel and the mean intensity of each nucleus in the ZEB1 channel was determined using CellProfiler [17].

### Statistics

To study associations of ZEB1 with molecular and clinical parameters, a random intercept linear mixed model was applied to account for the nested data structure of replicates nested in patients. The regression model was fitted to a subset of the data including all primary and secondary glioblastoma cases and considering patient sex, age, EGFR and IDH1 genomic alteration as well as p53 and EGFR expression as fixed factors. All three replicates entered the analysis as level one units, patients were level two units. For survival analysis, the log-rank test was used to test for group differences. All statistical analyses were performed using R with lme4 and lmerTest packages [27].

### Reproducibility and data availability

All raw data from IHC quantification are provided in the Supplementary Material. Raw sequencing data from RNA-seq experiments are available at the European Genome-phenome Archive (http://www.ebi.ac.uk/ega, accession EGAS000010011679). ImageJ plugins for processing of TMA virtual slides are available at https://gitlab.com/pesk/TMAtools.

## Results

### ZEB1 is widely expressed in human gliomas

We analyzed protein expression of ZEB1 by immunohistochemistry in a panel of human gliomas of different WHO grade and a tissue microarray (TMA) of 243 high grade glioma samples and performed an unbiased, single-cell level classification **(S1 Table)**.

**Fig 1:**
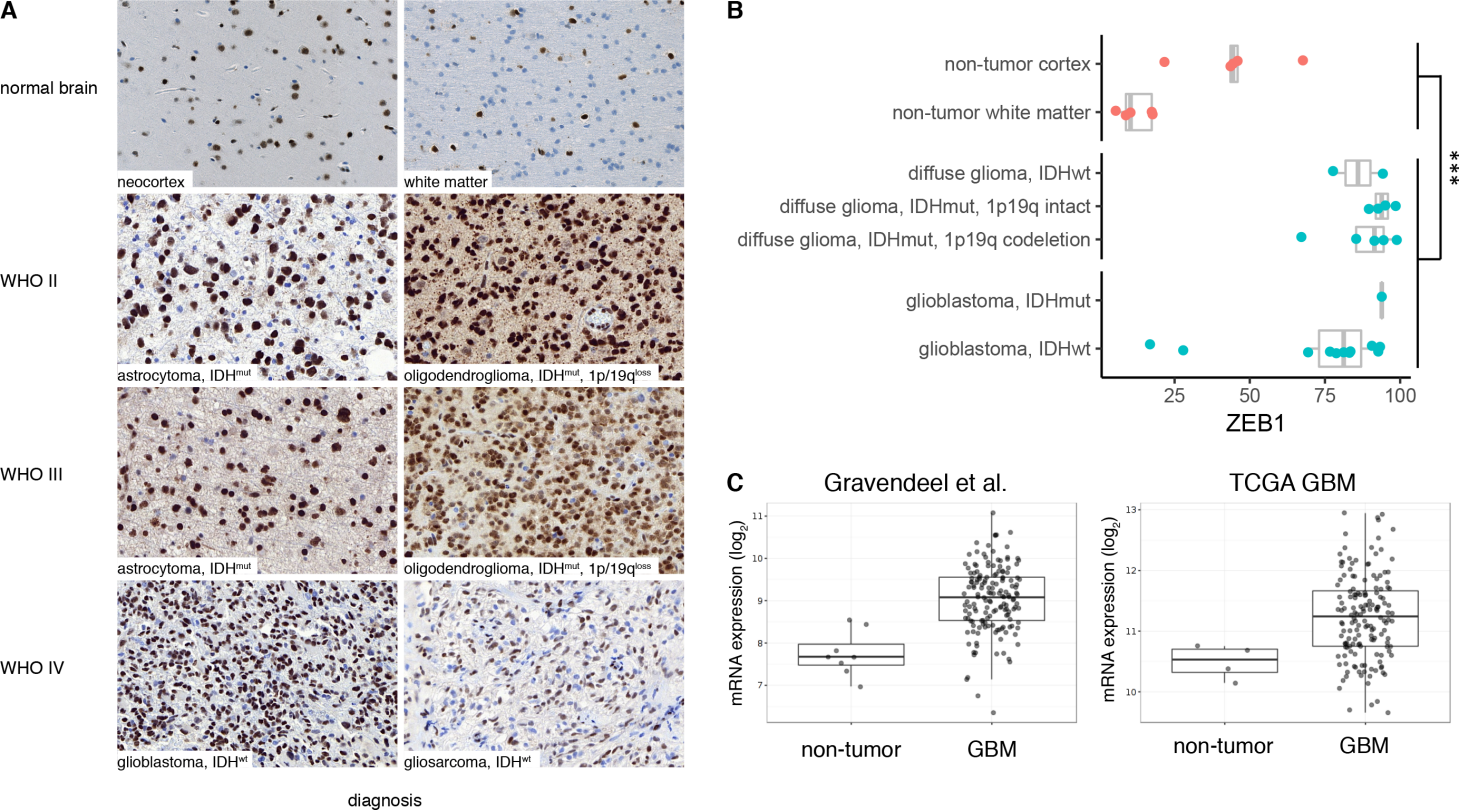
Expression of ZEB1 in human glial tumors and normal brain. (A) Biopsy samples of human glial tumors show abundant nuclear expression of ZEB1. Sections from non-neoplastic normal brain show weak cytoplasmic staining of neurons and nuclear expression in astrocytes. (B) Quantification of ZEB1 in full histological sections of gross total resections of human glial tumors. ZEB1 positive cells were quantified using automated image analysis. Dots represent the mean of multiple regions of interest (ROI) per tumor. Box plots show the distribution of the ZEB1 labelling in tumor with indicated integrated diagnosis according the 2016 WHO classification of CNS tumors [29]. (C) *ZEB1* mRNA expression in public GBM cDNA microarray datasets. Boxplots show distribution in normal brain vs. GBM. Data were retrieved via the GlioVis portal [30].

First, we investigated full histological sections of gross total resections (N = 23) of gliomas with different IDH and chromosome 1p/19q codeletion status and normal brain controls (N = 5). We found widespread expression of ZEB1 in all tumor entities **(Fig 1A)** and observed a dichotomous nuclear expression pattern identifying ZEB1-positive and ZEB1-negative populations in all tumors. In non-tumor brain samples obtained from epilepsy surgery, we found that the ZEB1-positive fraction is expression is significantly lower (linear regression, p = 0.0005), especially in normal white matter where glial tumor usually reside **(Fig 1B)**. As a validation, we compared ZEB1 expression in gliomas vs. normal brain tumors in public transcriptomic datasets from several studies [28] and found higher *ZEB1* mRNA expression in glial tumors compared to normal brain controls **(Fig 1C)**. The relative fraction of ZEB1-positive cells, however, varied markedly across tumors with higher variability observed in GBM **(Fig 1B)**.

### Intertumoral heterogeneity of ZEB1 expression in glioblastoma

To further characterize the observed heterogeneity of ZEB1 expression in GBM, we analyzed a tissue microarray of GBM samples complemented with molecular markers, quantification of routine histology and clinical parameters **(S2 Table)**. We determined the percentage of ZEB1^+^ cells (ZEB1 labelling index) from three cores per tumor with an average of 3,693 nuclei (min. 133; max. 9,485) quantified per core.

**Fig 2:**
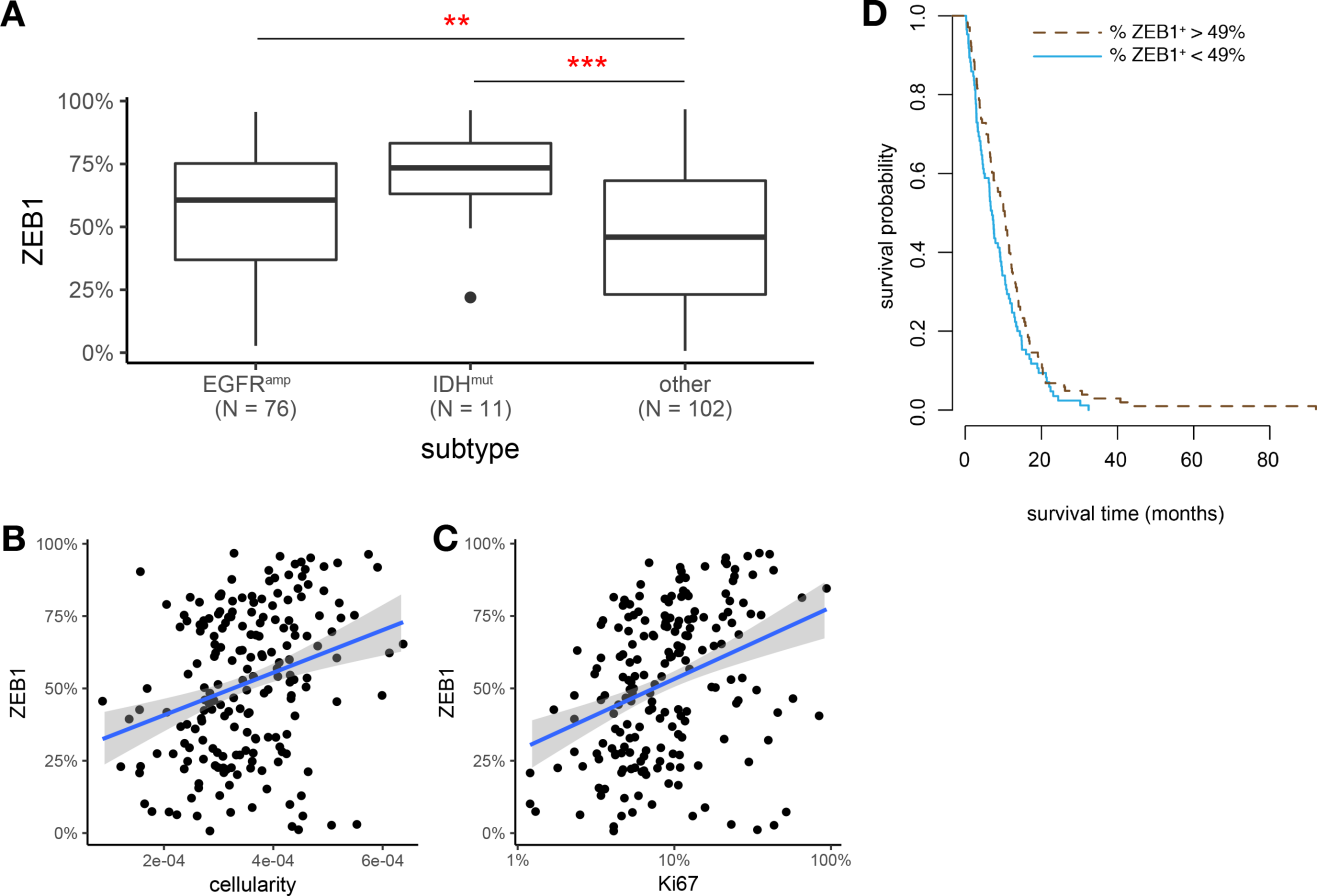
Intertumoral heterogeneity of ZEB1 expression. (A) Box plots of ZEB1 labelling index (percent) with respect to EGFR amplification and IDH1 mutation. (B,C) Correlation of ZEB1 expression and Ki67 labelling index or cellularity, respectively, in N = 193 glioblastoma TMA samples. Trend lines indicate linear regression estimates. Note log scale for Ki67 index. (D) Kaplan-Meier estimates of overall survival time (months) with respect to ZEB1 expression. The observed data range of ZEB1^+^ percentages was split into two equally sized bins at the threshold value of 49%.

To identify GBM of the classical and (G-CIMP+) proneural gene expression subtypes [21,31], we stratified samples with respect to EGFR amplification and IDH mutations. ZEB1 labelling index showed pronounced differences with respect to these subgroups **(Fig 2A)**. A multivariate analysis confirmed that both EGFR expression and IDH1 status independently and significantly predict higher ZEB1 labelling index **(S3 Table)**. Interestingly, similar results were found for bulk ZEB1 mRNA expression data in microarray based gene expression data from the TCGA GBM cohort (S1 Fig).

ZEB1 labelling index was significantly and positively correlated with Ki67 proliferation index (Pearson's r = 0.21, p = 0.003) and cellularity (Pearson's r = 0.28, p < 0.0001) **(Fig 2B and 2C)**. In analogy to the well-established concept of ZEB1 as a marker of disease progression in carcinomas, we performed survival analysis separating the dataset into tumors with low versus high ZEB1 labelling index **(Fig 2D)**. However, no significant difference in overall survival was found (p = 0.067), with Kaplan-Meier estimates of median survival being 7.1 months for ZEB1^low^ tumor and 10.4 months for ZEB1^high^ tumors.

### Intratumoral heterogeneity of ZEB1 expression in glioblastoma

We then investigated how intratumoral heterogeneity contributes to ZEB1 expression and how it might explain the observed group differences with respect to EGFR and IDH status. Solid tumors are cellularily heterogeneous, both within the tumor cell population and with respect to composition of the cellular microenvironment.

**Fig 3:**
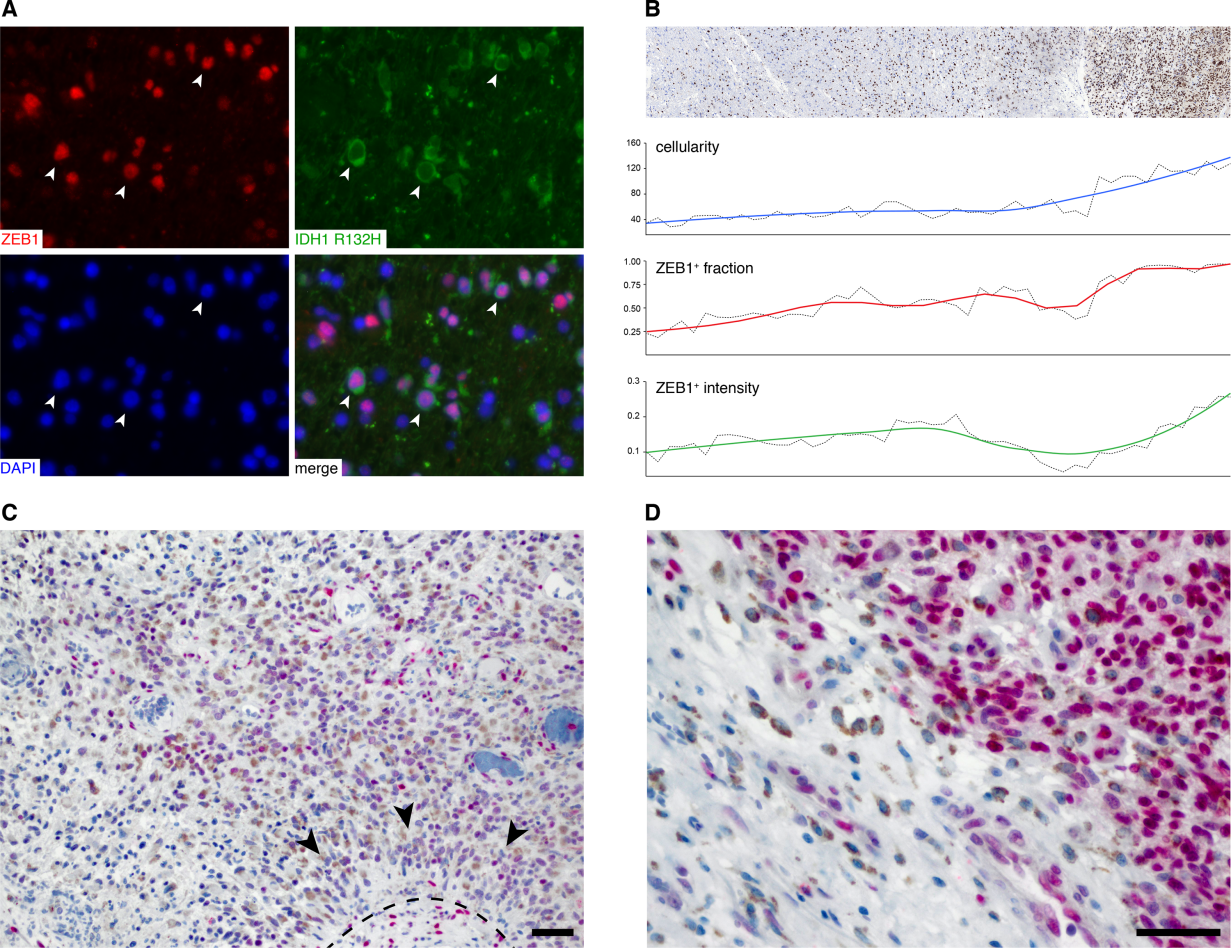
Intratumoral heterogeneity of ZEB1 expression. (A) Co-labeling of IDH1 R132H and ZEB1 in a case of IDH1 mutant glioblastoma. The image shown has been taken from one out of ten fields of view that were subjected to separate and blinded manual scoring of mutant IDH1 and ZEB1 expression. (B) ZEB1 gradient along the tumor edge of a mesenchymal GBM (BLN-7 parental tumor). ZEB1 IHC, overall cellularity (*blue*), the relative frequency of ZEB1+ cells (*red*) and the mean nuclear intensity of ZEB1+ cells (*green*) at the tumor edge are shown. (C,D) Expression of ZEB1 (*pink*) and CD68 (*brown*) in perinecrotic regions with (C) or without (D) pseudopalisades.

First, we wondered if ZEB1 is expressed by all tumor cells. Currently, mutation specific IDH1 immunohistochemistry is the only available true *in situ* tumor cell marker for gliomas. We therefore performed immunofluorescent double labeling of ZEB1 and IDH1 R132H in four IDH-mutant GBM cases **(Fig 3A, S2 Fig)** and quantified colocalization after blinded manual scoring of individual nuclei from at least three fields of views per tumor **(S1 File)**. Indeed, 96.3% (95% confidence interval 95.0%–97.3%) of IDH1 mutant cells also stained positive for ZEB1. It is thus likely that nearly all tumor cells stain positive for ZEB1. In contrast, we observed variable staining intensities in ZEB1^+^ tumor cells (Fig 3A). Since increased ZEB1 expression by tumor cells has been associated with hypoxia and invasion, we analyzed regional ZEB1 expression when approaching the tumor edge **(Fig 3B)** or in perinecrotic areas **(Fig 3C and 3D)**. We identified the tumor border of a GBM resection sample and quantified cellularity and the relative fraction of ZEB1 positive cells along a radial axis perpendicular to the tumor edge. Single ZEB1^+^ cells scatter in the periphery of the tumor and their relative numbers (i.e. the percentage of ZEB1^+^ cells) increase simultaneously with overall cellularity **(Fig 3B, *blue*)** when approaching the tumor core **(Fig 3B, *red*)**. As higher ZEB1 expression has been suggested in migrating tumor cells [9], we also analyzed the mean staining intensity of each ZEB+ nucleus with respect to location **(Fig 3B, *green*)**. Unexpectedly, ZEB1 staining intensity increased towards the tumor center. Even though DAB immunohistochemistry is a semi-quantitative method at best [32], the results do not indicate increased expression at the tumor edge. Next, we evaluated ZEB1 expression by tumor cells near in pseudopalisades and perinecrotic areas from seven additional cases in colabeling with CD68 to discriminate macrophages populating the necrotic region **(Fig 3C and 3D)**. While some interspersed cells showing very high ZEB1 staining intensity could be observed, no general pattern of ZEB1 intensity with respect to proximity to necrosis could be appreciated. In summary, ZEB1 is expressed by nearly all tumor cells but a varying expression levels.

### ZEB1 expression *in vitro*

**Fig 4:**
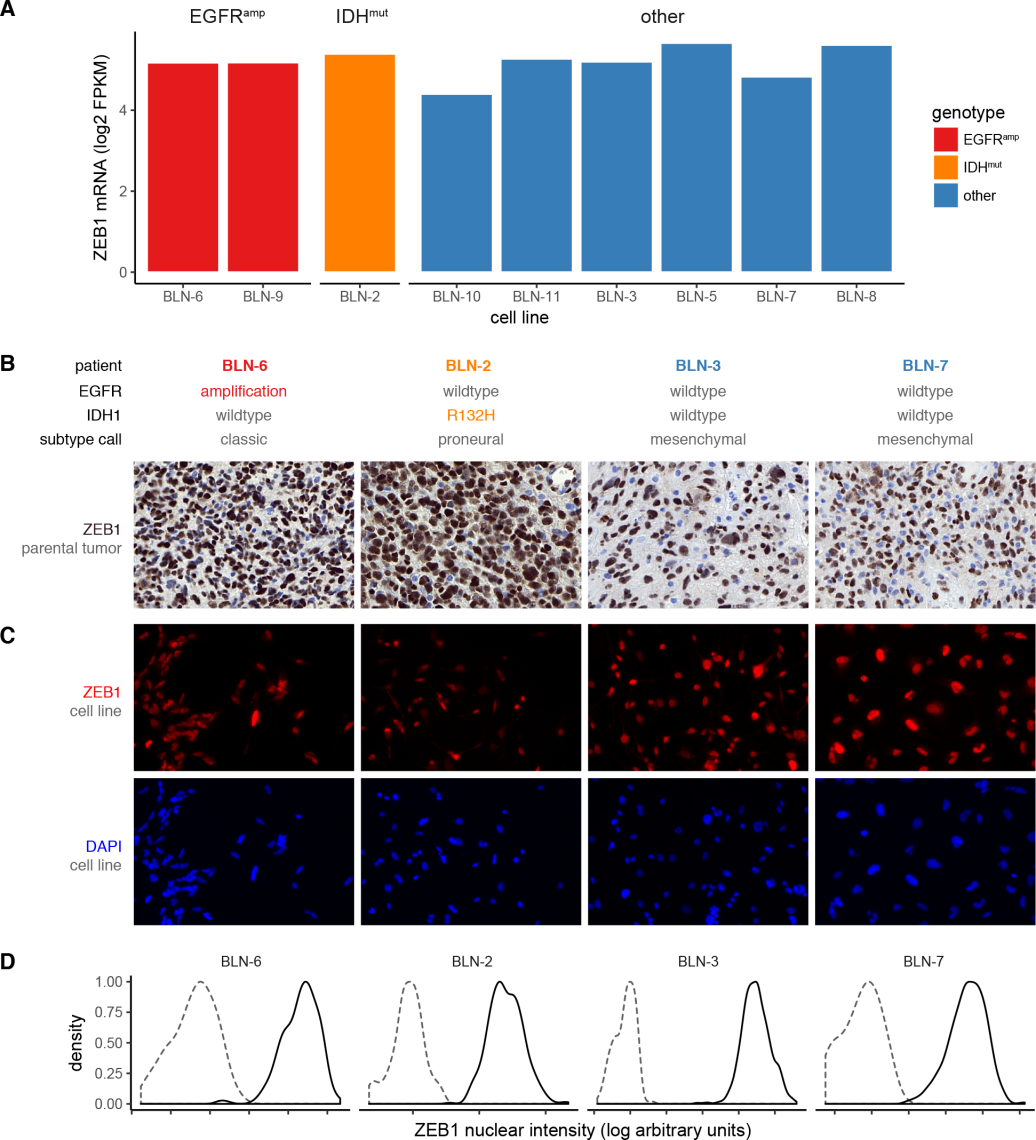
Expression of ZEB1 in glioma stem cell lines. (A) *ZEB1* mRNA expression in gliomasphere cell lines with known EGFR and IDH1 status of the parental tumor and cell line. Expression levels from RNA-seq are given as log_2_-transformed fragments per kilobase per million reads (RPKM). (B,C,D) ZEB1 protein expression in cell lines and matched parental tumors. Human GBMs (B) and matched glioma stem cell lines (C) were stained for ZEB1 using immunostaining. (D) The fraction of ZEB1-positive cells was quantified using image cytometry. Gating was performed with respect to a secondary antibody only negative control. Histograms show a representative intensity distribution of the negative control (*dashed*) and ZEB1 staining (*solid*) from at least three independent experiments.

To overcome the limitation of missing tumor markers for IDH-wildtype glioblastoma, we next analyzed a panel of nine patient-matched gliomasphere cell lines [12] to further test differences in ZEB1 expression within a pure population of tumor cells. We first investigated bulk mRNA expression levels respect to EGFR and IDH status **(Fig 4A)**. All cell lines expressed ZEB1 on the transcriptional level irrespective of genotype. We then selected four cell lines for in depth study of ZEB1 protein expression on a single cell level. Irrespective of EGFR or IDH status or the observed heterogeneity of ZEB1 in the parental tumor **(Fig 4B, S3 Fig)**, ZEB1 was homogeneously expressed in virtually all cells of all four cell lines **(Fig 4C)**, which could be quantified by image cytometry **(Fig 4D)**. We thus conclude that ZEB1 is robustly expressed in glial tumor cells.

### ZEB1 expression in the tumor microenvironment

**Fig 5:**
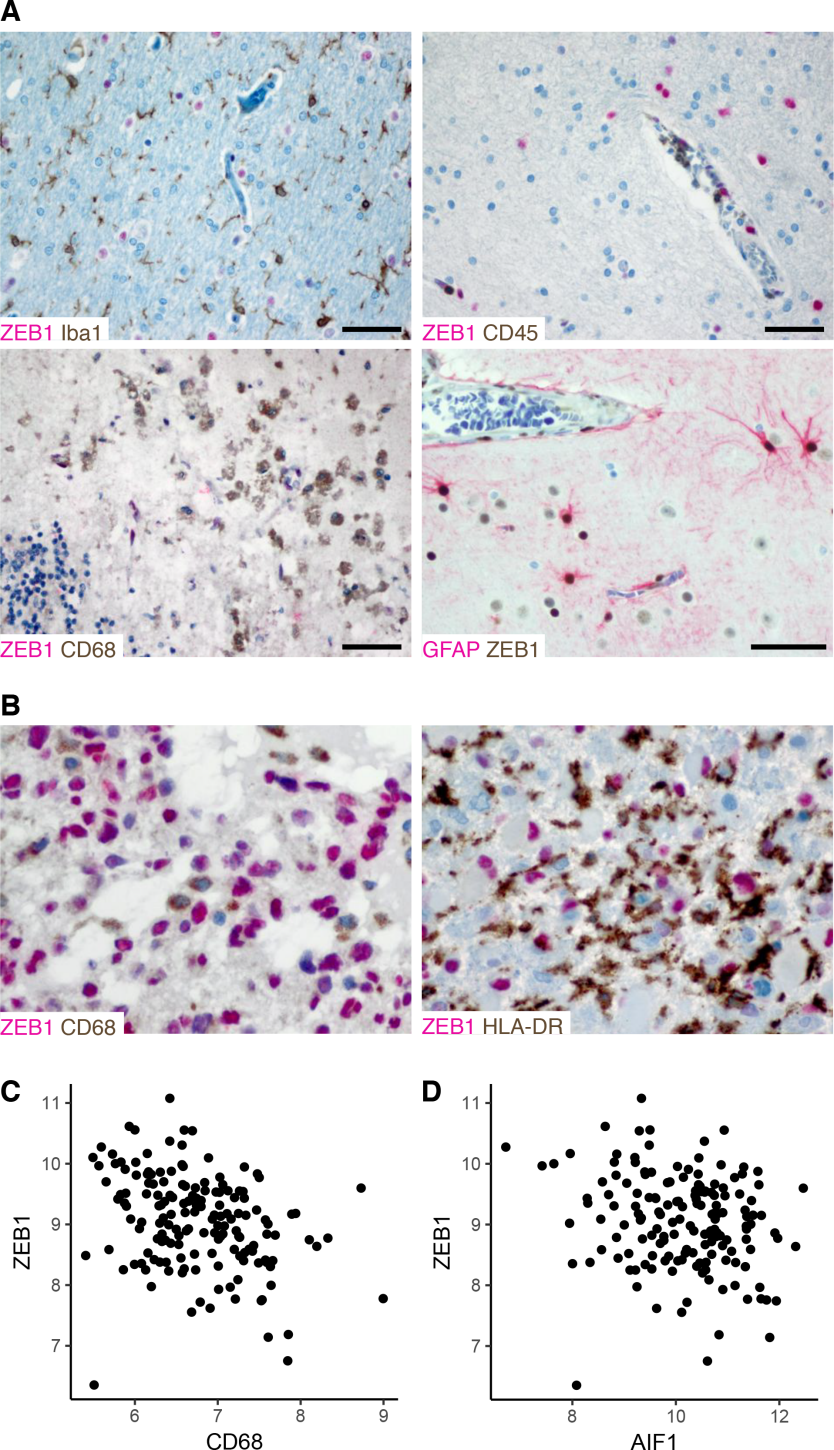
ZEB1 expression in the tumor microenvironment. (A) Cell type specific expression of ZEB1 in reactive brain tissue. Human brain biopsy samples from cases of seizure-induced reactive gliosis or subacute infarction were co-stained for ZEB1 and Iba1 for microglia, CD45 for leucocytes, CD68 for macrophages and GFAP for astrocytes, respectively. Scale bars 50 μm. (B) Co-labeling of ZEB1 and CD68 or HLA-DR, respectively in human GBM. (C,D) Correlation of microarray-based gene expression levels of *ZEB1* and myeloid cell markers *CD68* (C) and *AIF1* (D), respectively. Processed log_2_-transformed intensities from 157 GBM cases studied by Gravendeel et al. [34] were obtained via the GlioVis portal [30].

Finally, to test whether ZEB1 is expressed by cells of the tumor microenvironment, co-staining of ZEB1 with markers of immune and glial cells were performed in non-tumor tissue. Macrophage (CD68), microglia (Iba1) and leucocytes (CD45) markers revealed mutually exclusive patterns with ZEB1 **(Fig 5A)**. In contrast, reactive astrocytes and endothelial cells showed strong ZEB1 positivity, and some neurons showed weak ZEB1 expression **(Fig 5A)**. Thus both tumor cells and reactive astrocytes, but not immune cells stain ZEB1-positive. Since tumor-associated microglia and macrophages are the most abundant population of immune cells found in GBM [33], we further investigated mutual exclusivity of ZEB1 with CD68 or HLA-DR (as a marker of activated microglia and macrophages) in additional GBM cases (N = 8) using co-labeling **(Fig 5B)**. As in non-neoplastic tissue, tumor-infiltrating CD68^+^ or HLA-DR^+^ cells were consistently negative for ZEB1, too. Thus, given this mutual exclusivity of ZEB1 expression in tumor vs. immune cells, the amount of immune infiltration will invariably be reflected in the overall ZEB1 labelling index of human GBMs.

To corroborate this conclusion, we correlated bulk ZEB1 mRNA levels from a public GBM dataset [34] with expression of macrophage/microglial markers **(Fig 5C and 5D)**. For both CD68 and AIF1 (i.e. Iba1), we found significant negative correlations (Pearson's *r* = –0.33, p < 3 × 10^−5^ and *r* = –0.21, p = 0.007, respectively). Thus, indeed, immune infiltration is anti-correlated with bulk ZEB1 expression.

## Discussion

The current study provides an *in situ* characterization of ZEB1 at single cell resolution in human gliomas. With our focus on glioblastoma, we show high intra- und interindividual variation of the ZEB1 labeling index and an association with EGFR amplification and IDH mutation, i.e. molecular traits of the classical and proneural subtypes, respectively. Many factors are likely to contribute to these variable ZEB1 expression levels, both intratumorally and intertumorally. Tumor cells with EGFR amplification [9], those in the process of migration or exposed to hypoxia [7,8] all have been shown to express higher levels of ZEB1 than controls and experimental studies have identified molecular mechanisms involved in ZEB1 regulation, such as PDGFRA, Wnt- or TGF-beta signaling [6,35–37]. Here, we show that the cellular composition of solid tumors contributes to this heterogeneity. While tumor cells in glioma consistently express ZEB1, we observed mutually exclusive staining with markers of immune cells (CD45, CD68, Iba1, HLA-DR). This is important for interpretation of ZEB1 gene and protein expression of bulk tumors with immune infiltration being a confounder which is tightly associated with GBM subtypes.

It has previously been reported that ZEB1 expression is higher in IDH mutant lower grade glioma [38]. In our study, we have only analyzed a small number of diffuse astrocytomas and oligodendrogliomas, which precluded statistical testing, but we observed the same trend. Another study did not find an association between ZEB1 expression and EGFR amplification or IDH mutation, but compared bulk tumor levels [39].

As we show in IDH-mutant glioblastomas and glioma cell lines, however, ZEB1 appears to be expressed uniformly by the entire tumor cell population. This surprisingly ubiquitous and robust expression pattern of ZEB1 in glial tumors challenges the canonical concept of ZEB1 as a marker of EMT and tumor progression. The numerous biological contexts that have recently been identified for ZEB1 to be involved in, such as immunosuppression and DNA repair [4,5], also suggest that its function is not restricted to an EMT signaling network.

The results also challenge the findings studies claiming that ZEB1 is expressed only in about half of the examined cases, predominantly at the invasive edge or that survival negatively correlated with ZEB1 expression [8,9]. In more than 200 tumors, we found nearly ubiquitous expression in tissue microarray samples where spots represent selected subsamples from the tumor core. In gross total resections, we rather observed decreasing ZEB1+ percentage when approaching the tumor edge. We could also detect no significant differences in survival. However, there was a trend towards longer overall survival time in tumors with high ZEB1 labelling index suggesting that ZEB1 might be, if at all, a positive predictor of survival. In contrast, our data support findings from a recent study that suggest widespread co-expression of SOX2, OLIG2 and ZEB1 in glioblastoma, irrespective of key driver alterations [36].

Differential amounts of infiltrating immune cells more likely explain the observed heterogeneity of the ZEB1 labelling index. Even though a recent study suggests an enrichment in expression of EMT-related genes in tumor-associated macrophages in gliomas [40], we showed that immune cells are consistently ZEB1-negative. As microglia and tumor-associated macrophages have been shown to account for up to 30% of cells in glioma [41–44], they can thus substantially contribute to the ZEB1-negative population. This hypothesis is further supported by the notion that mesenchymal glioblastoma show higher immune infiltration than all other gene expression subtypes [21,45]. We found lower ZEB1 labelling indices in GBM without EGFR amplification or IDH1 mutation, which might be explained by the fact that this group will accordingly be enriched for mesenchymal subtype tumors with higher amounts of immune infiltration. Finally, we show in public gene expression data from bulk glioblastoma samples that ZEB1 inversely correlates with markers of microglia and macrophages. These findings have implications for the interpretation of previous studies investigating ZEB1 levels. For example, Neswick and colleagues found higher ZEB1 levels in IDH-mutant gliomas [38]. Since IDH mutations cause an immunosuppressive phenotype with lower numbers of lymphocytes and macrophages [46–48]. The correlation between ZEB1 expression and IDH status might thus be a spurious relationship mediated by immune infiltrates. This also means that the prognostic value of ZEB1 reported by the above-mentioned study might only be a surrogate of this relationship and care must be taken to attribute it to ZEB1 function.

In conclusion, our data support the notion of ZEB1 as universally expressed transcription factor in human glioblastoma with high inter- and intratumoral heterogeneity, which is best explained by differential contribution of the tumor microenvironment.

## Supporting information

S1 Fig ZEB1 mRNA expression in TCGA GBM cases with respect to transcriptional subtypes. Normalized and log2 transformed ZEB1 mRNA expression data from The Cancer Genome Atlas microarray studies are plotted with respect to gene expression subtype. The proneural group is further differentiated by CpG island methylator phenotype (G-CIMP). Horizontal bars indicate subtype median.
(TIFF)

S2 Fig Double-labeling of ZEB1 and mutant IDH1 in human glioblastomas. **Four cases of IDH-mutant GBM were subjected to blinded quantification of ZEB1 and IDH1 R132H status to determine the percentage of tumor cells staining positive for ZEB1.** In addition to the case shown in Fig 3A, representative images for from remaining cases is shown here.
(TIFF)

S3 Fig Characterization of parental tumors of GBM stem cell lines. (A) EGFR fluorescence in situ hybridization depicts chromosomal EGFR copies (*red*) and a chromosome 7 centromeric probe (*green*). (B) EGFR and (C) IDH1 R132H immunocytochemistry were counterstained with hematoxylin.
(TIFF)

S1 File Manual scoring results of ZEB1 and mutant IDH1 status from 1,270 nuclei from four independent tumors from at least three fields of view per tumor. (PDF)

S1 Table Overview of samples with quantifiable ZEB1 staining available. (DOC)

S2 Table Raw data of histological scoring, clinical and molecular annotation. (XLSX)

S3 Table Multivariate analysis by mixed model linear regression of ZEB1 labelling index with respect to molecular and clinical properties (n = 166 cases). (DOC)
