# Supplementary figures and images for: Cellular heterogeneity contributes to subtype-specific expression of ZEB1 in human glioblastoma
Source: PLoS One. 2017 Sep 25;12(9):e0185376. doi: 10.1371/journal.pone.0185376 (PMC5612763; doi:10.1371/journal.pone.0185376)

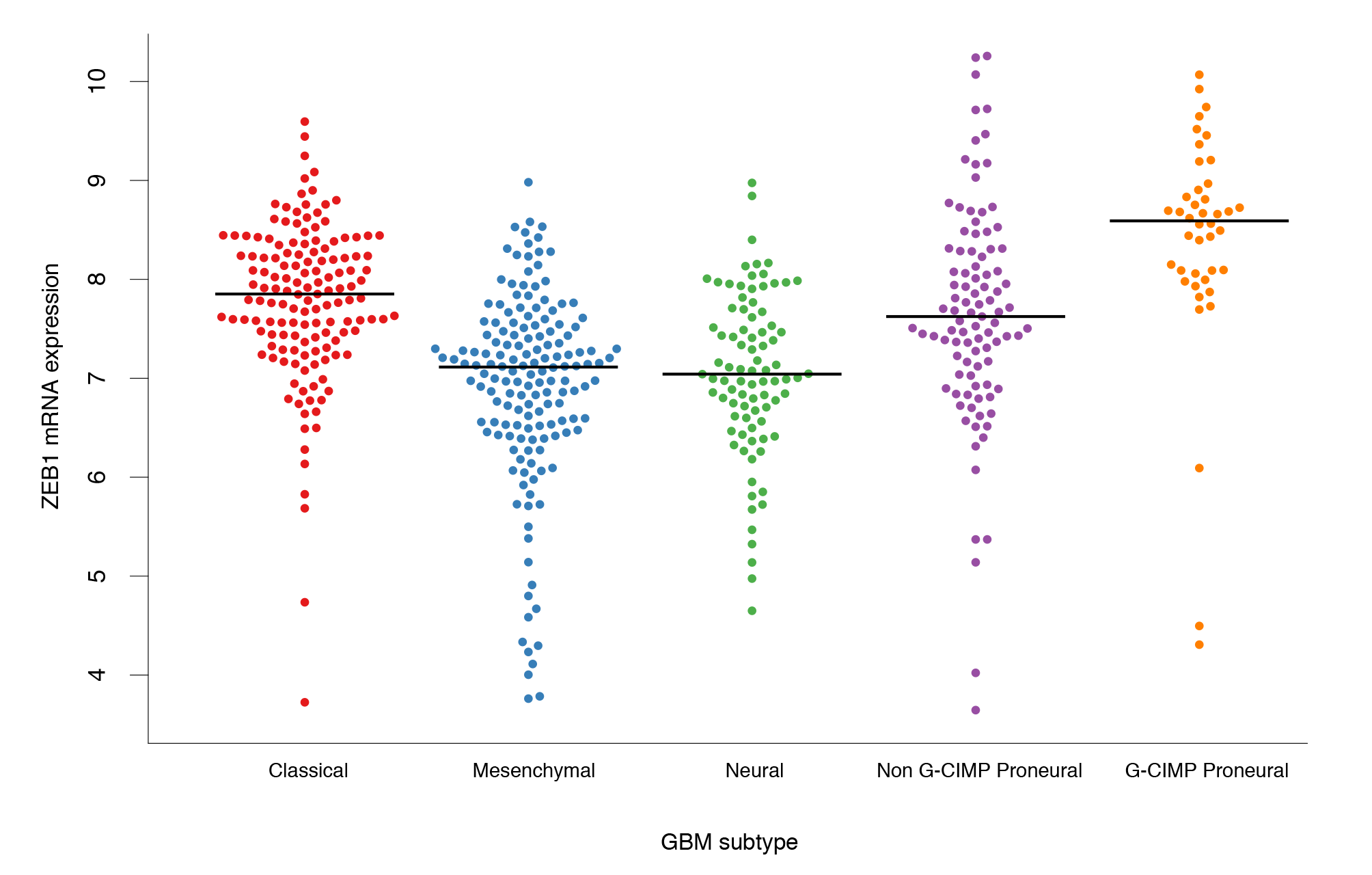

Supplement: S1 Fig — Normalized and log2 transformed ZEB1 mRNA expression data from The Cancer Genome Atlas microarray studies are plotted with respect to gene expression subtype. The proneural group is further differentiated by CpG island methylator phenotype (G-CIMP). Horizontal bars indicate subtype median. (TIFF) [file pone.0185376.s001.tiff]

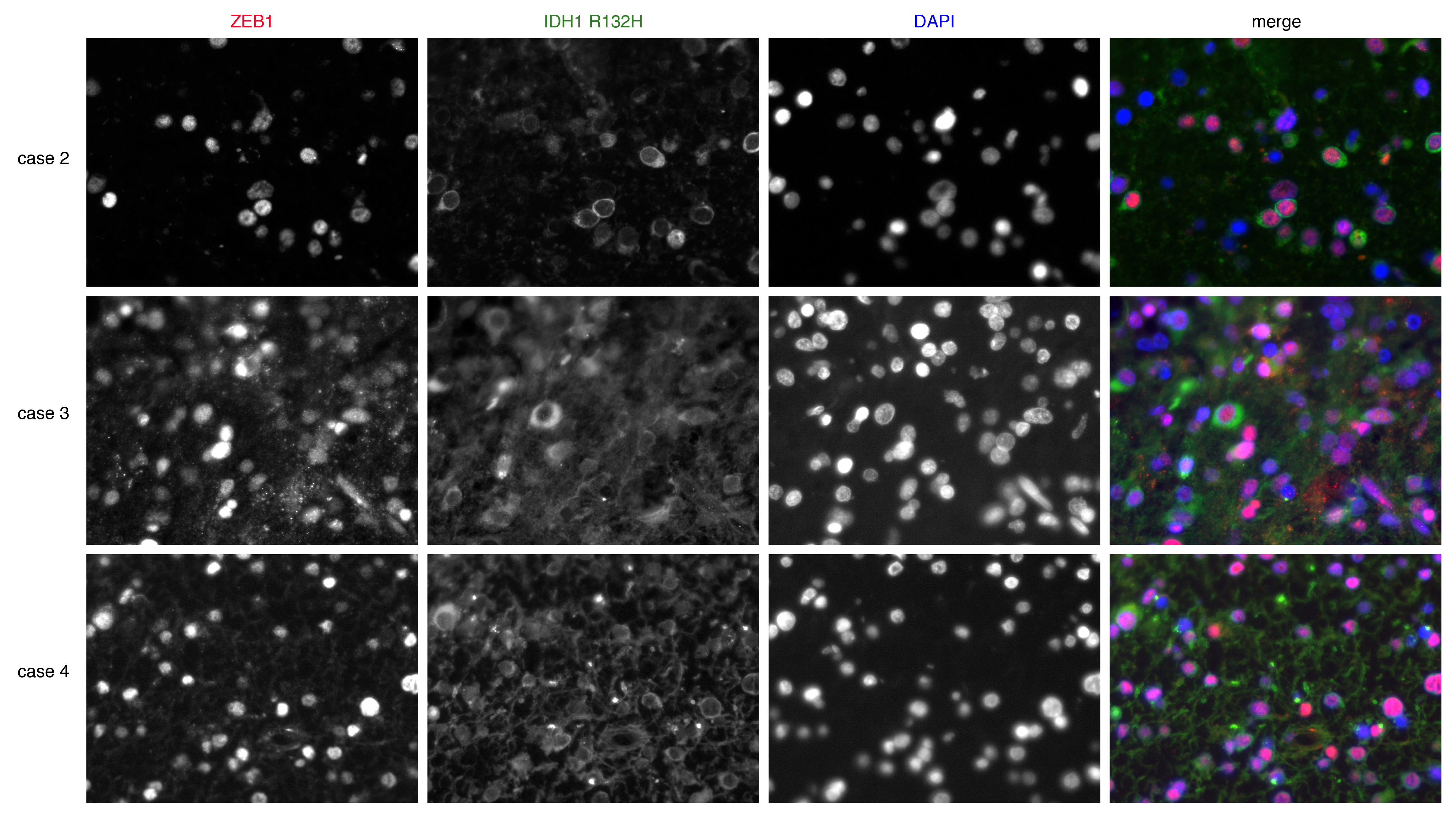

Supplement: S2 Fig — Four cases of IDH-mutant GBM were subjected to blinded quantification of ZEB1 and IDH1 R132H status to determine the percentage of tumor cells staining positive for ZEB1. In addition to the case shown in Fig 3A, representative images for from remaining cases is shown here. (TIFF) [file pone.0185376.s002.tiff]

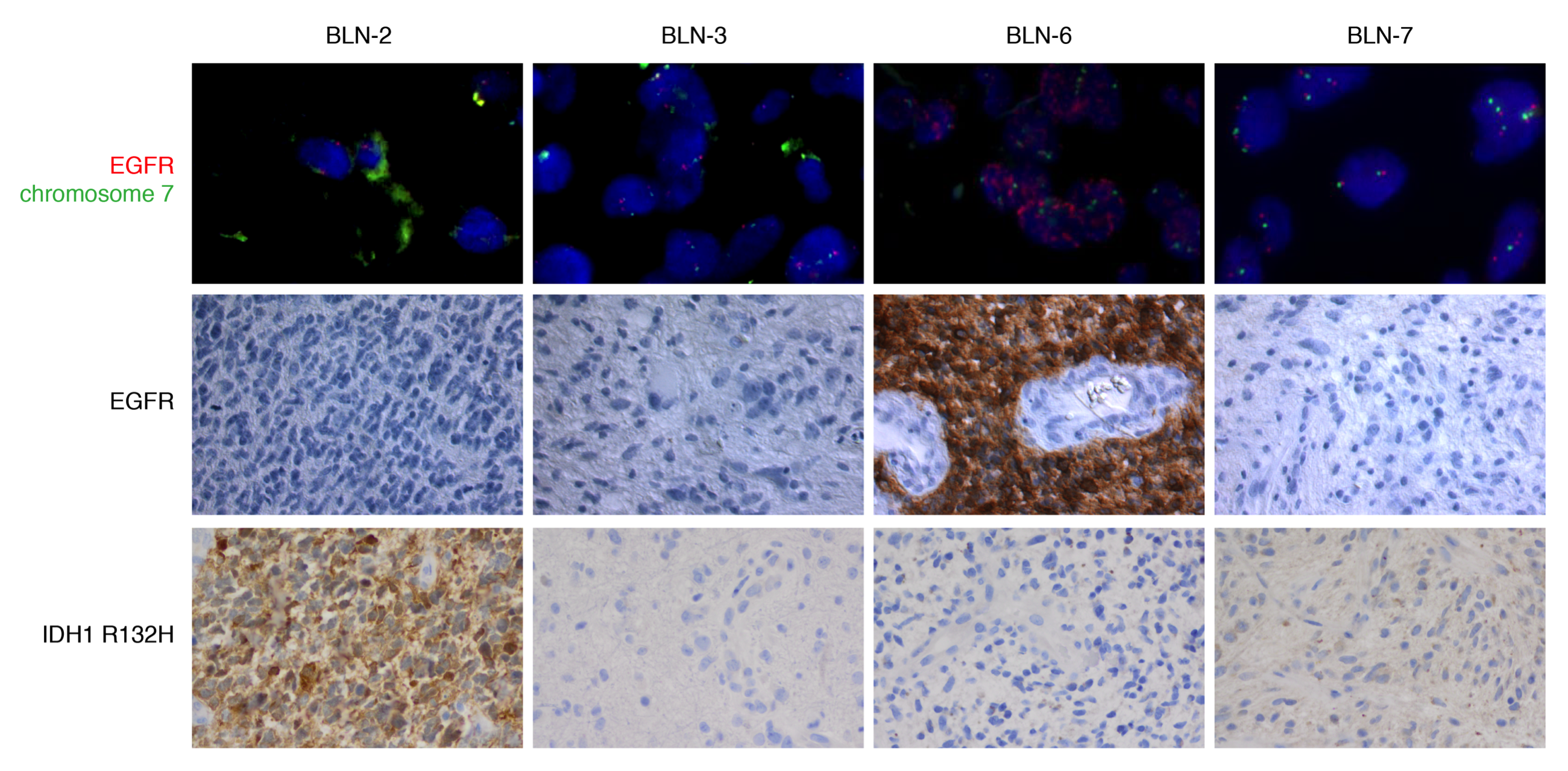

Supplement: S3 Fig — (A) EGFR fluorescence in situ hybridization depicts chromosomal EGFR copies (red) and a chromosome 7 centromeric probe (green). (B) EGFR and (C) IDH1 R132H immunocytochemistry were counterstained with hematoxylin. (TIFF) [file pone.0185376.s003.tiff]
