# Supplementary material for: Cellular heterogeneity contributes to subtype-specific expression of ZEB1 in human glioblastoma
Source: PLoS One. 2017 Sep 25;12(9):e0185376. doi: 10.1371/journal.pone.0185376 (PMC5612763; doi:10.1371/journal.pone.0185376)

Case1_ROI_1 overview

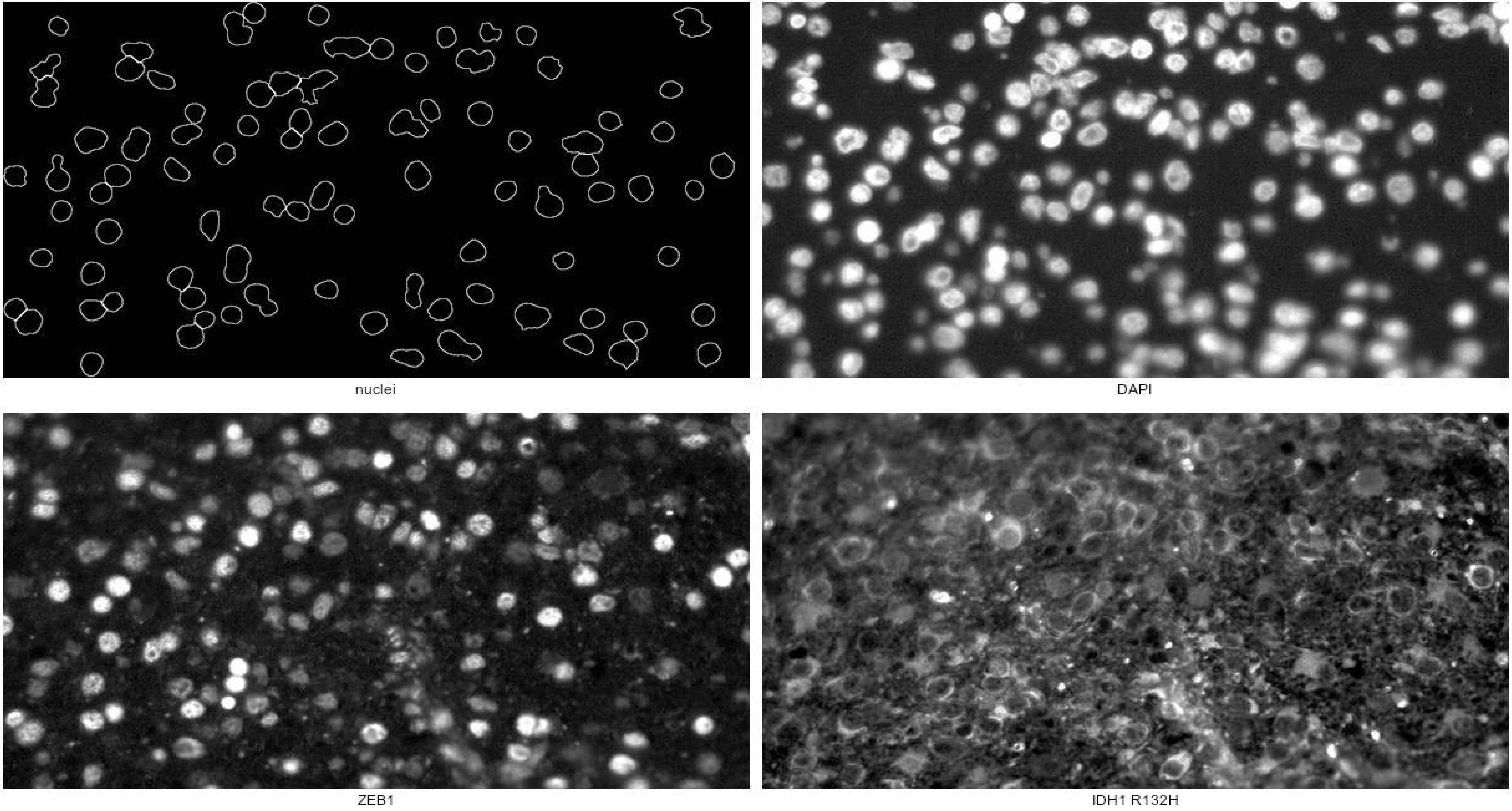

Case1_ROI_1 IDH1 scoring

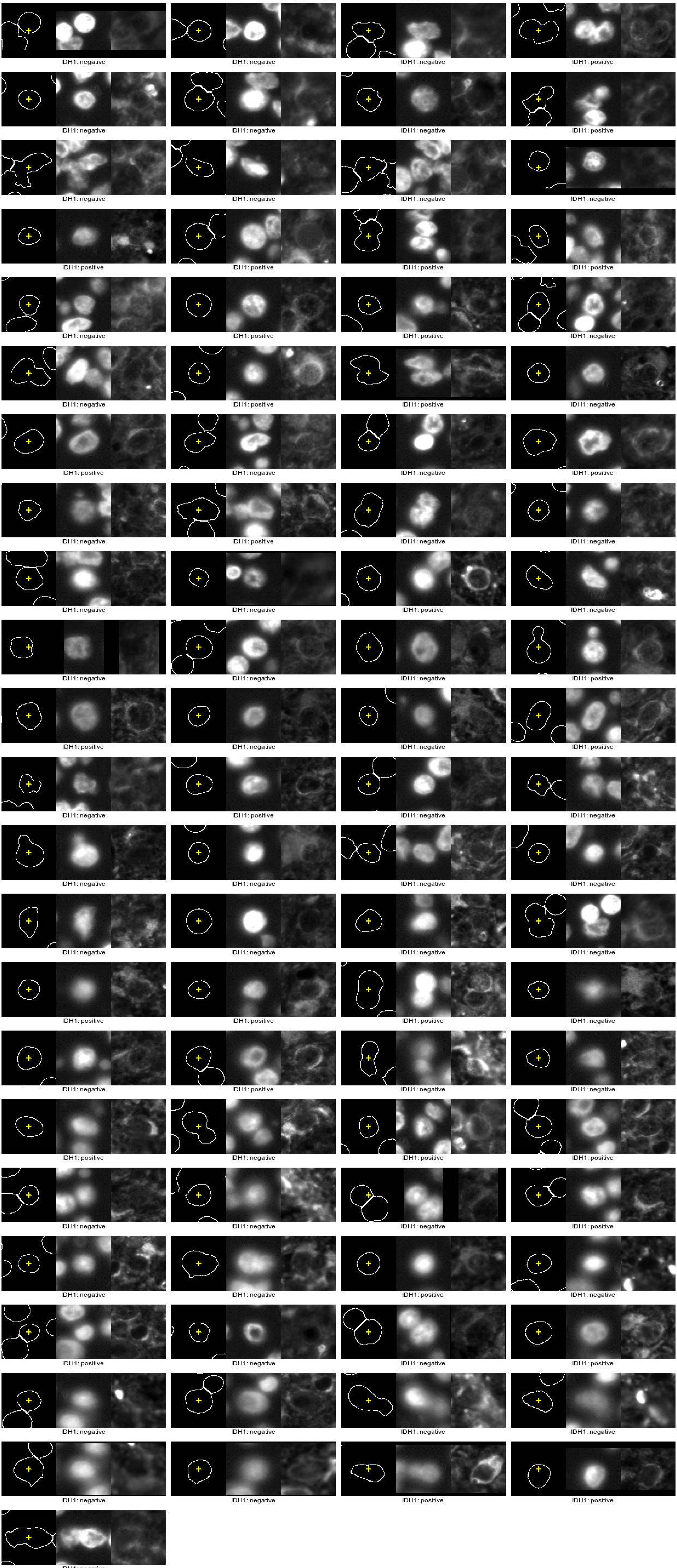

Case1_ROI_1 ZEB1 scoring

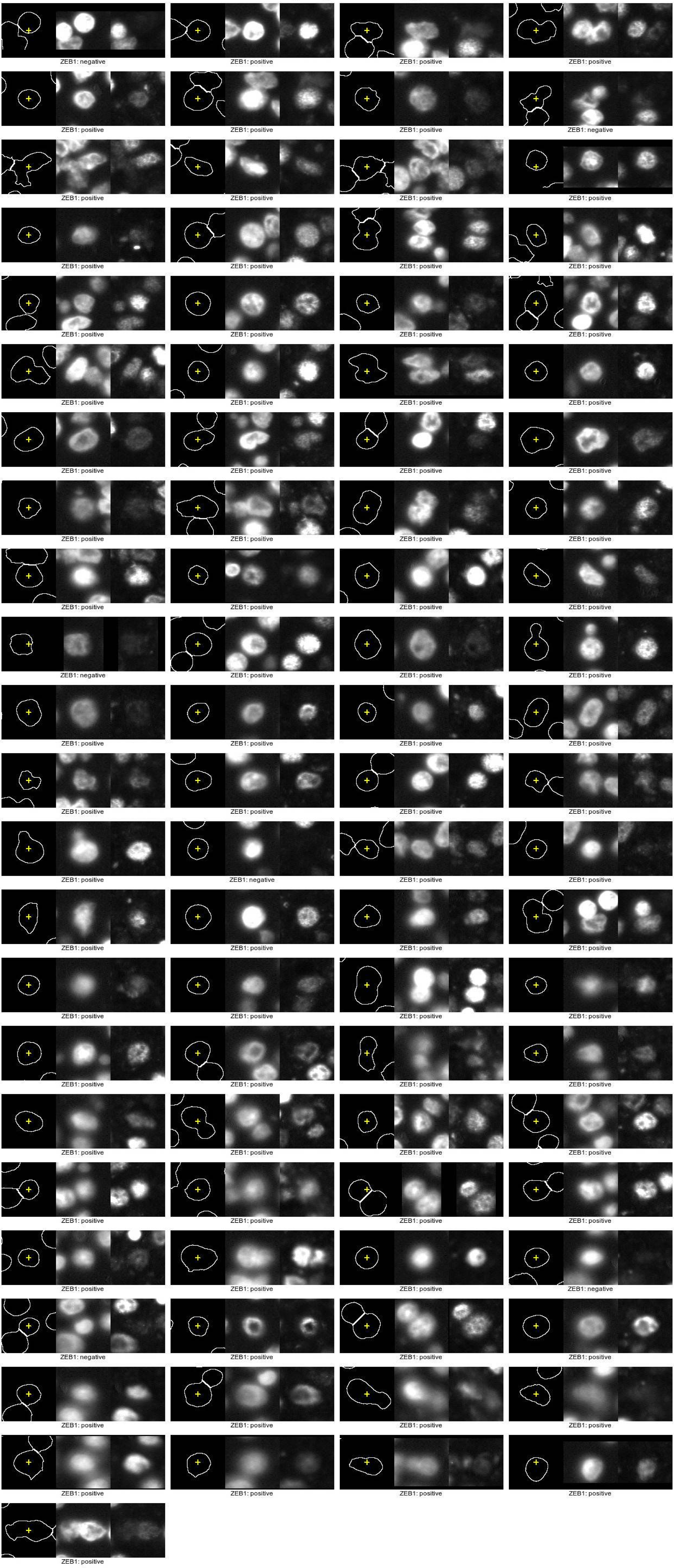

Case1_ROI_2 overview

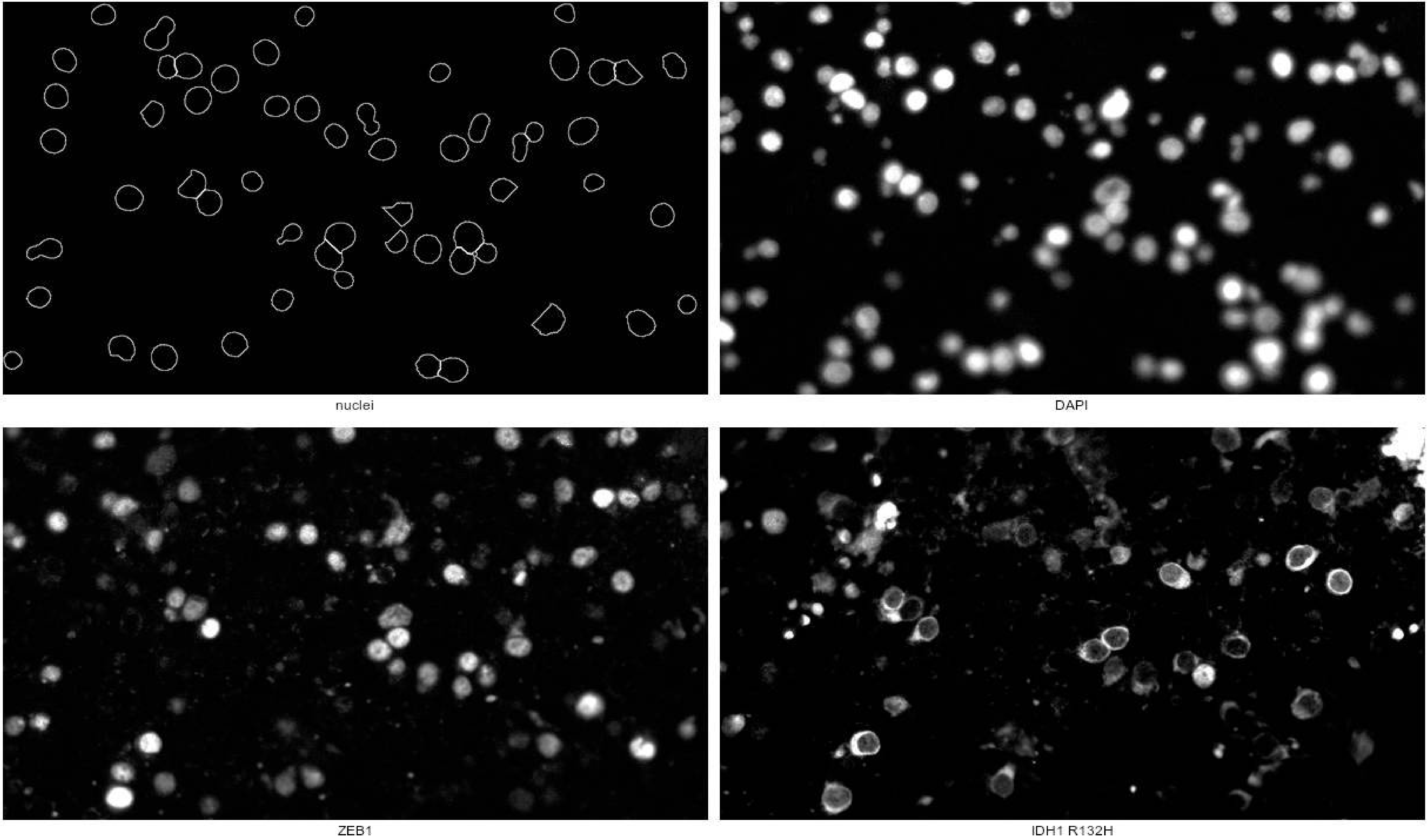

Case1_ROI_2 IDH1 scoring

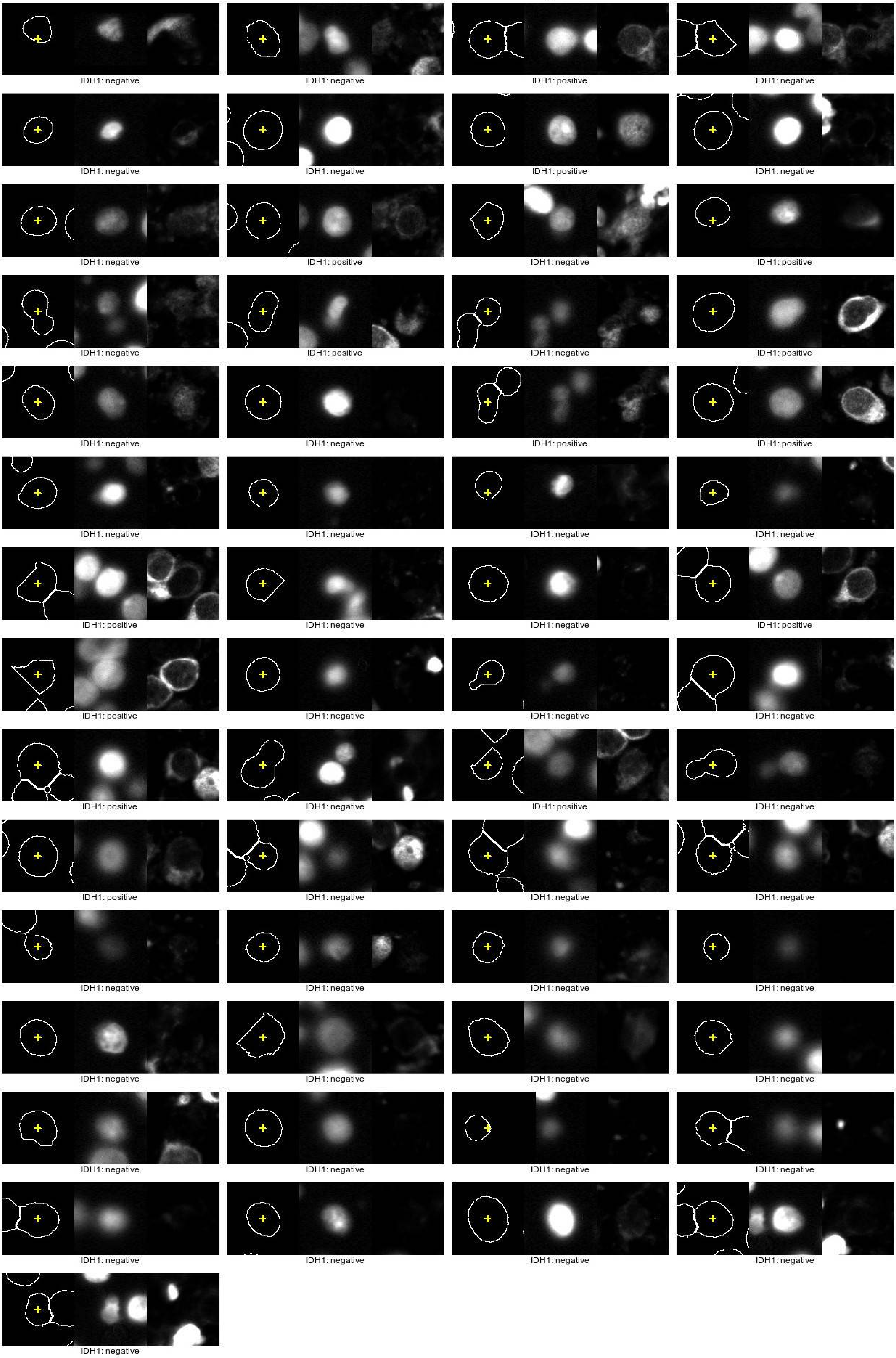

Case1_ROI_2 ZEB1 scoring

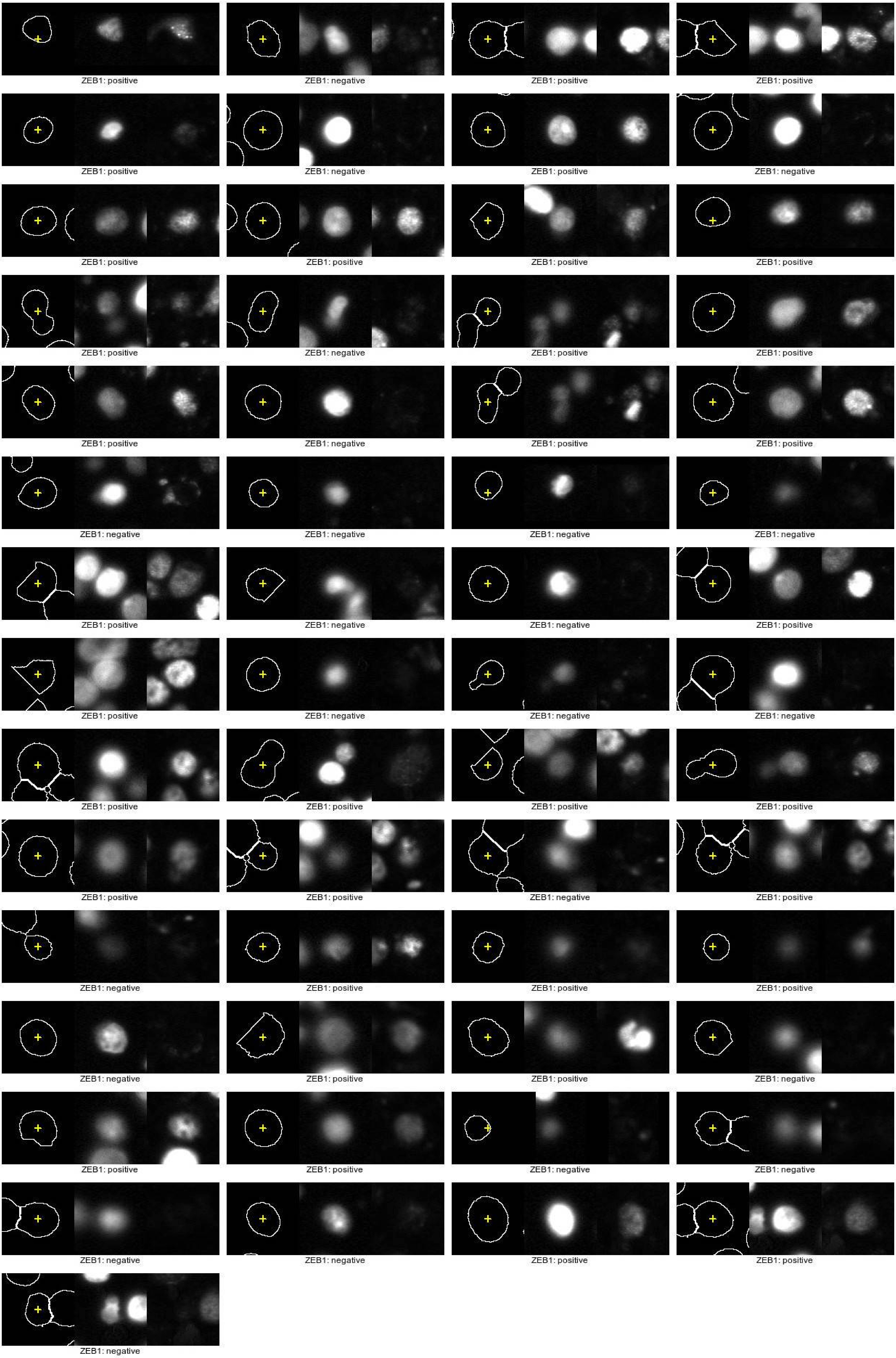

Case1_ROI_3 overview

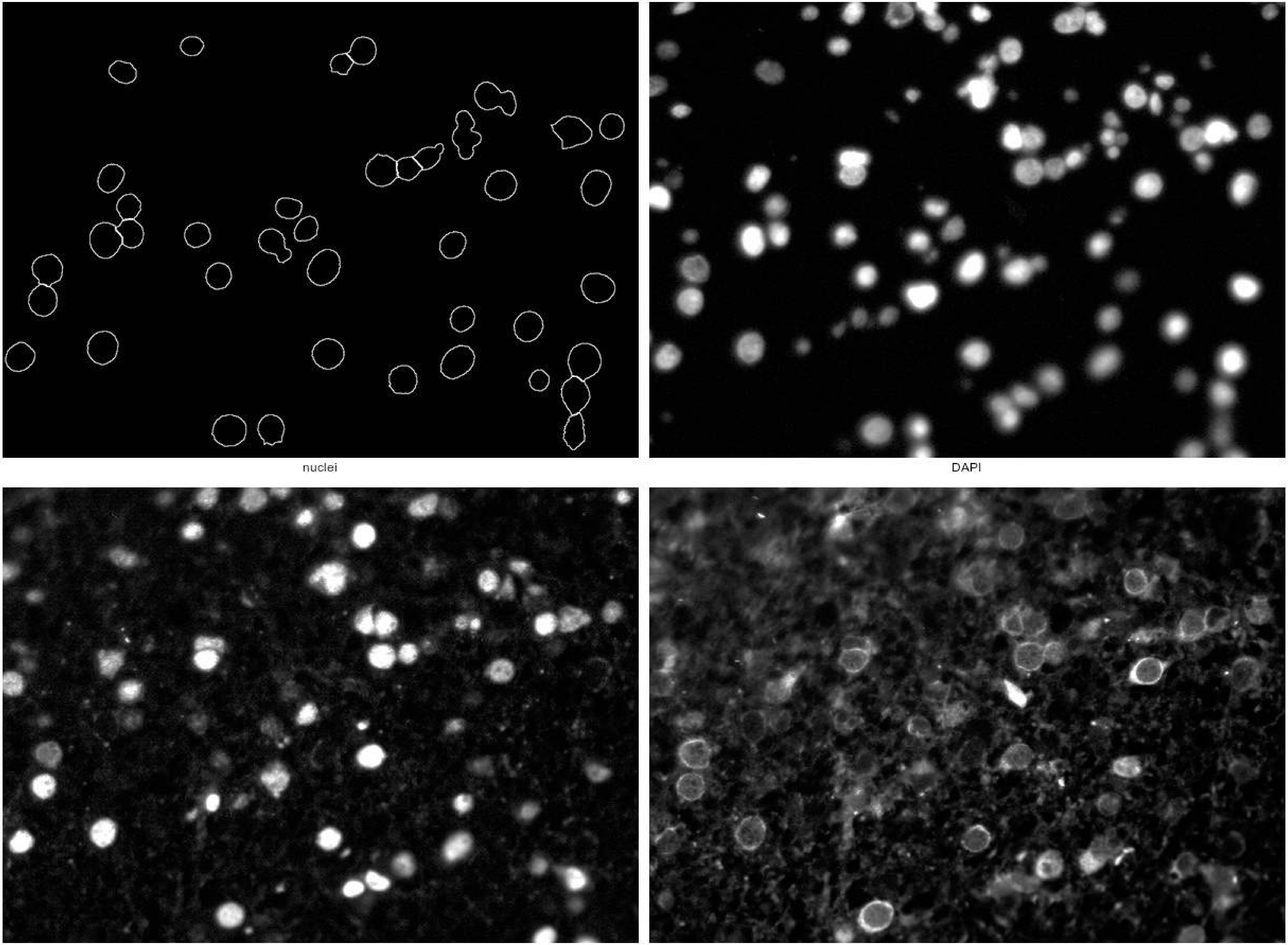

nuclei DAPI

ZEB1 IDH1 R132H

Case1_ROI_3 IDH1 scoring

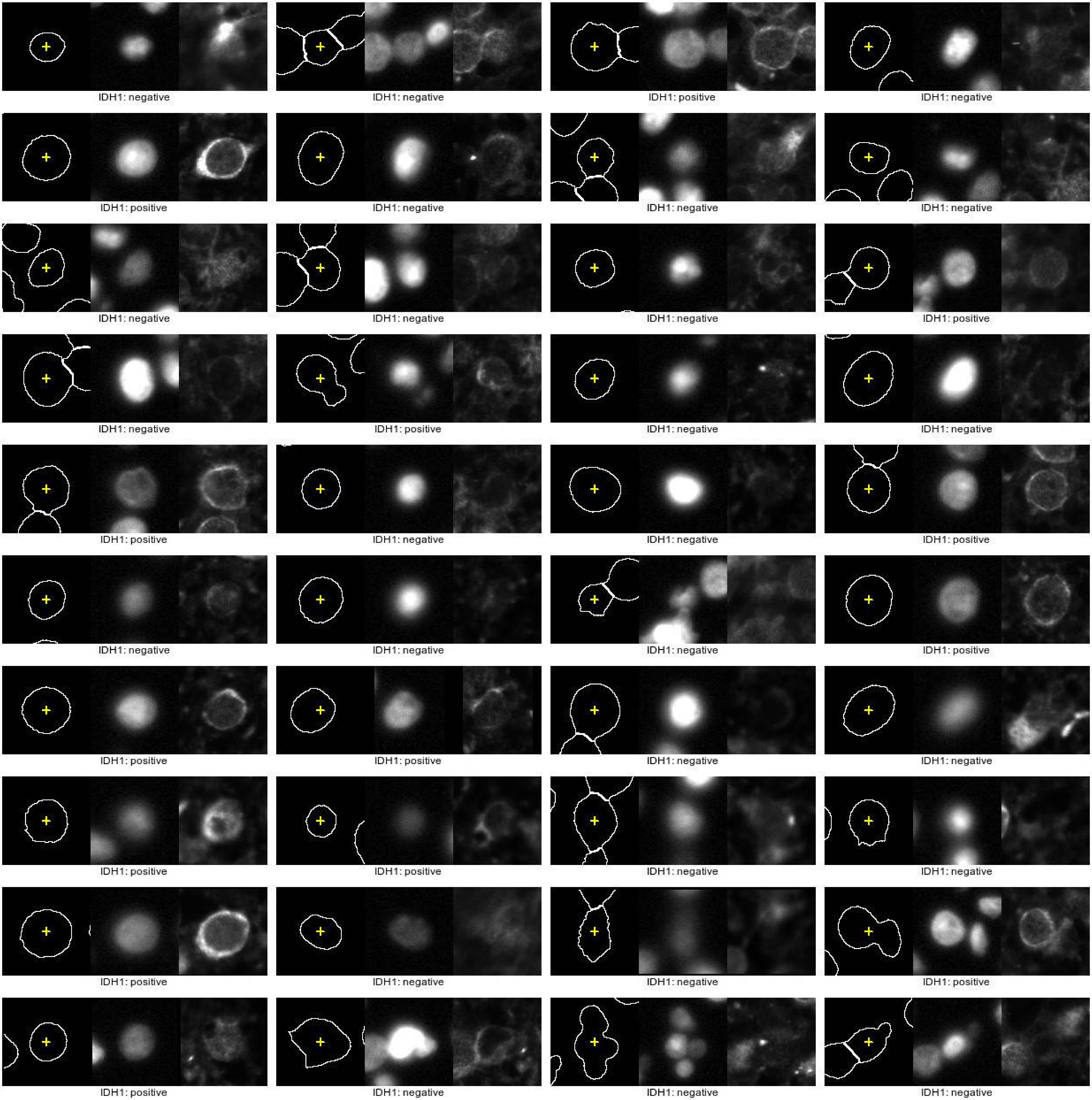

Case1_ROI_3 ZEB1 scoring

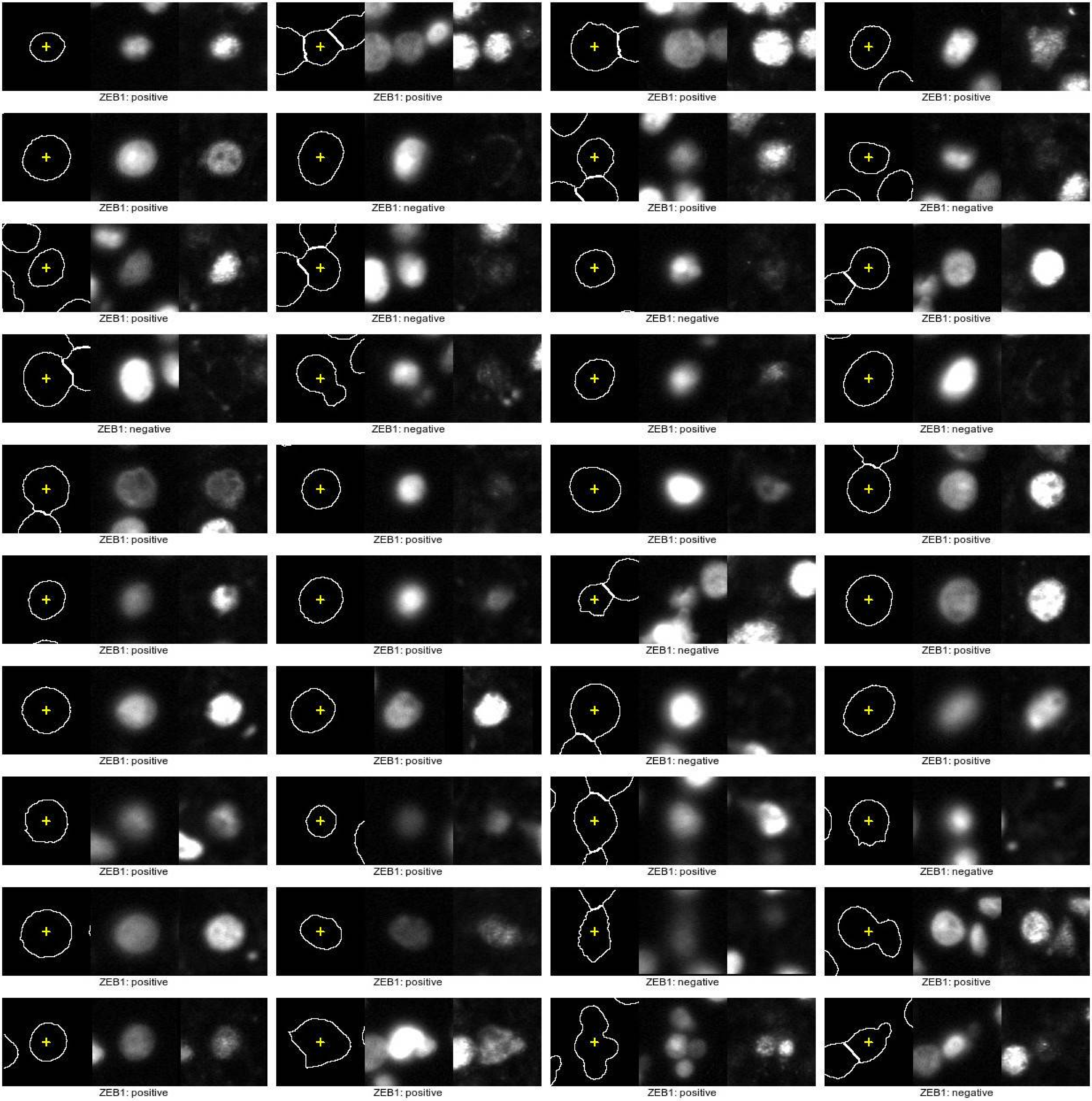

Case2_ROI_1 overview

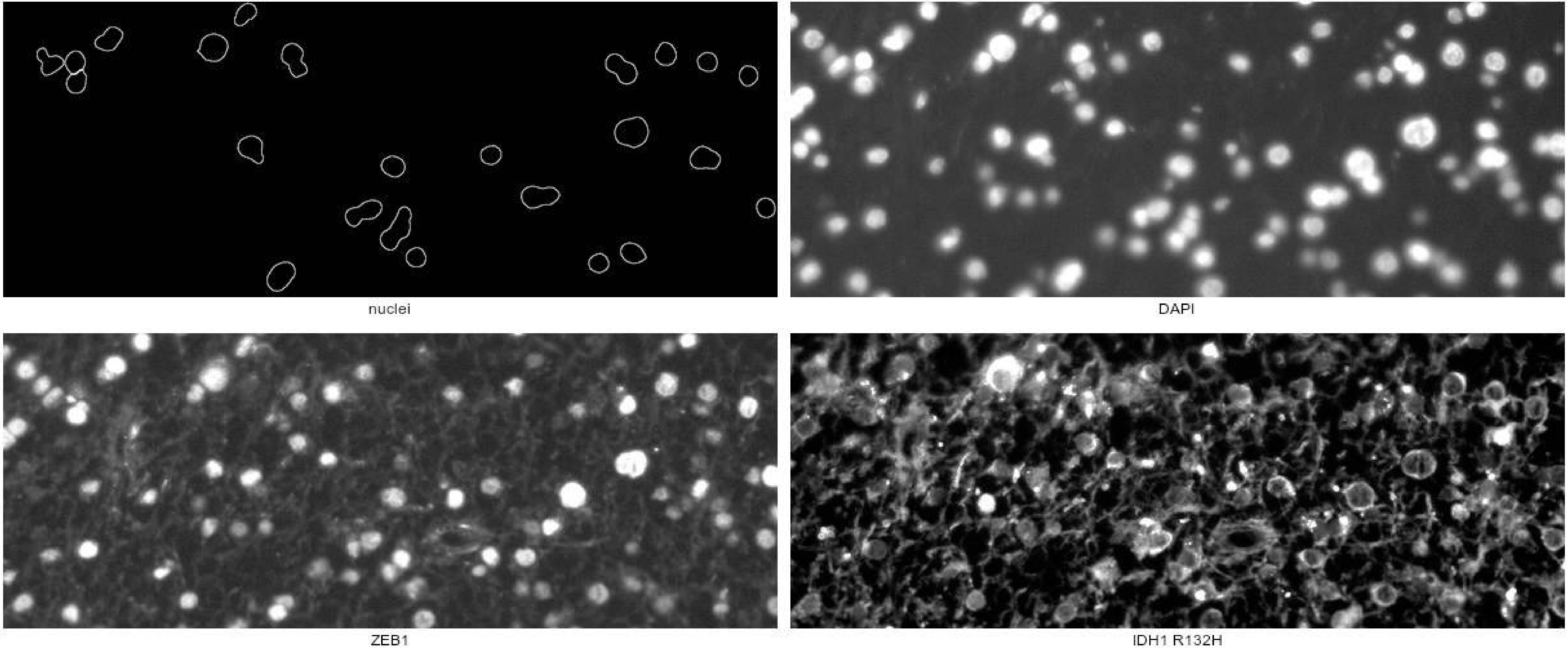

Case2_ROI_1 IDH1 scoring

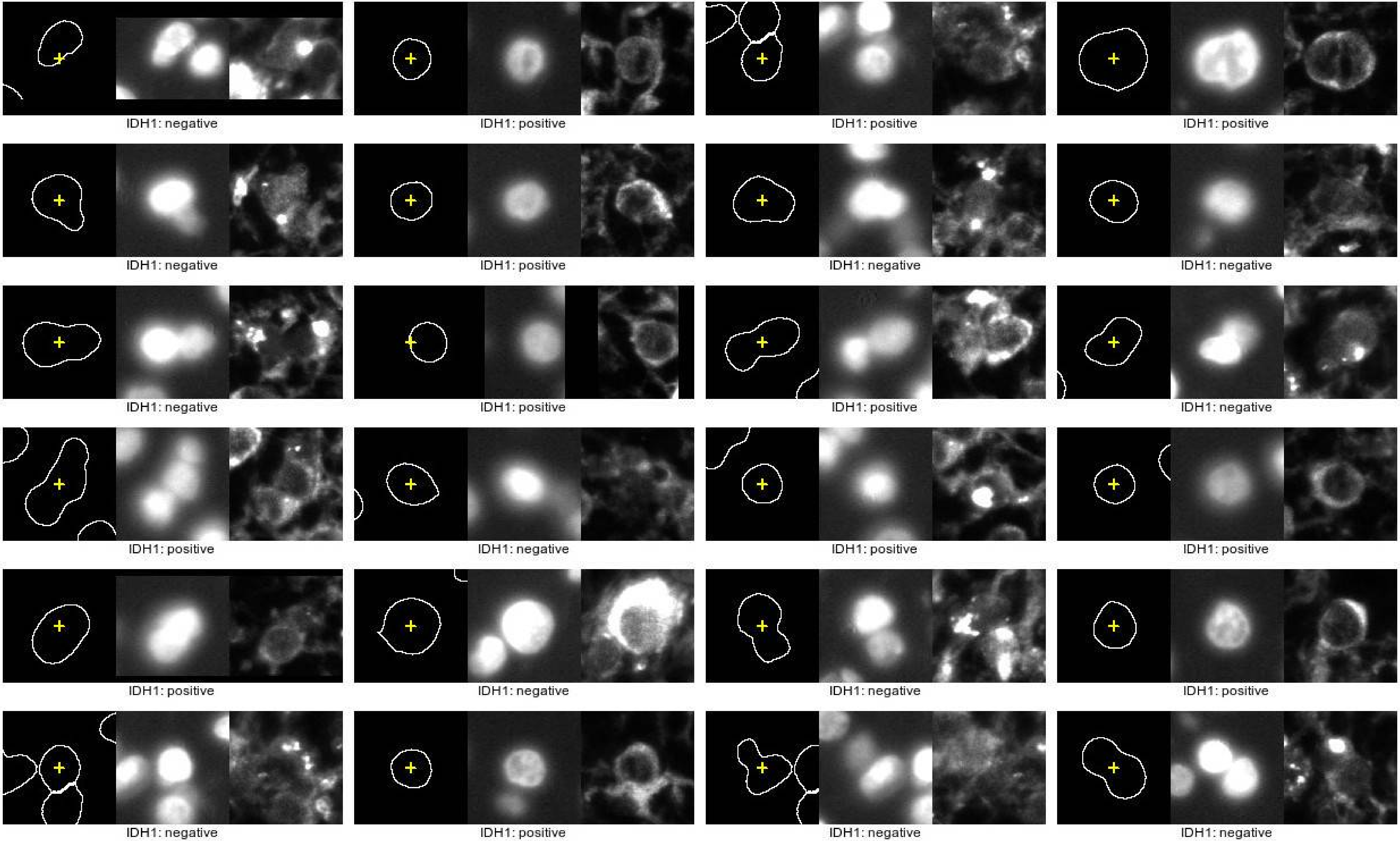

Case2_ROI_1 ZEB1 scoring

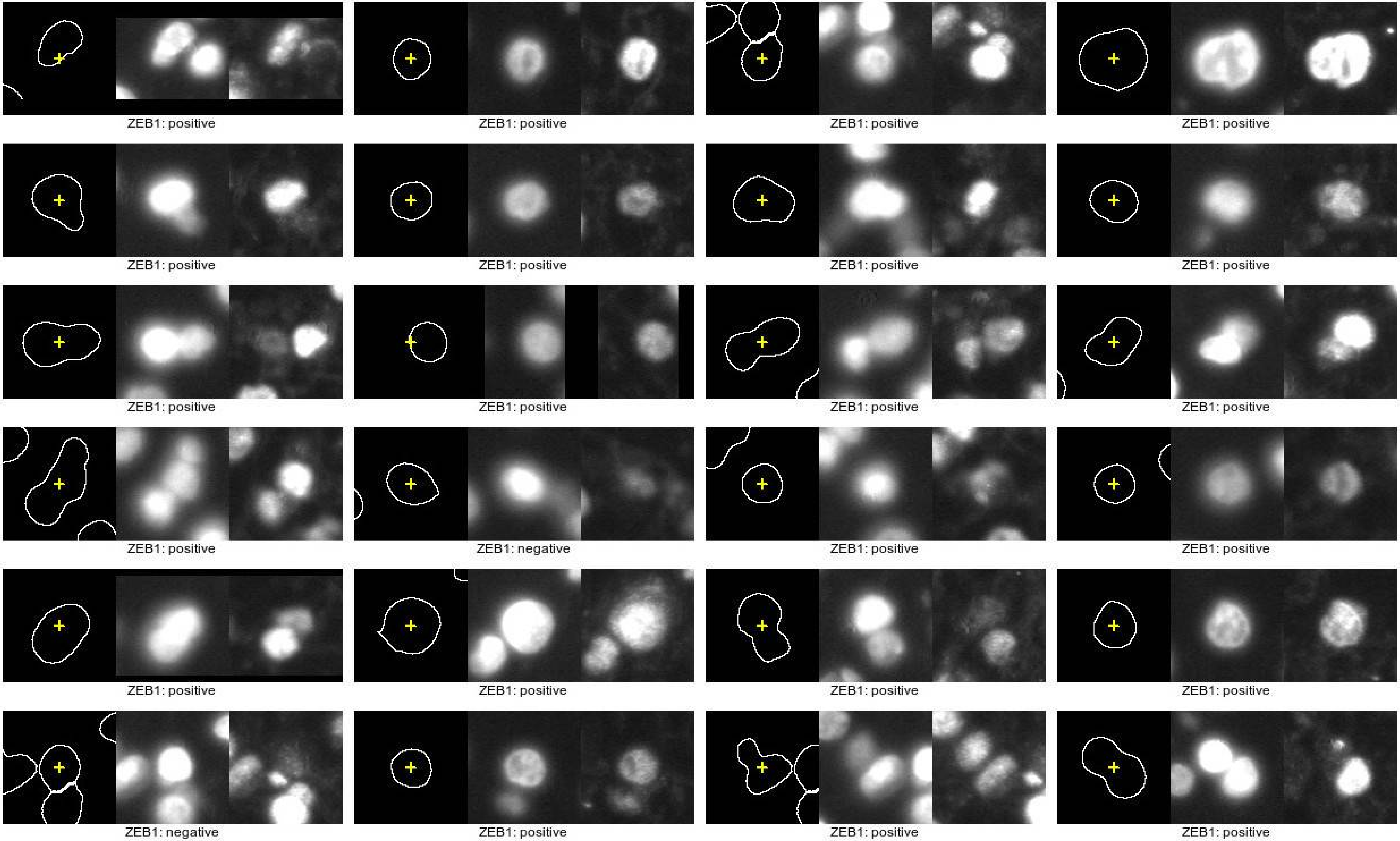

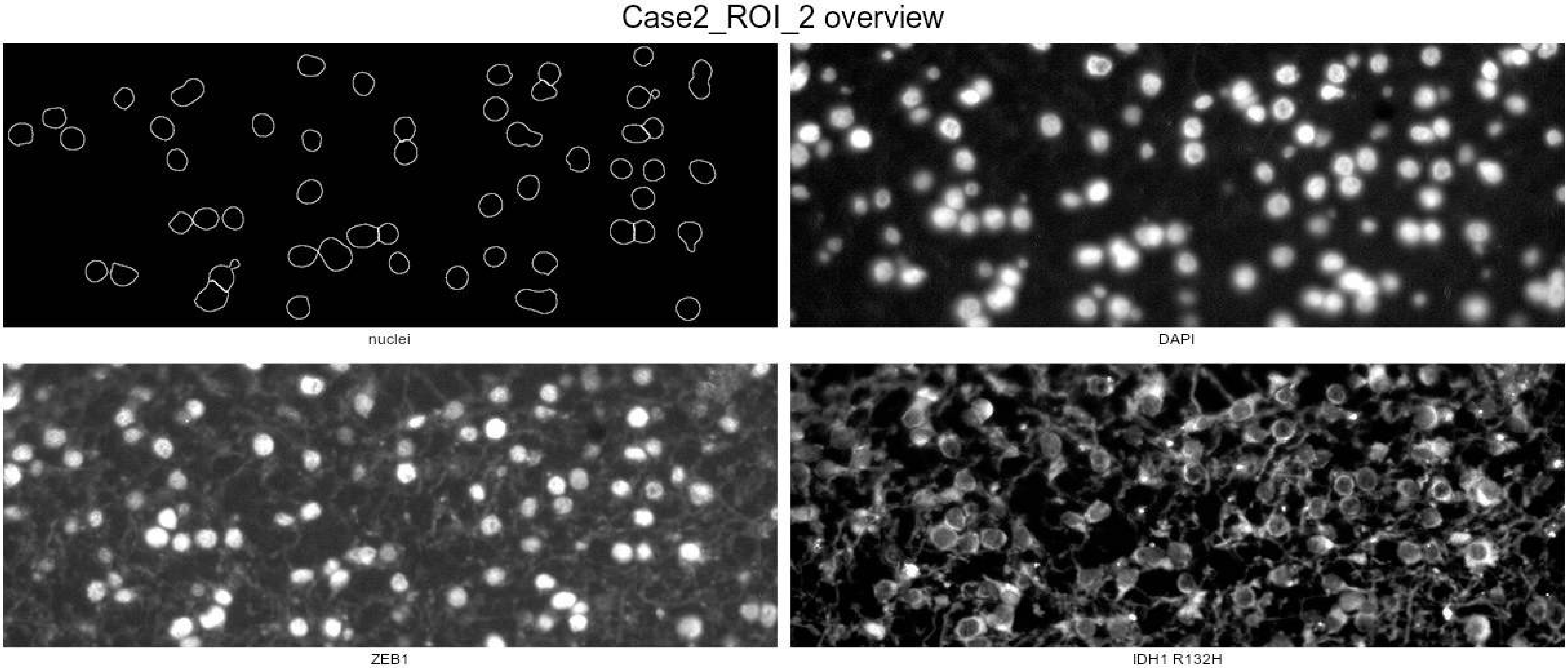
Case2_ROI_2 overview
nuclei
DAPI
ZEB1
IDH1 R132H

Case2_ROI_2 IDH1 scoring

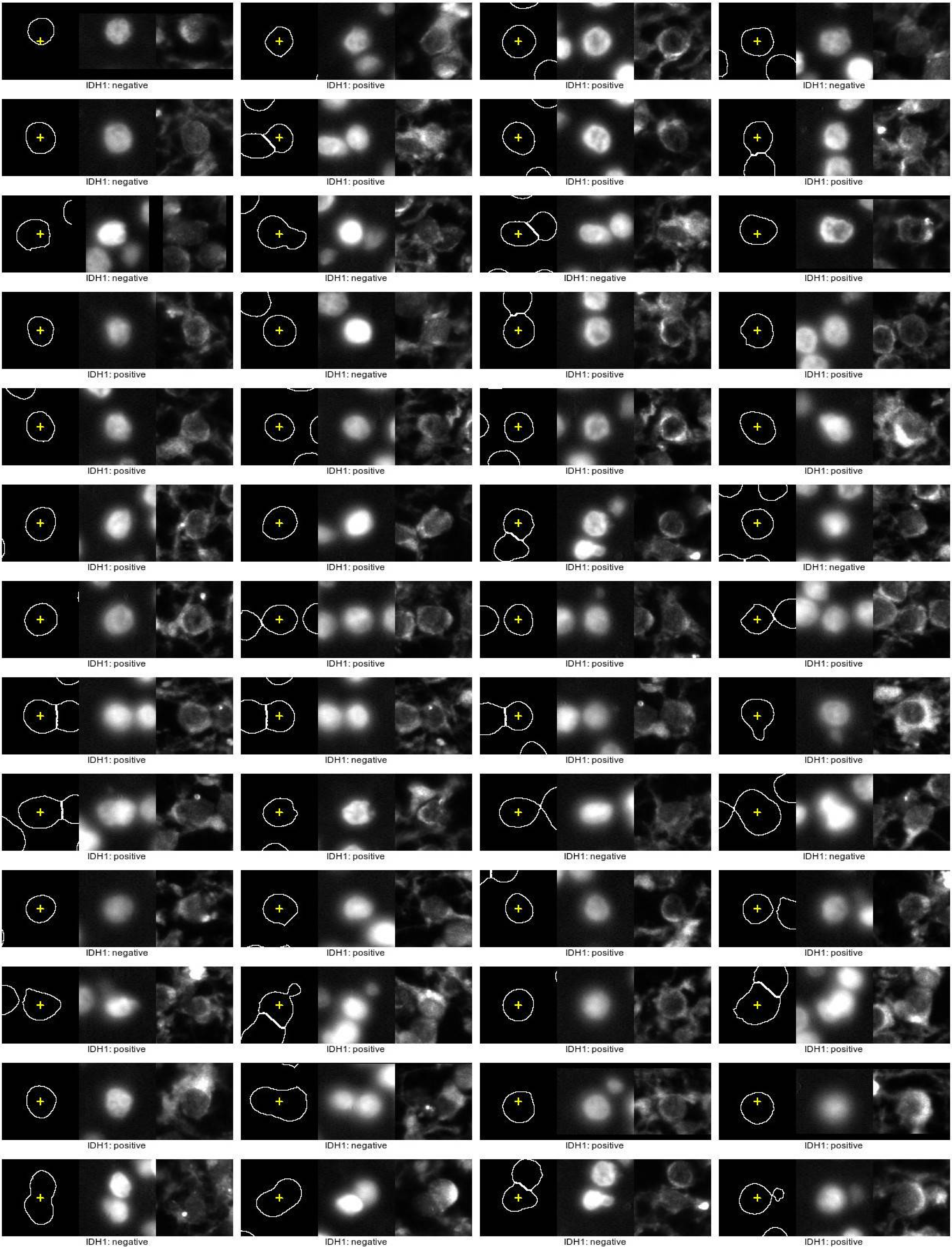

Case2_ROI_2 ZEB1 scoring

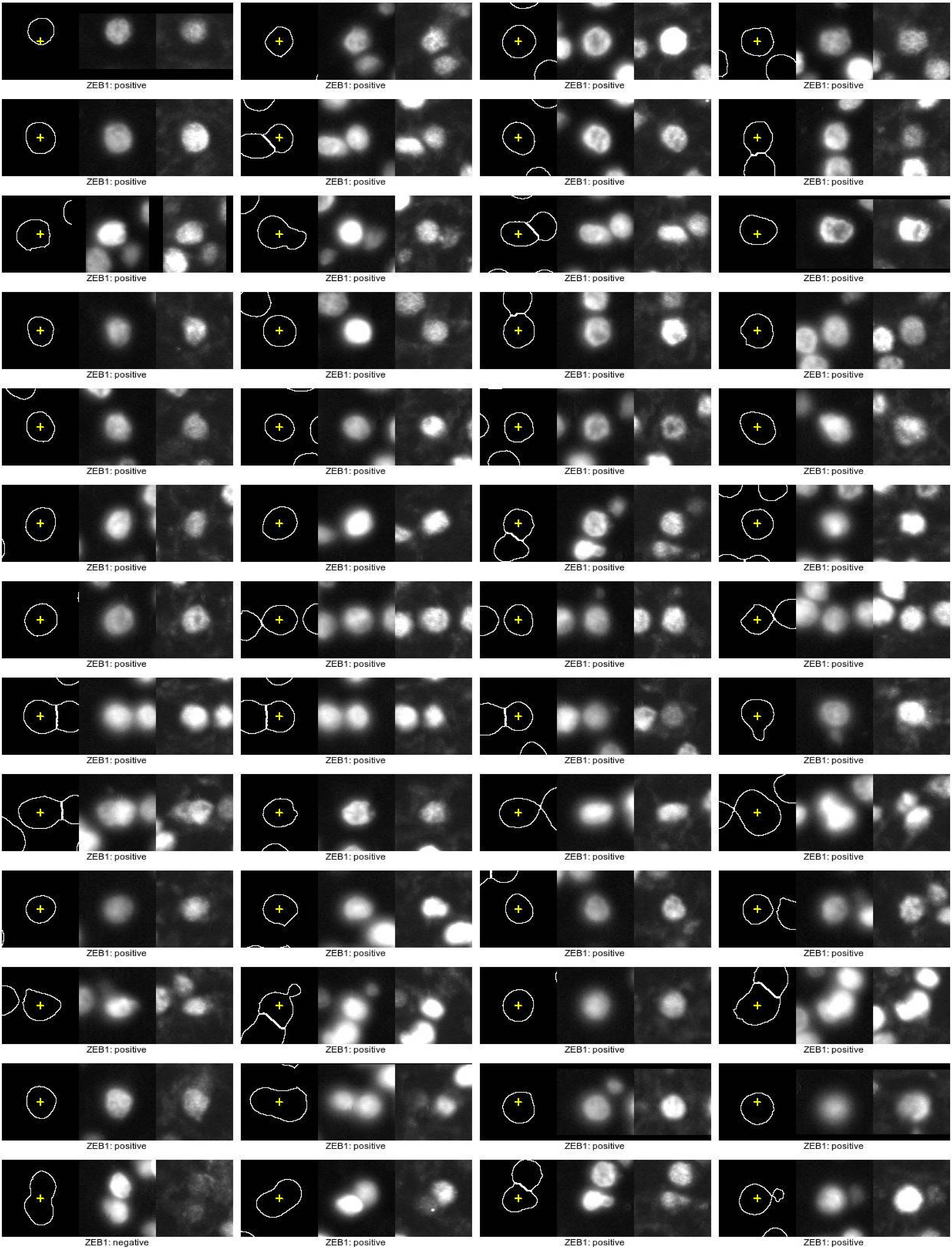

Case2_ROI_3 overview

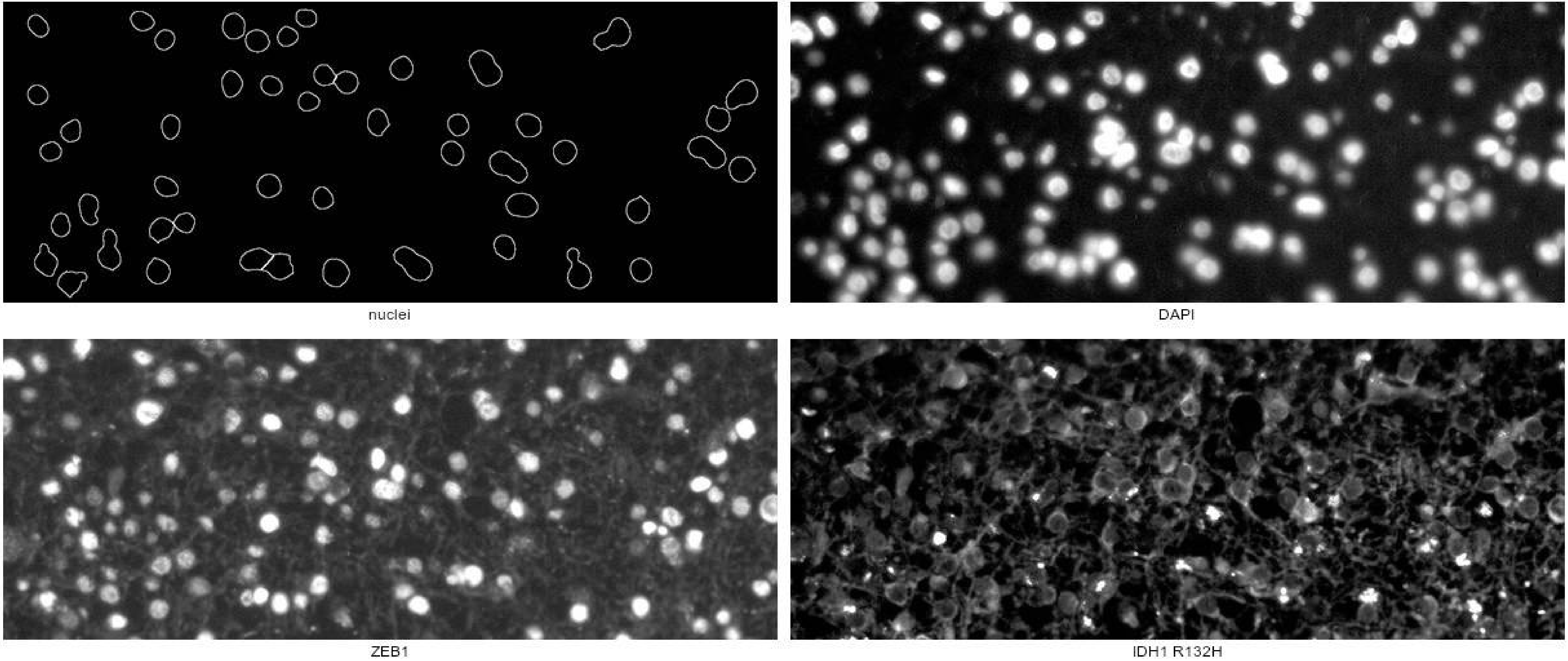

Case2_ROI_3 IDH1 scoring

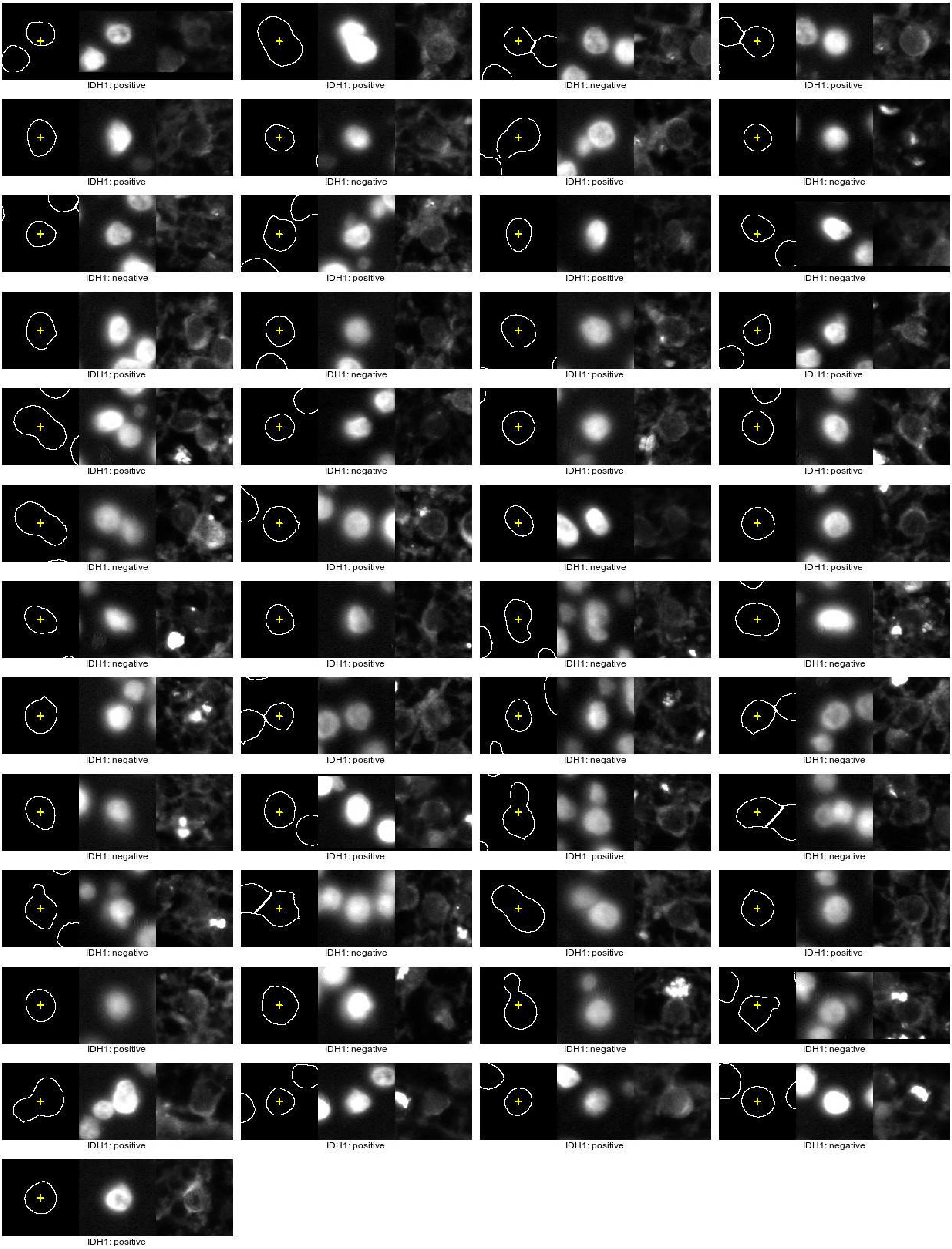

Case2_ROI_3 ZEB1 scoring

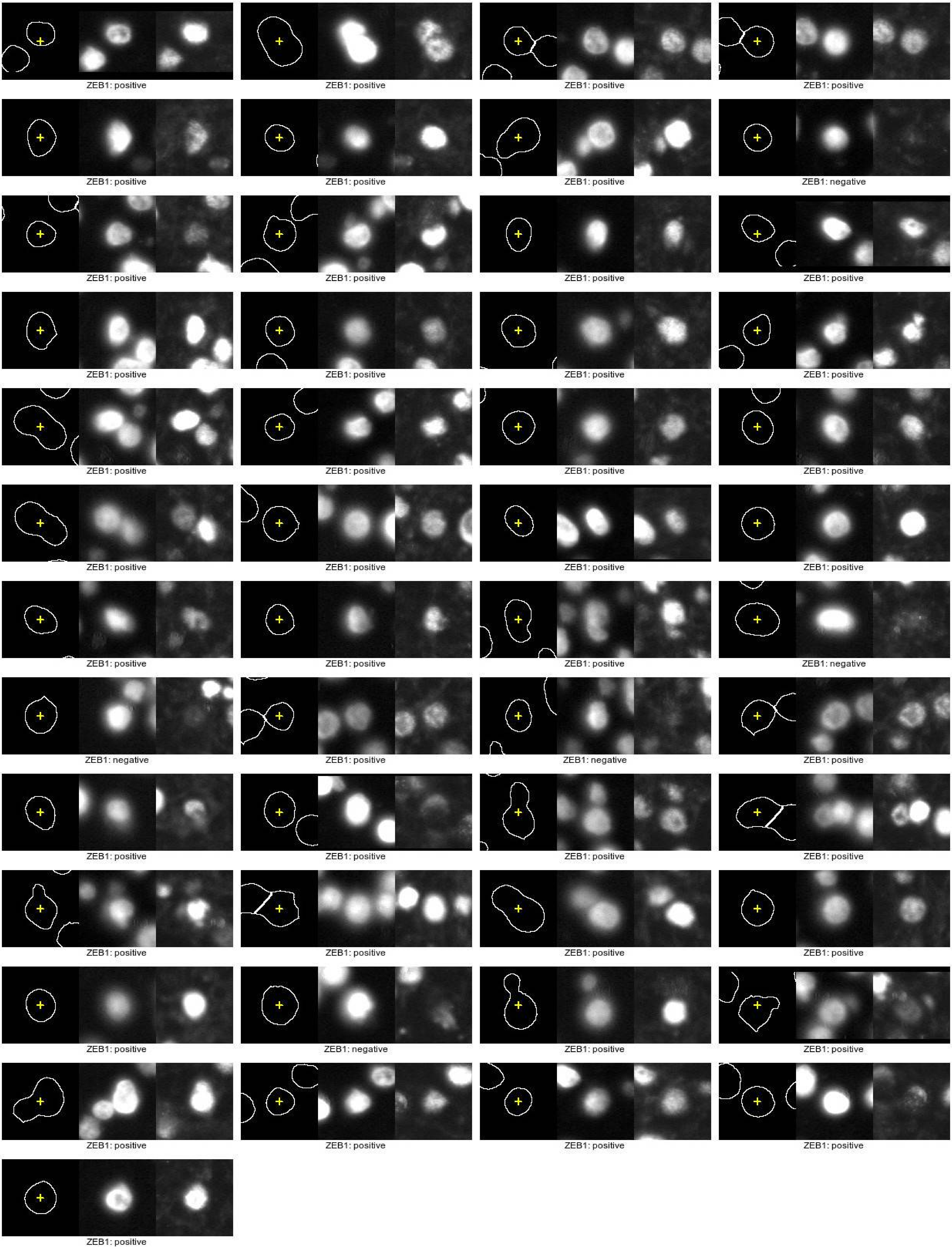

Case3_ROI_1 overview

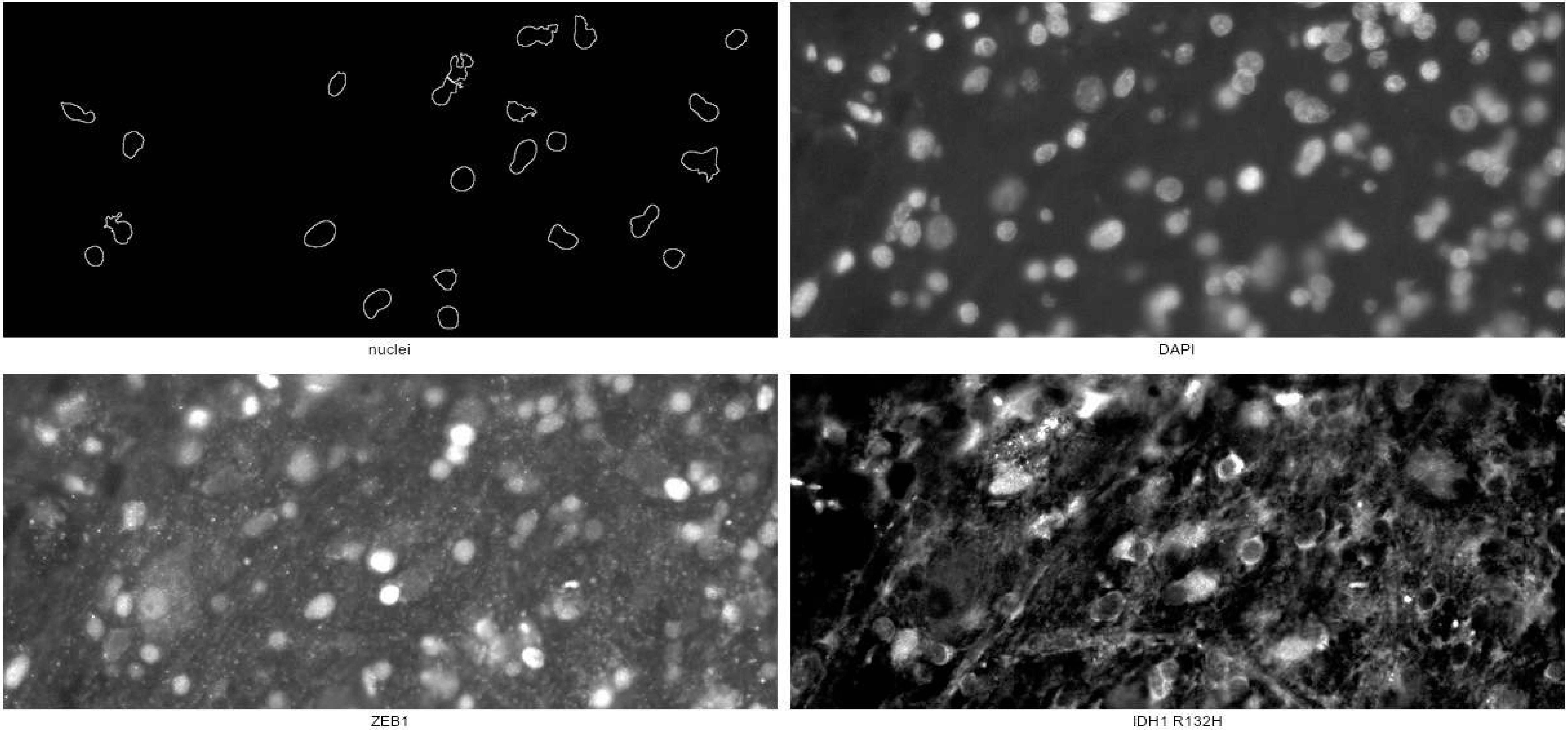

Case3_ROI_1 IDH1 scoring

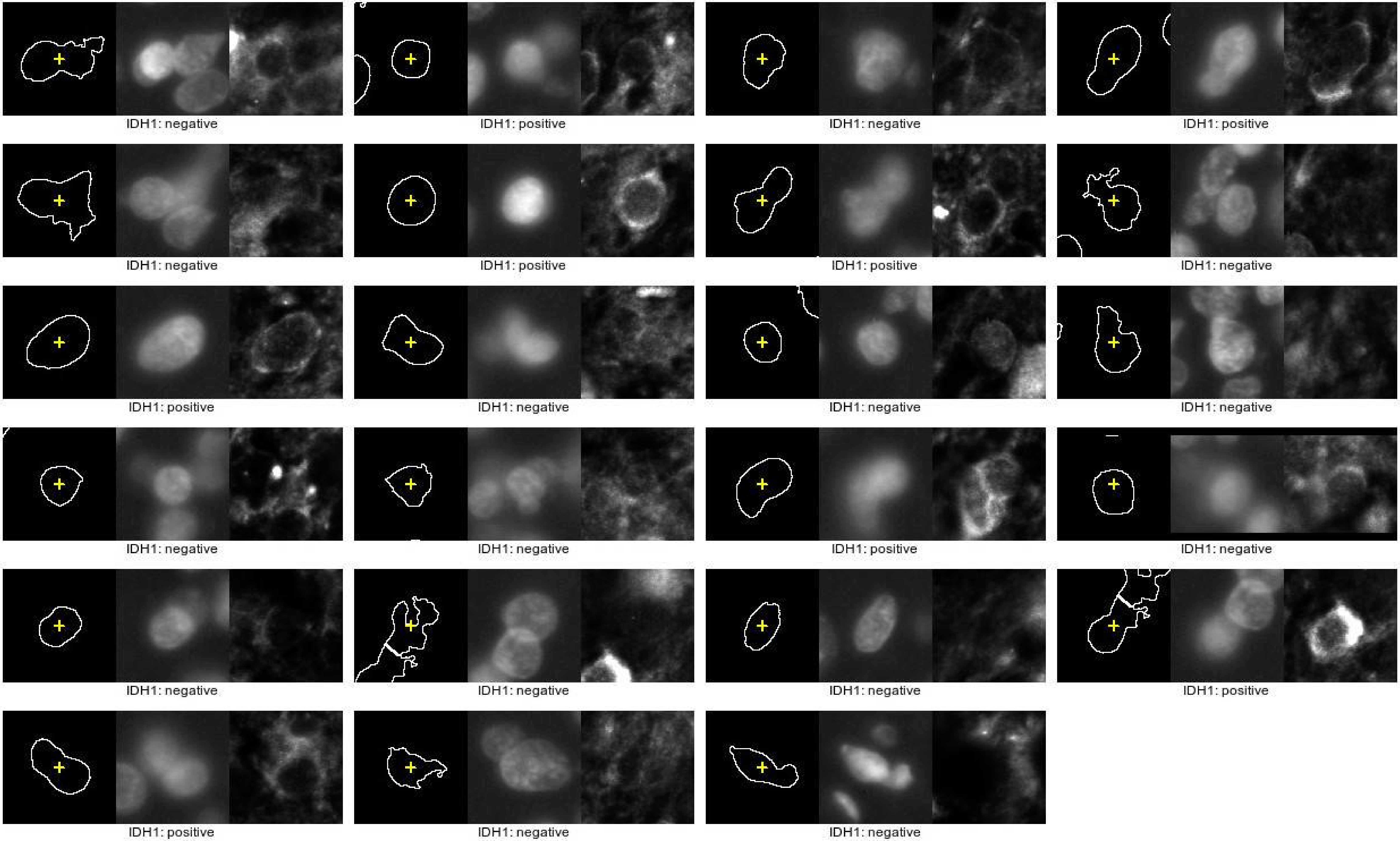

Case3_ROI_1 ZEB1 scoring

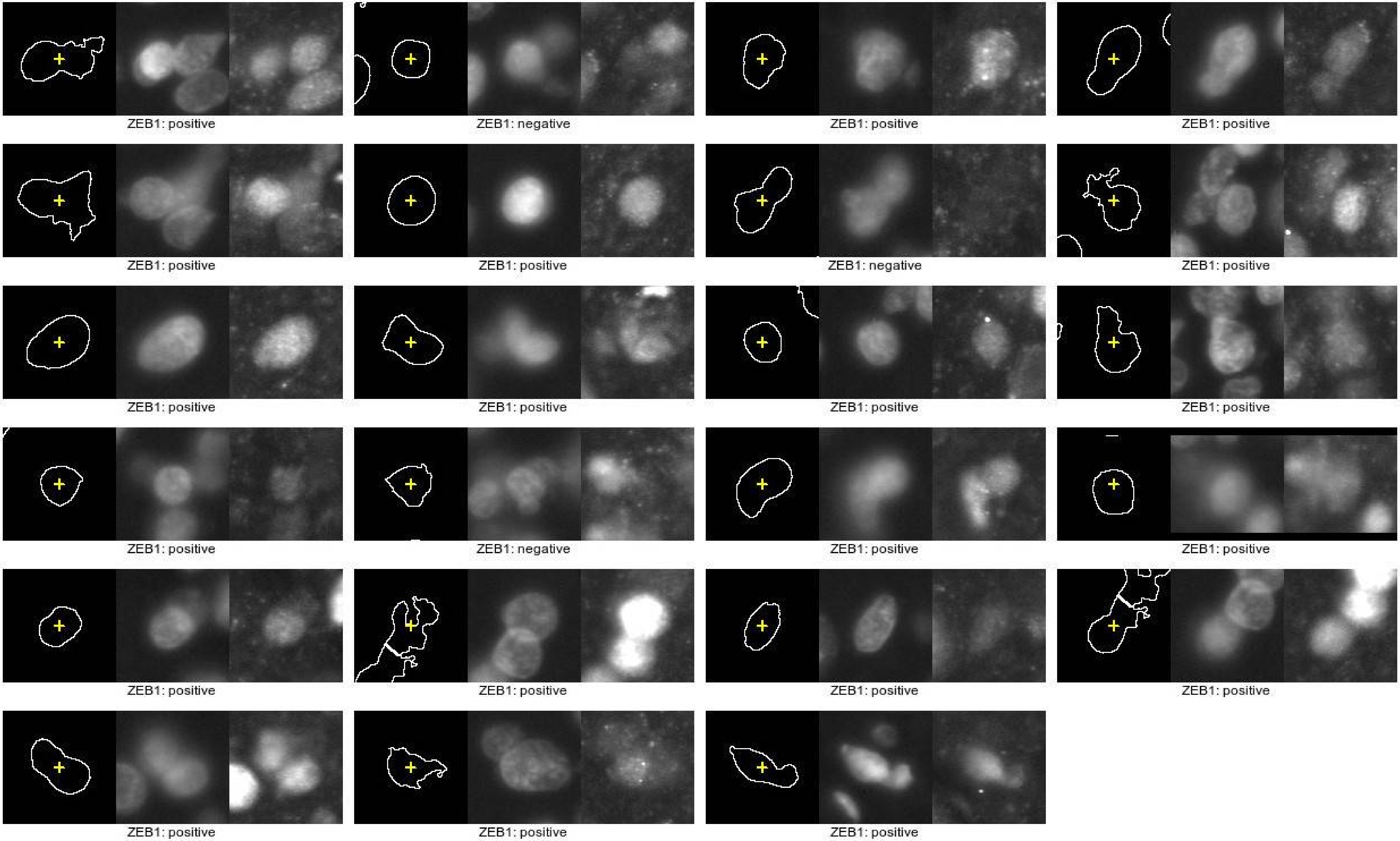

Case3_ROI_2 overview

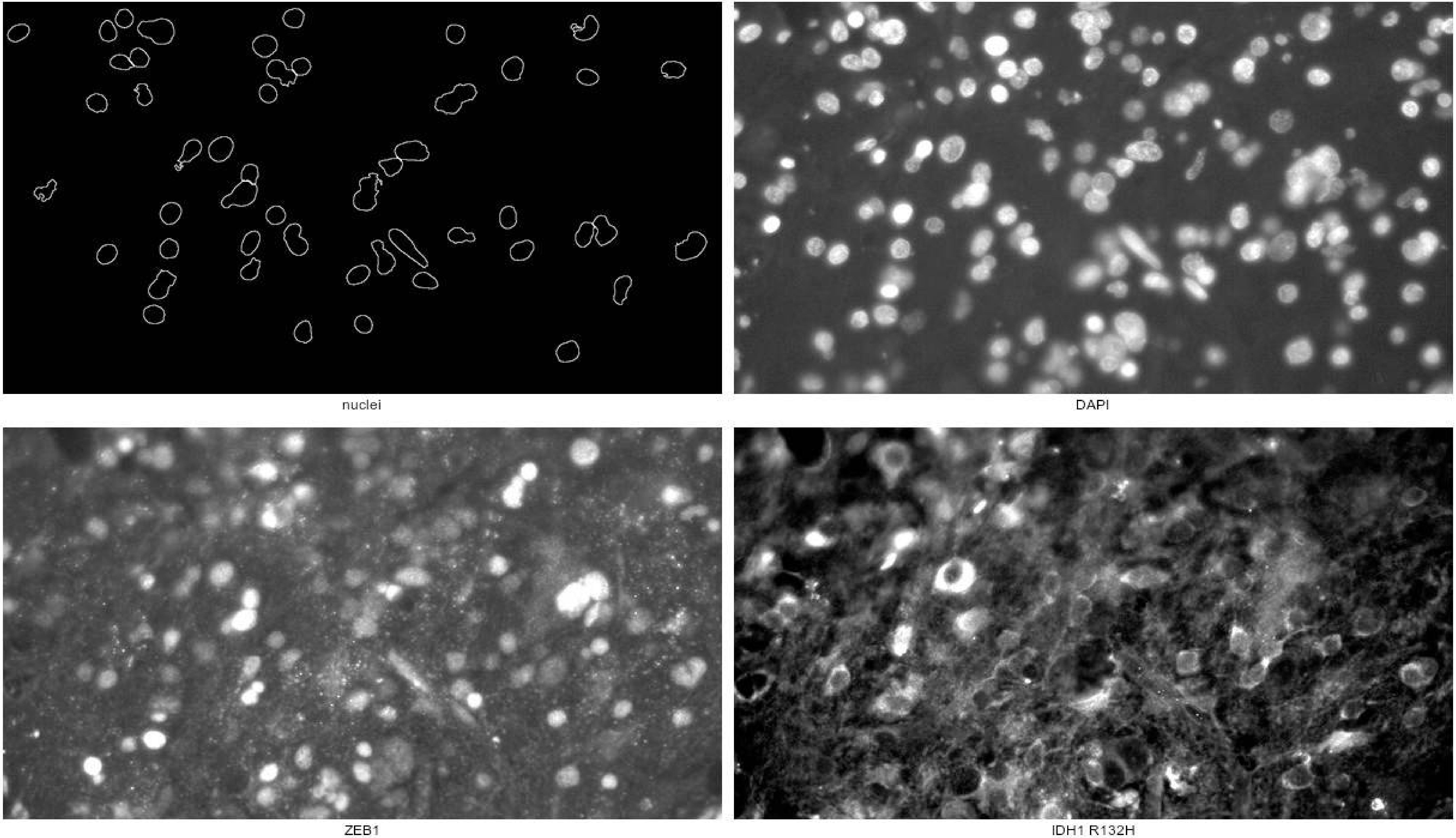

Case3_ROI_2 IDH1 scoring

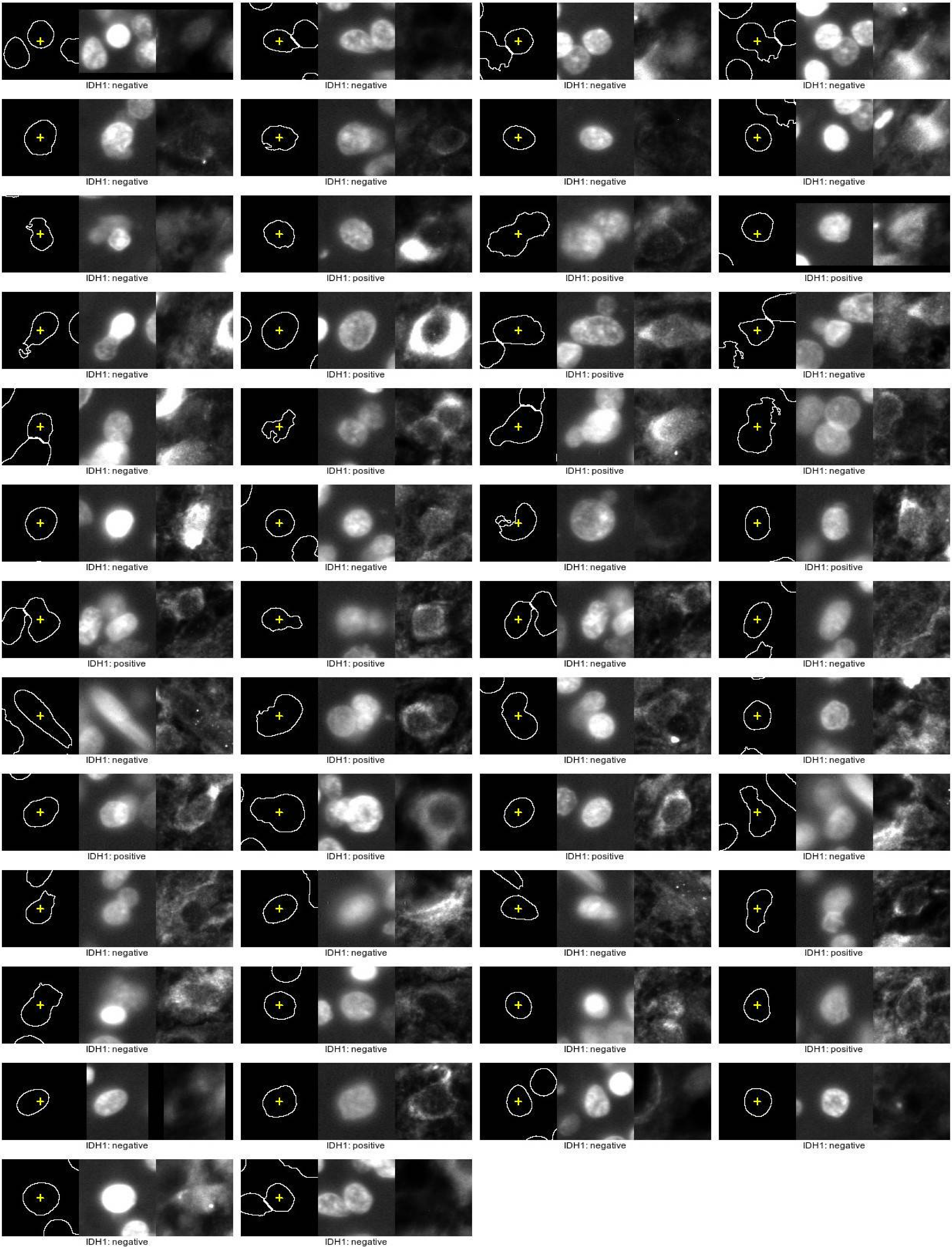

Case3_ROI_2 ZEB1 scoring

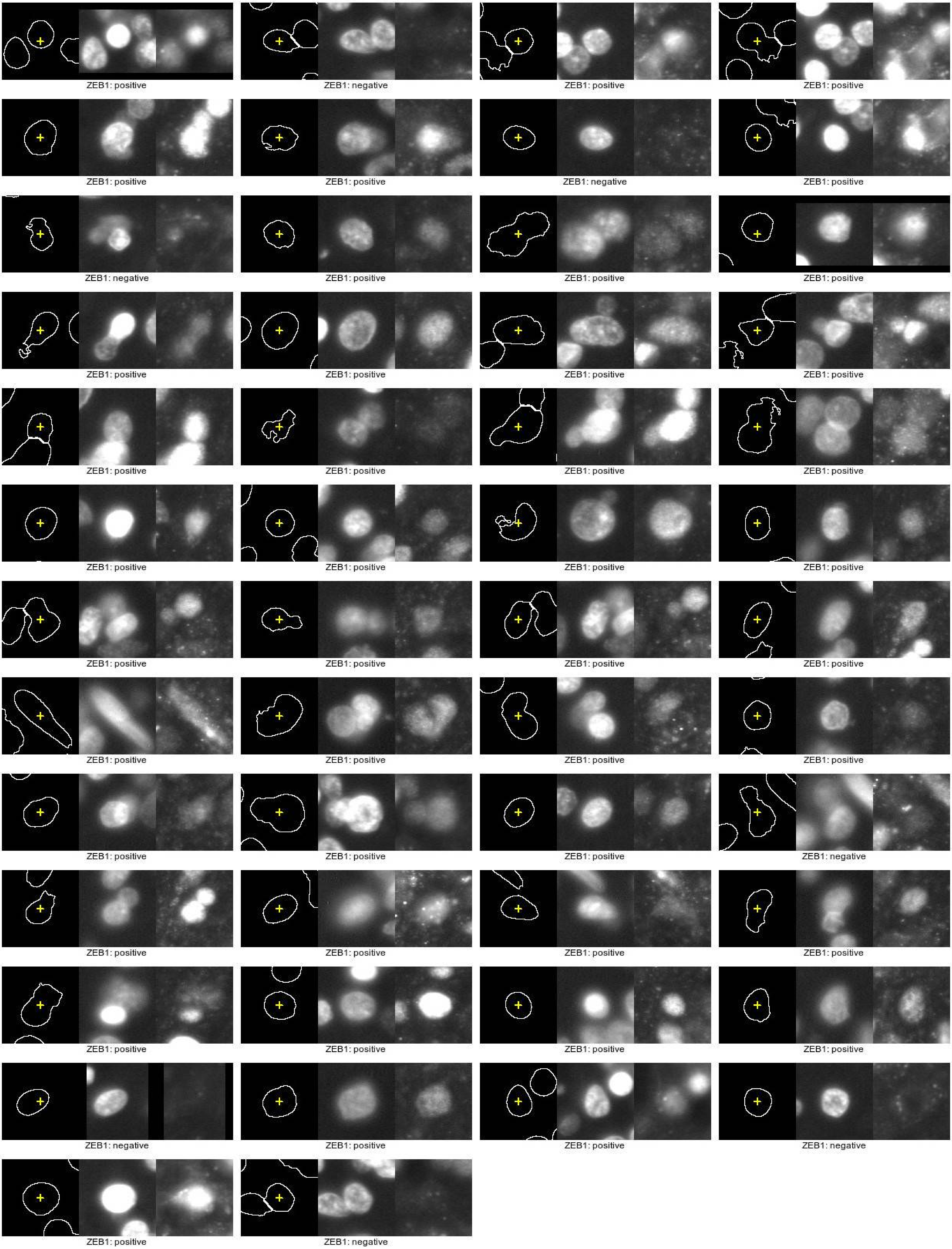

Case3_ROI_3 overview

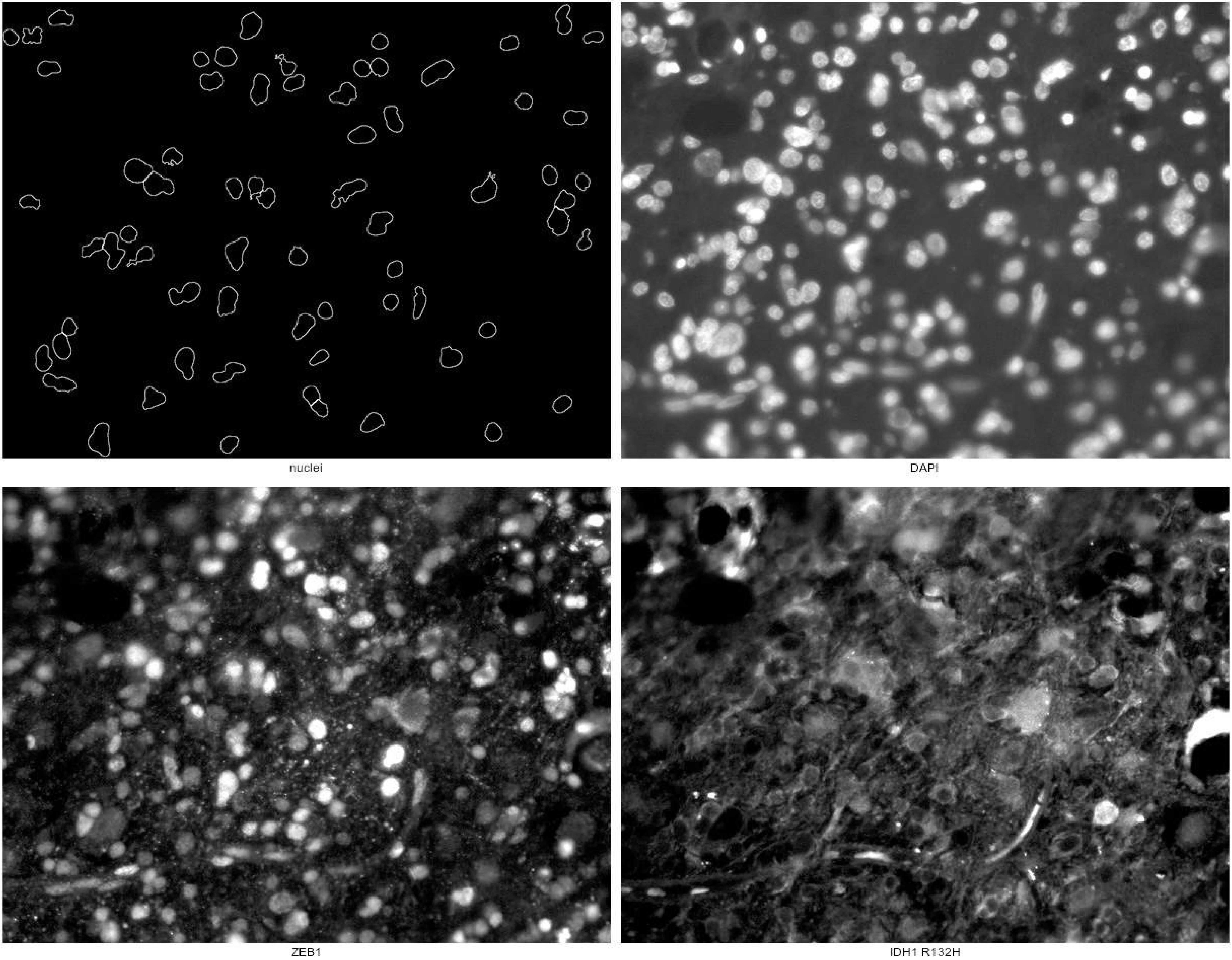

Case3_ROI_3 IDH1 scoring

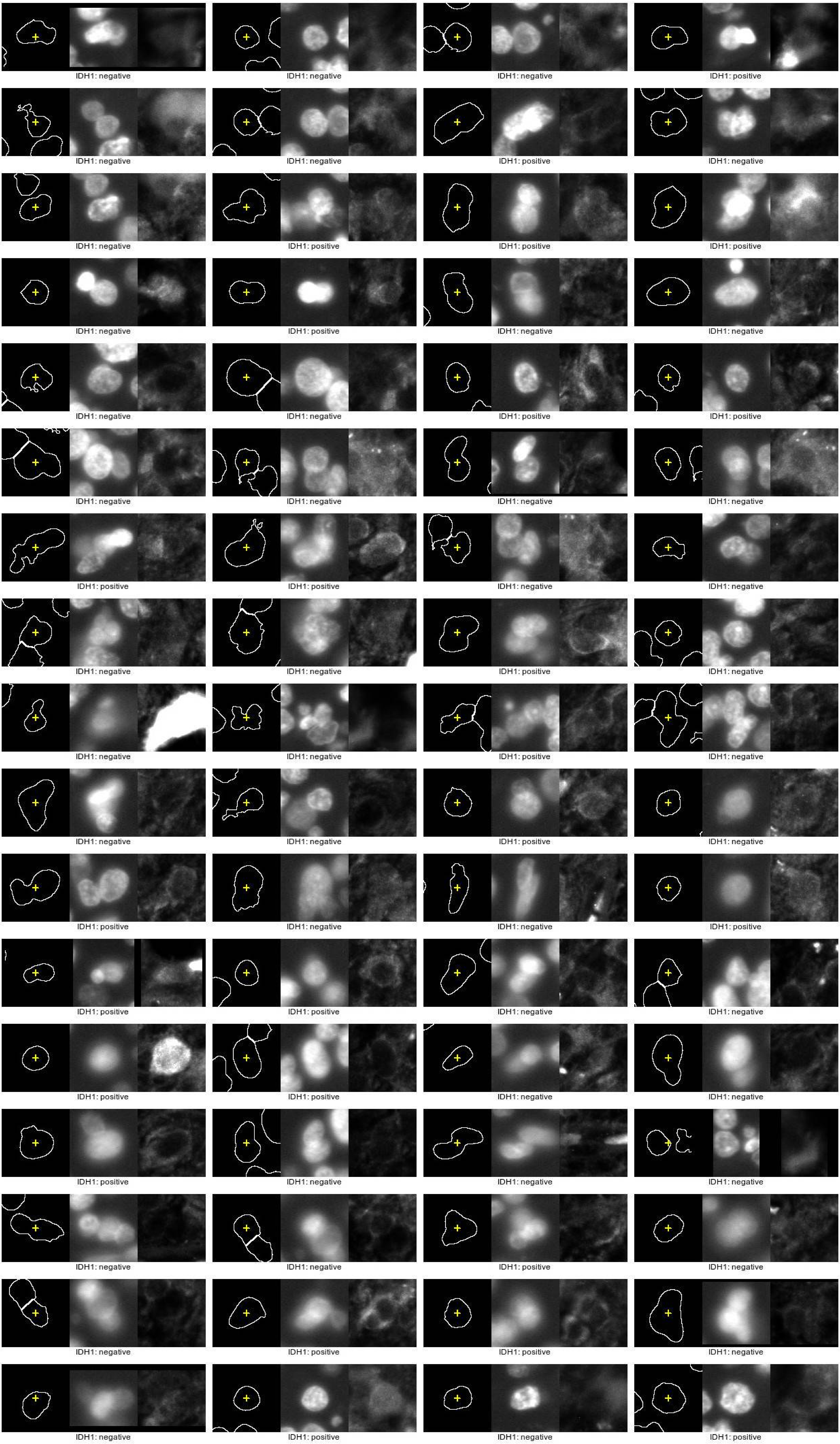

Case3_ROI_3 ZEB1 scoring

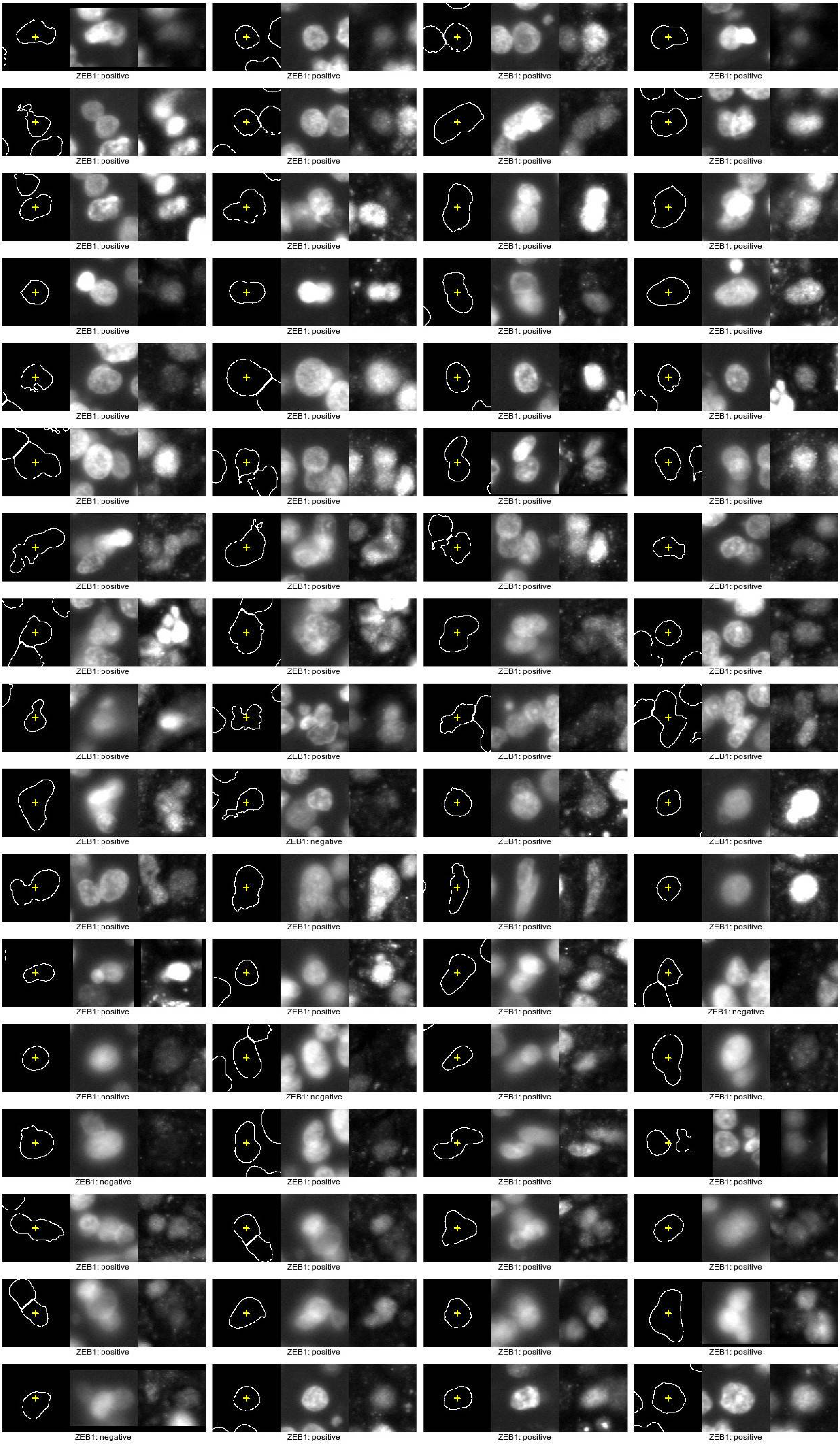

Case4_ROI_1 overview

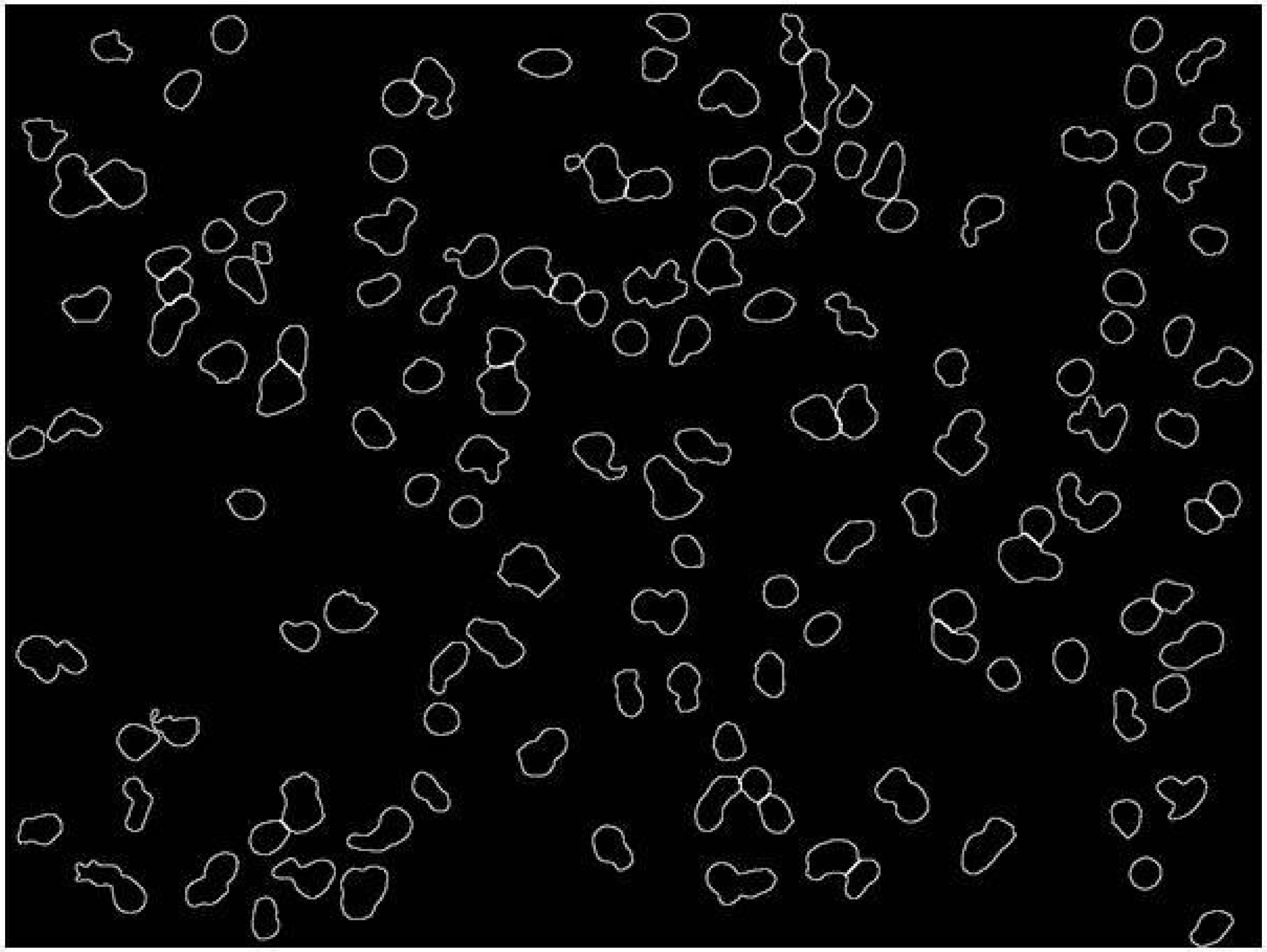

nuclei

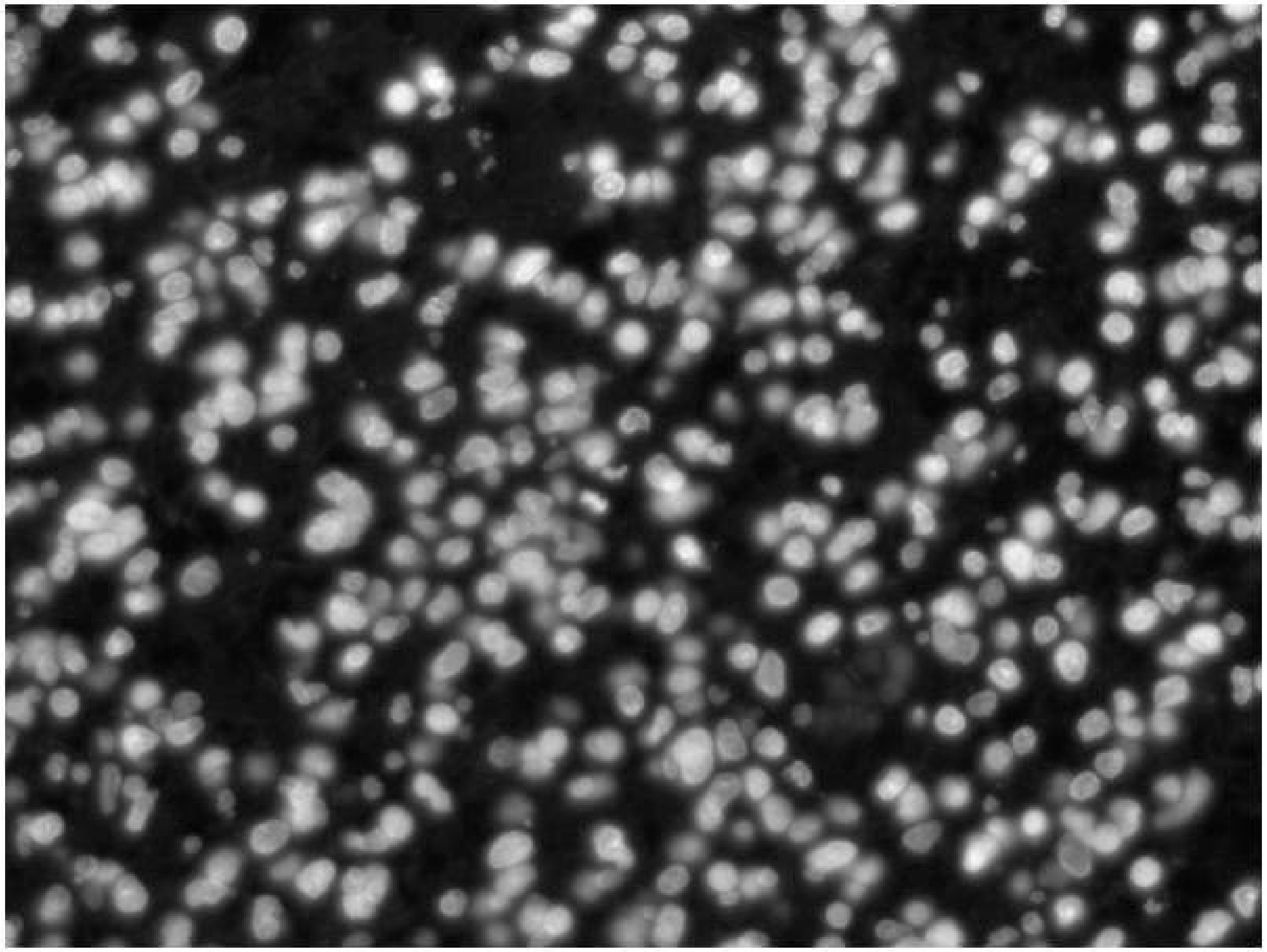

DAPI

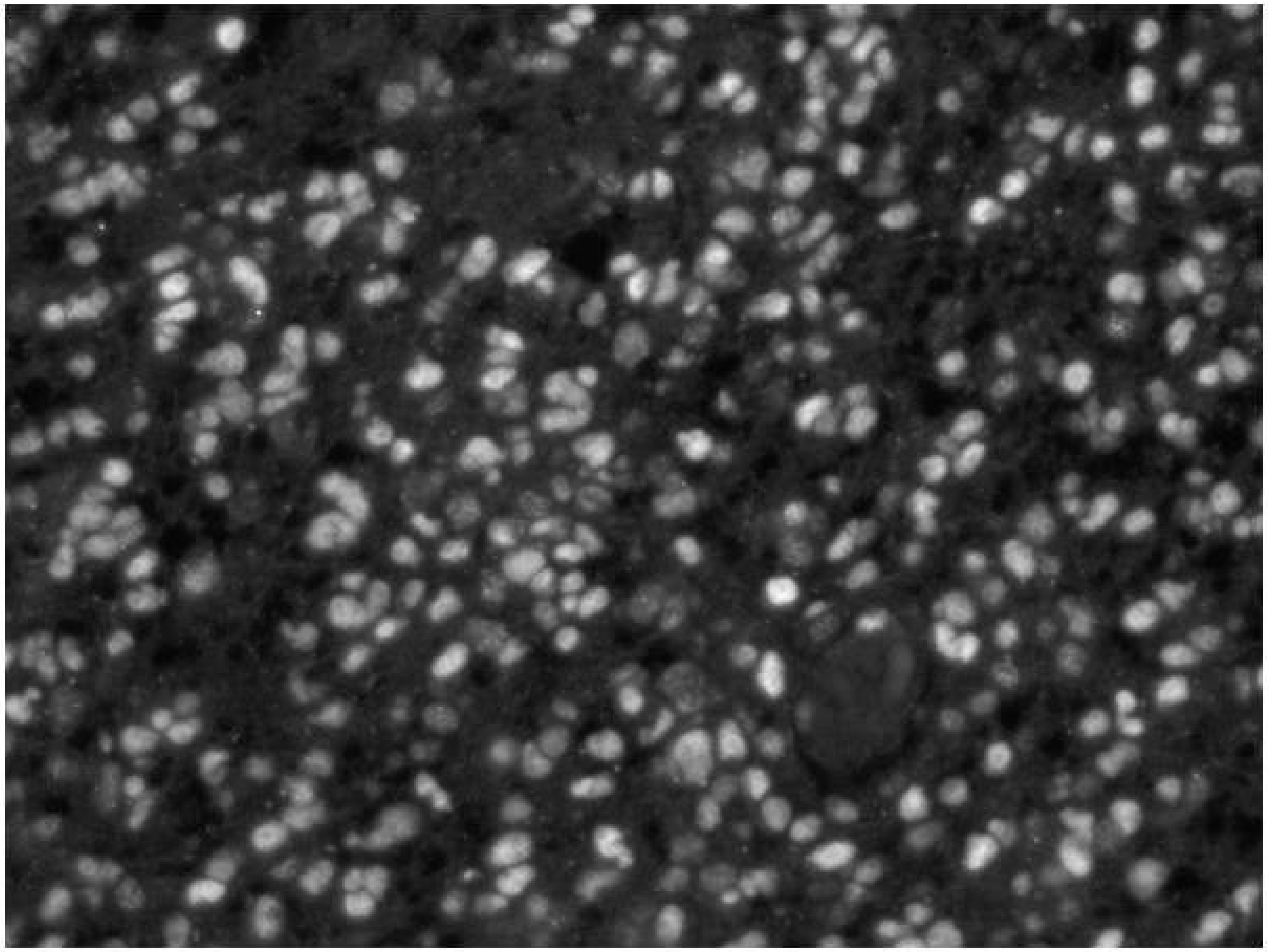

ZEB1

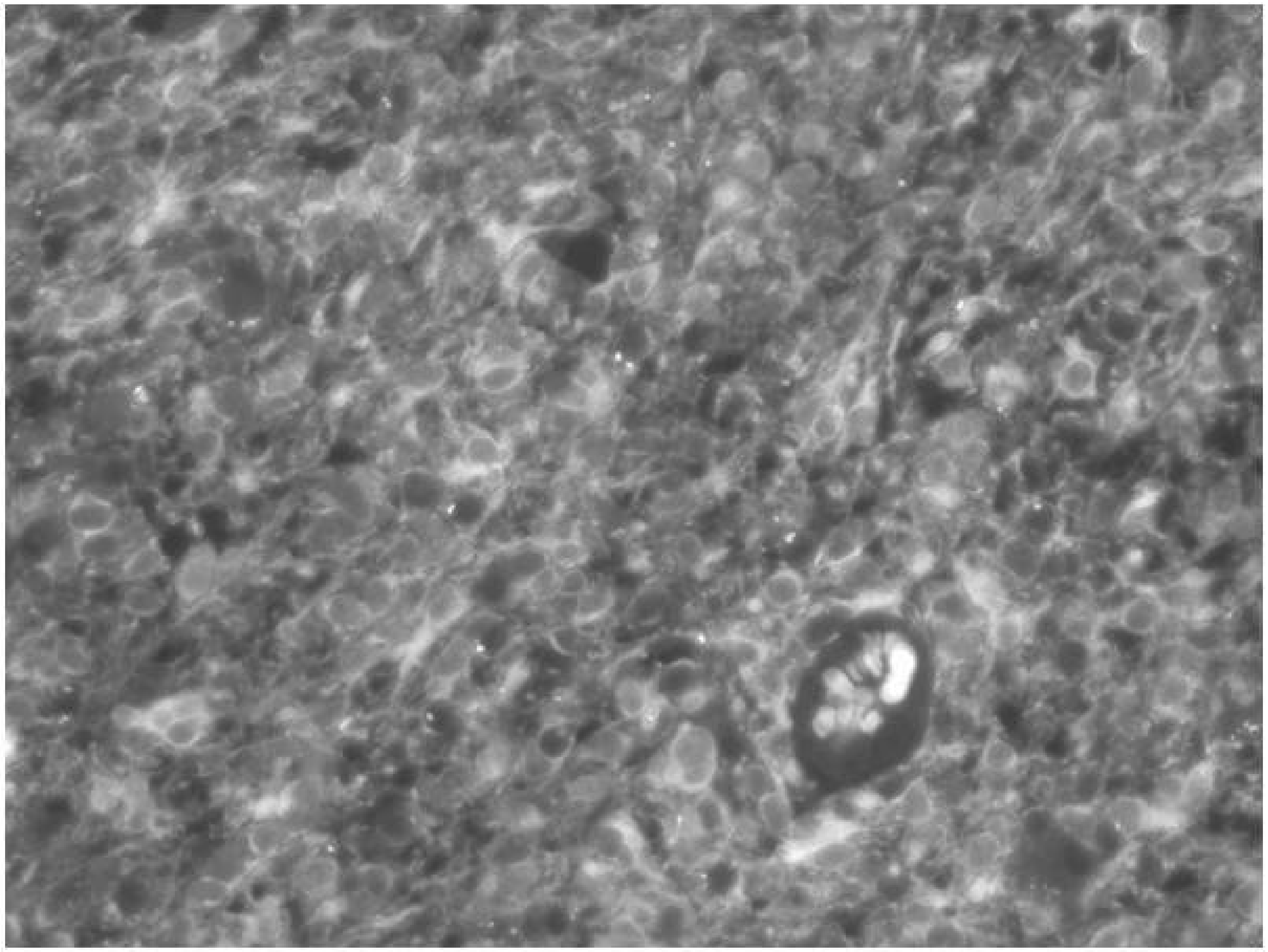

IDH1 R132H

Case4_ROI_1 IDH1 scoring

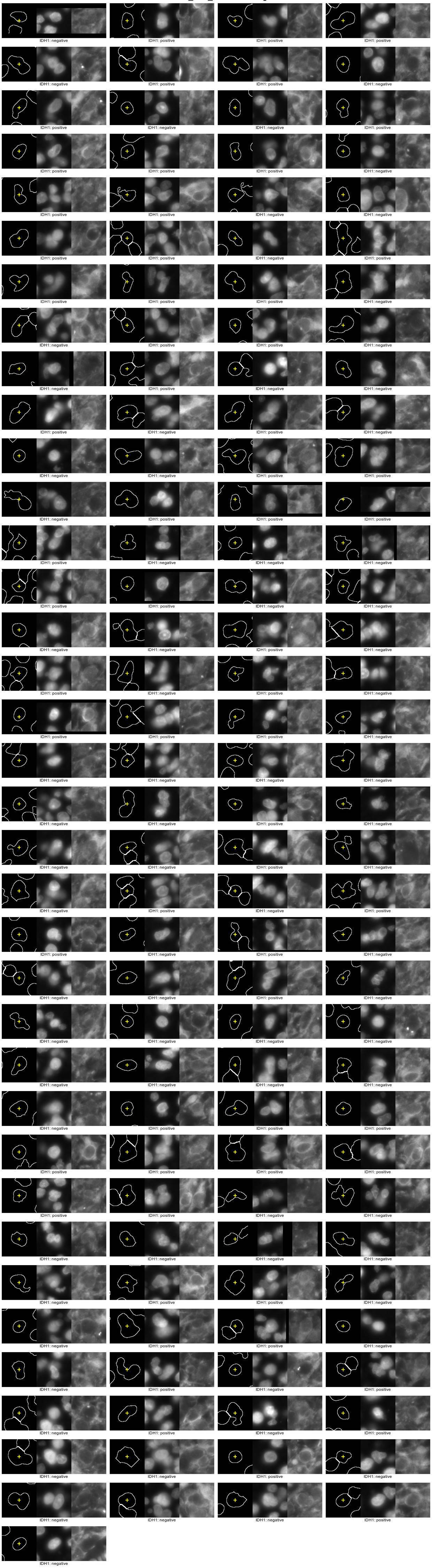

Case4_ROI_1 ZEB1 scoring

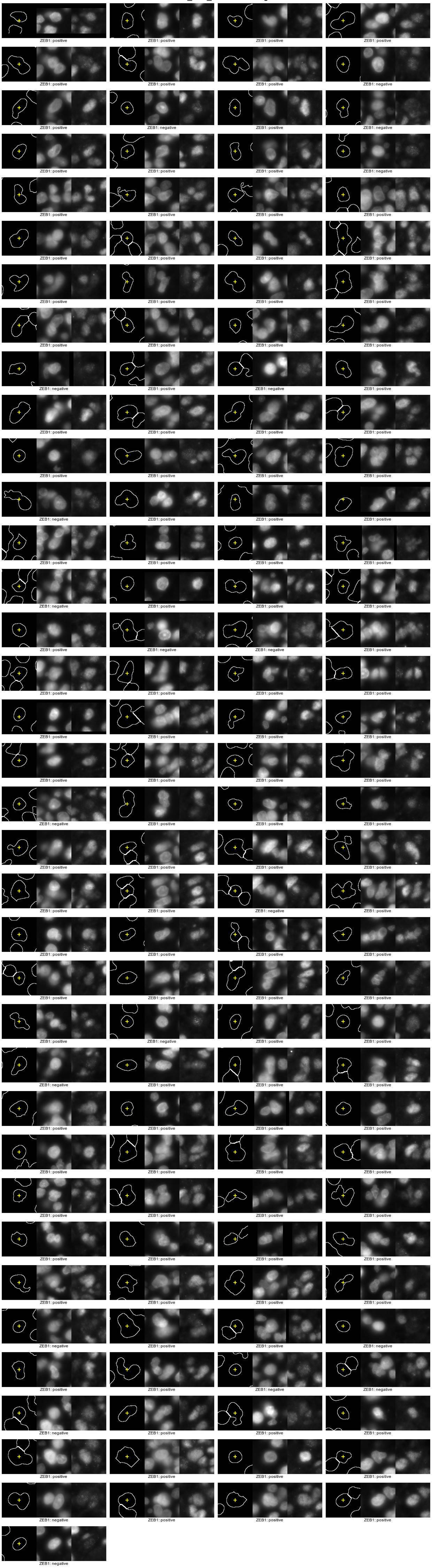

Case4_ROI_10 overview

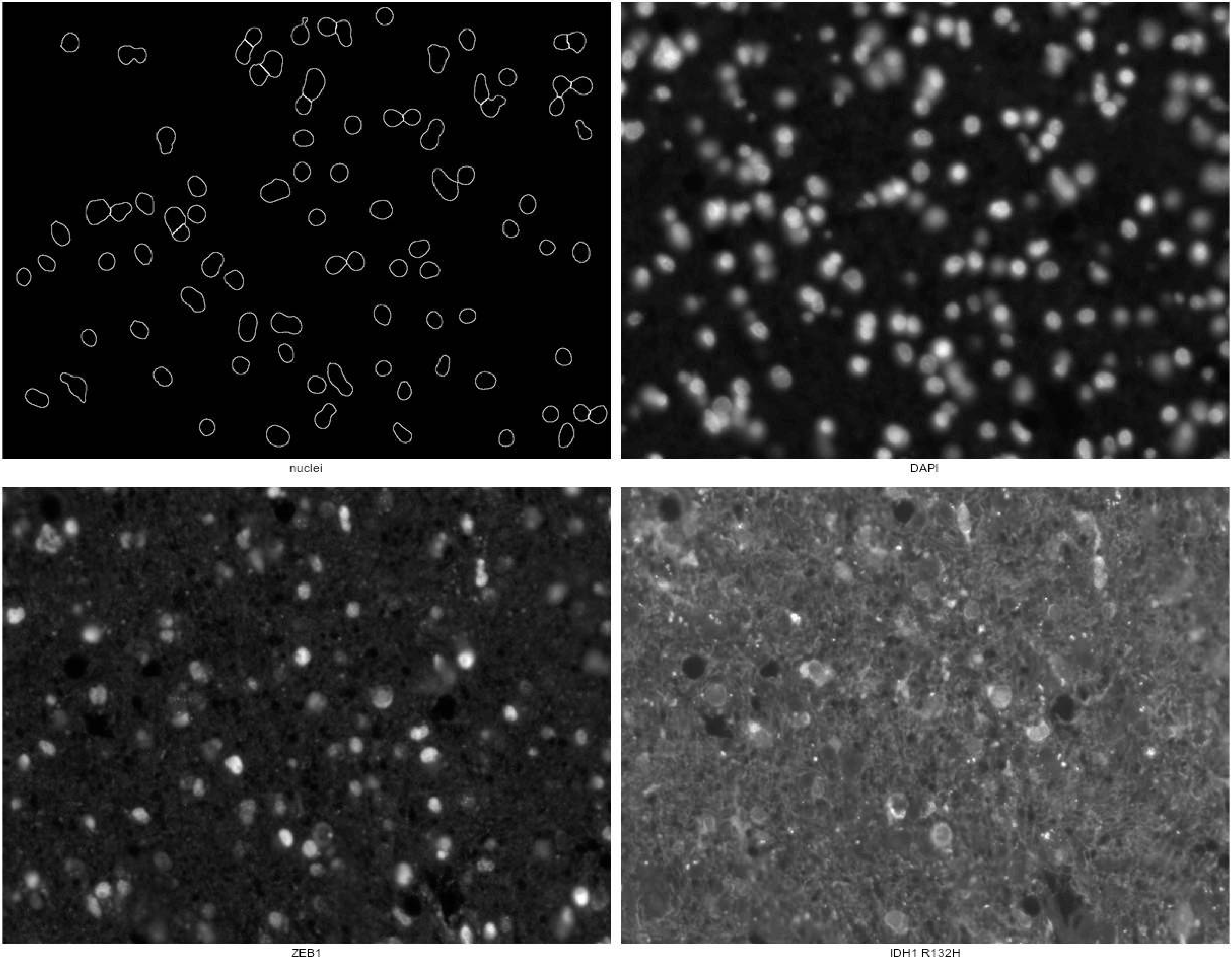

Case4_ROI_10 IDH1 scoring

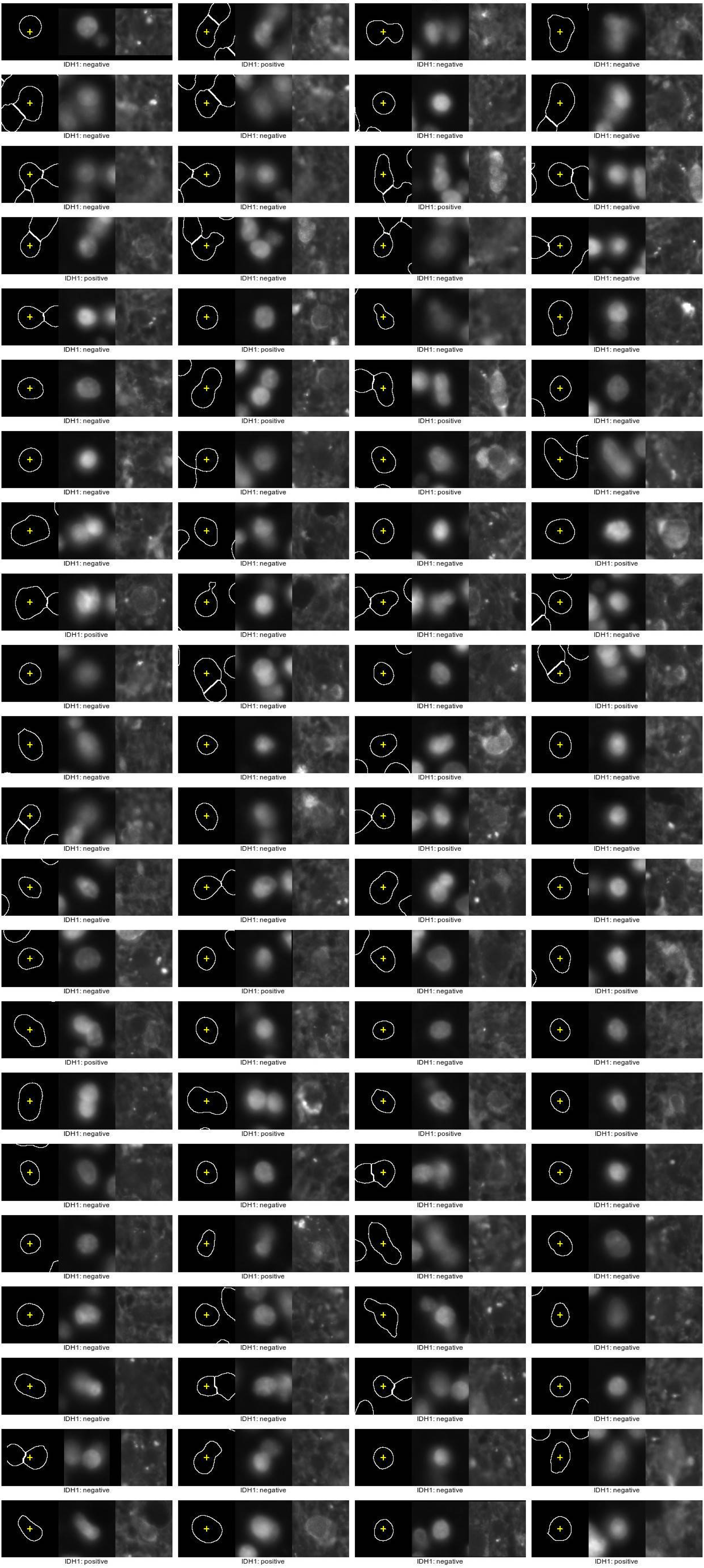

Case4_ROI_10 ZEB1 scoring

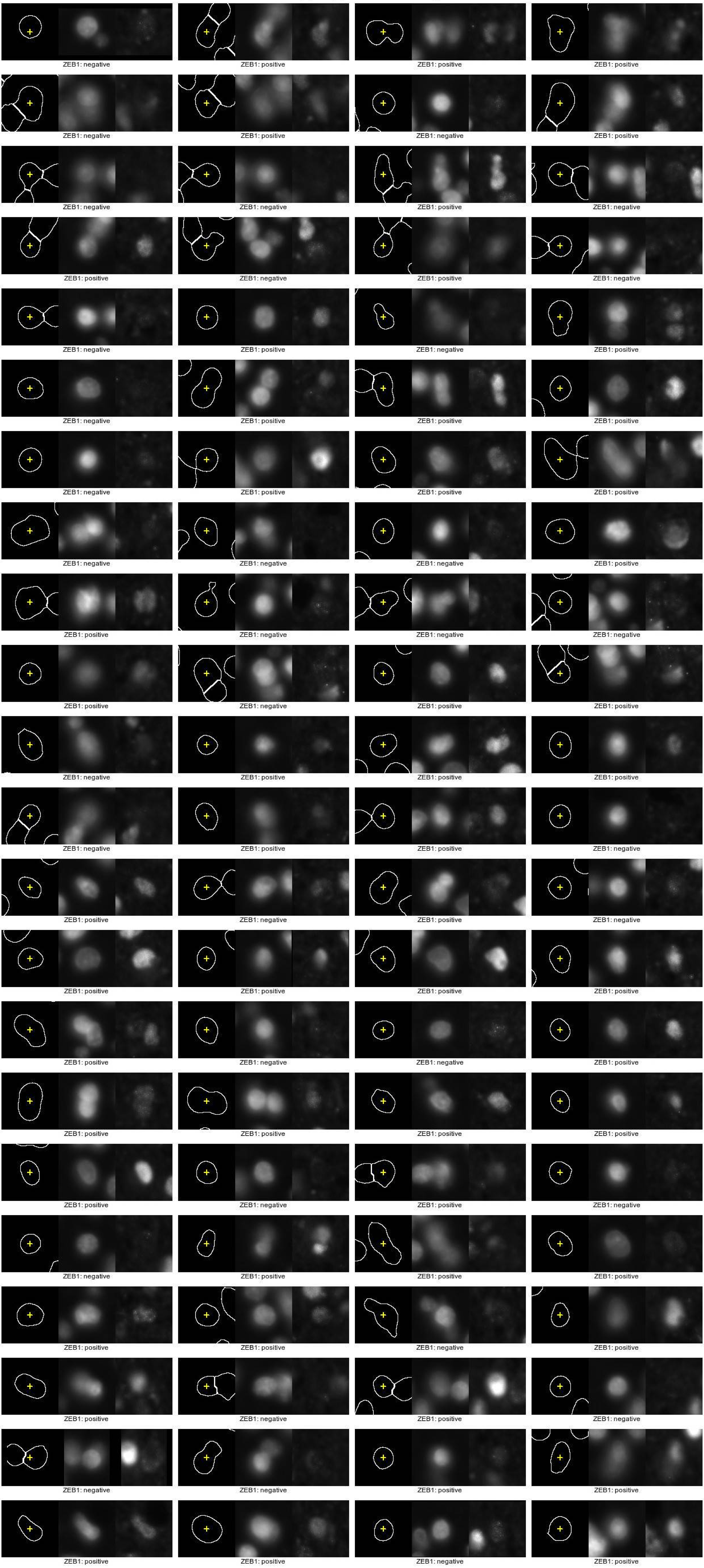

Case4_ROI_2 overview

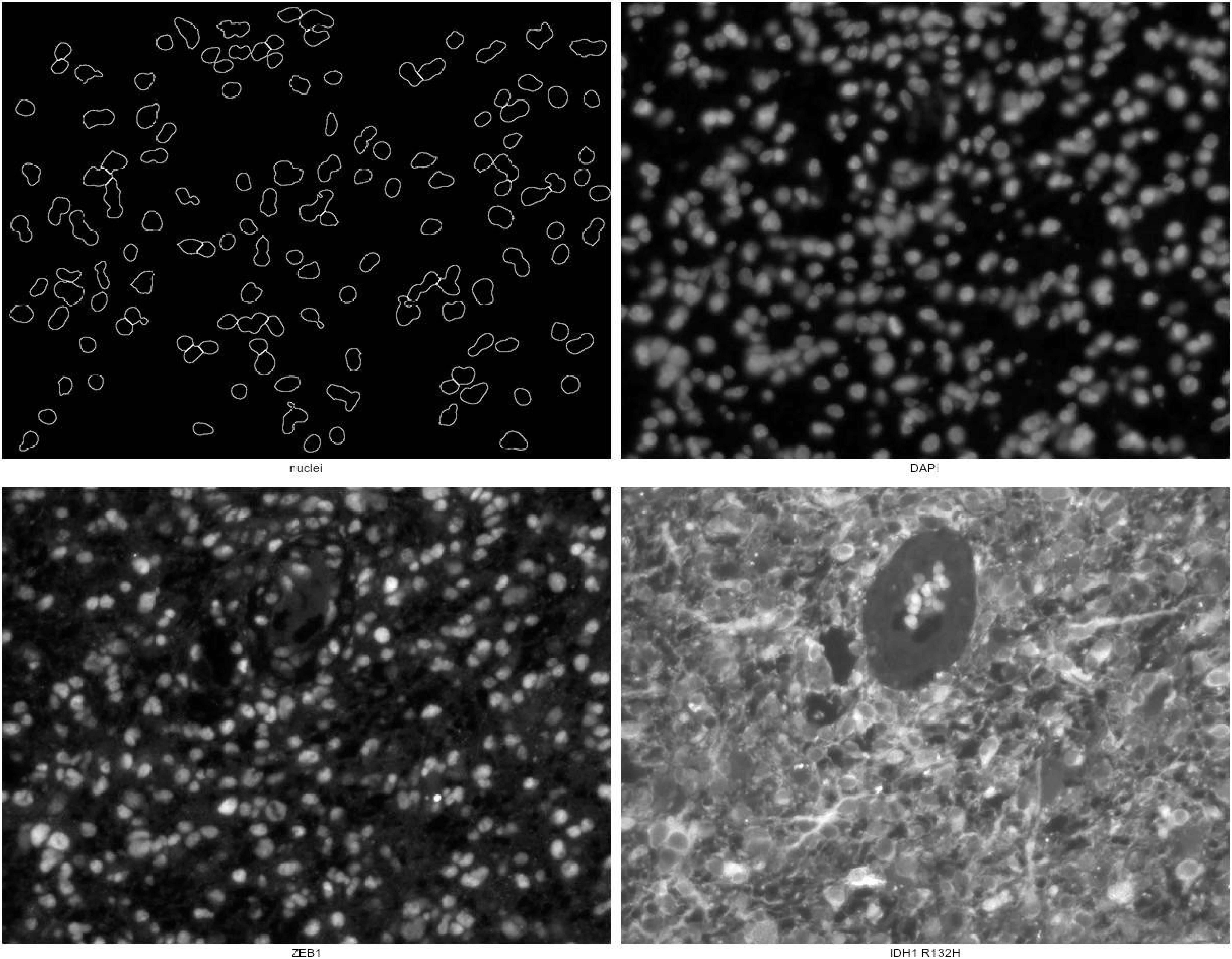

Case4_ROI_2 IDH1 scoring

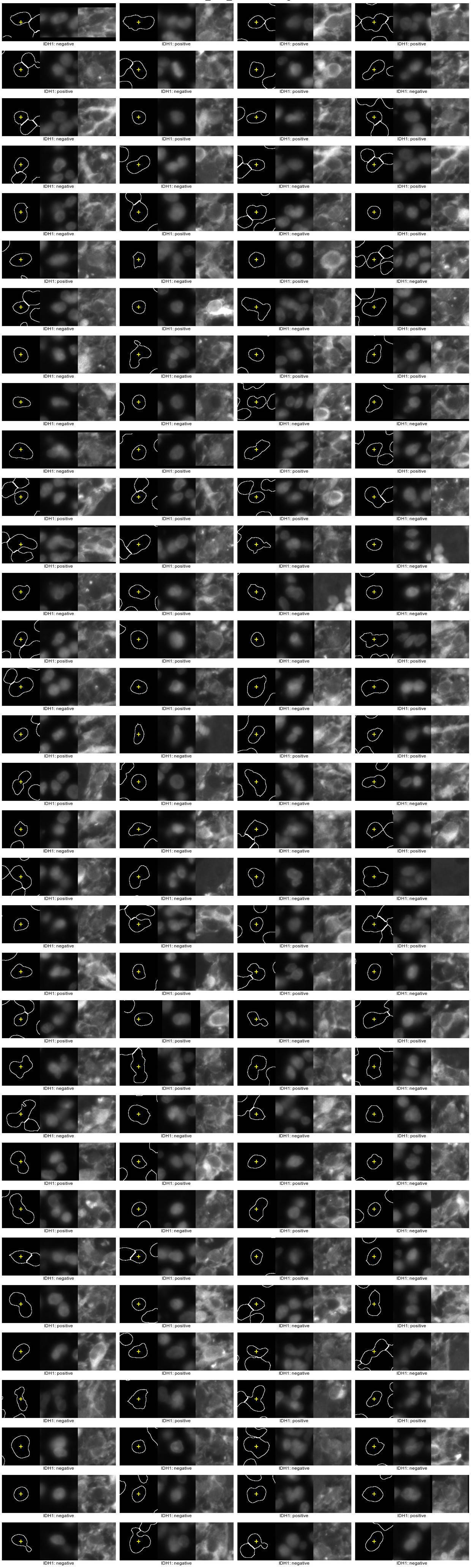

Case4_ROI_2 ZEB1 scoring

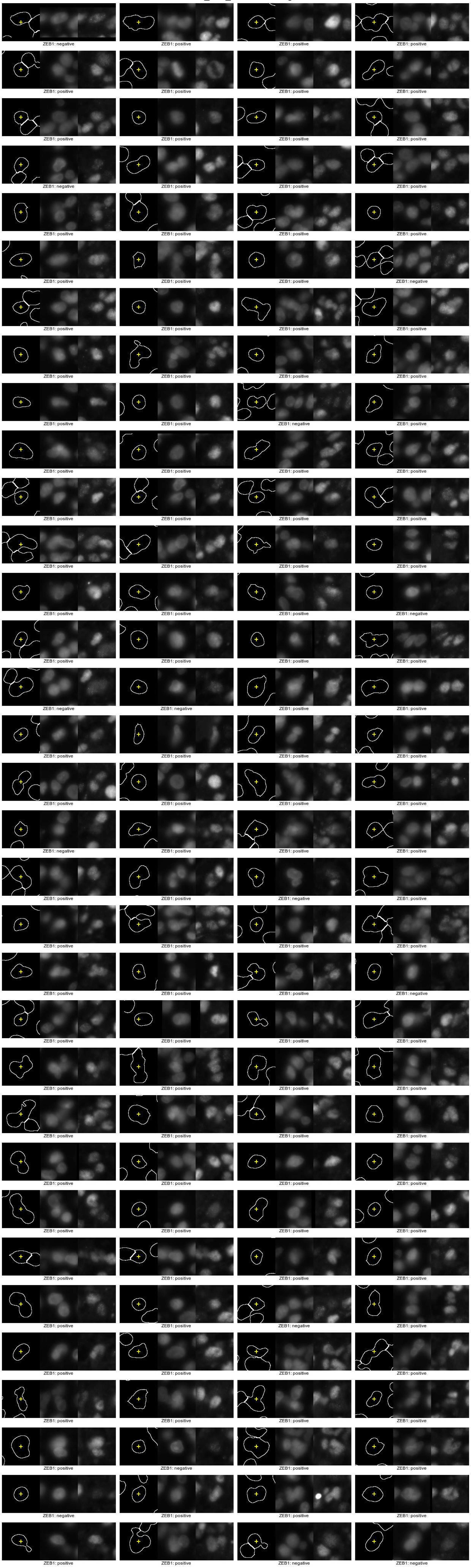

Case4_ROI_3 overview

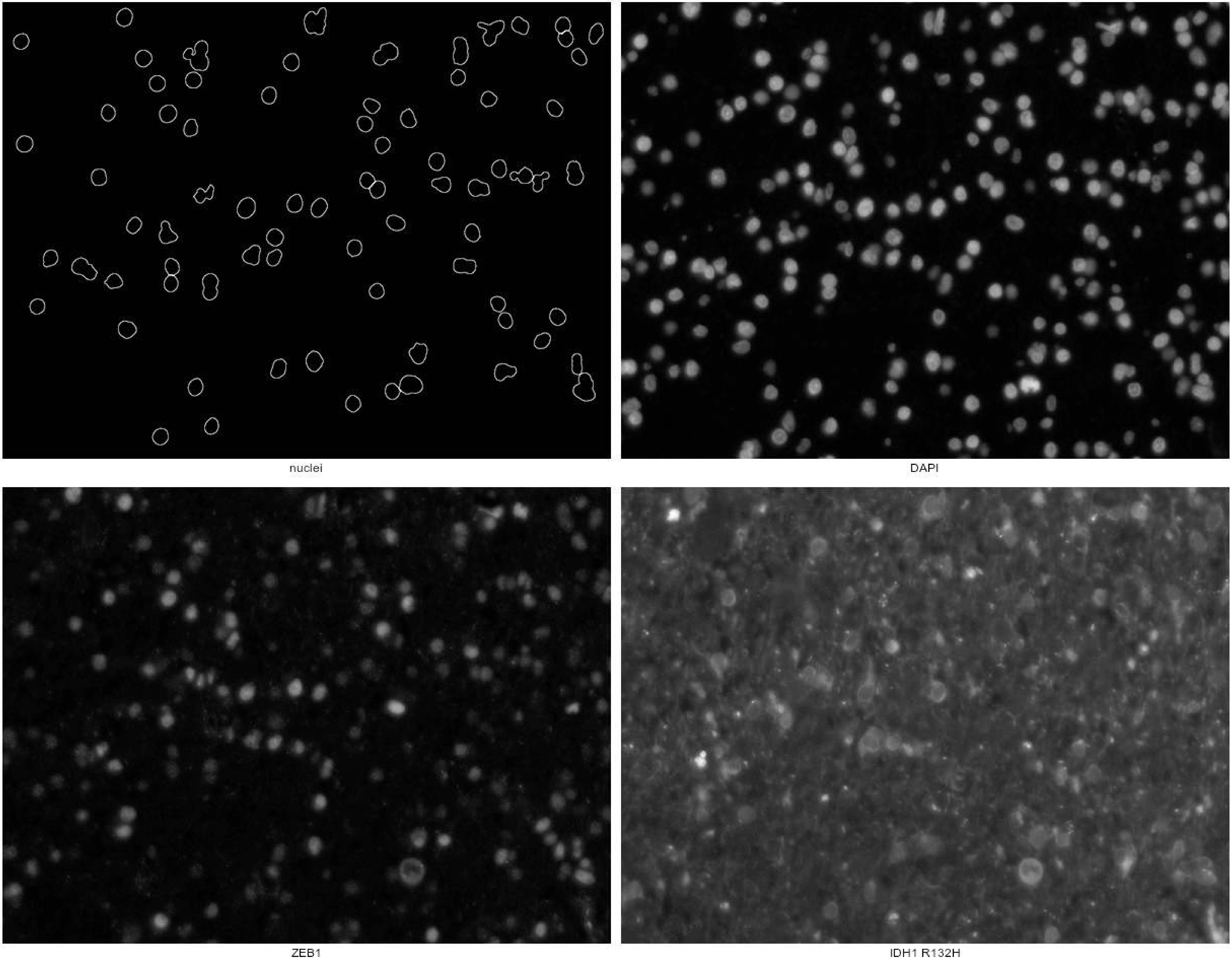

Case4_ROI_3 IDH1 scoring

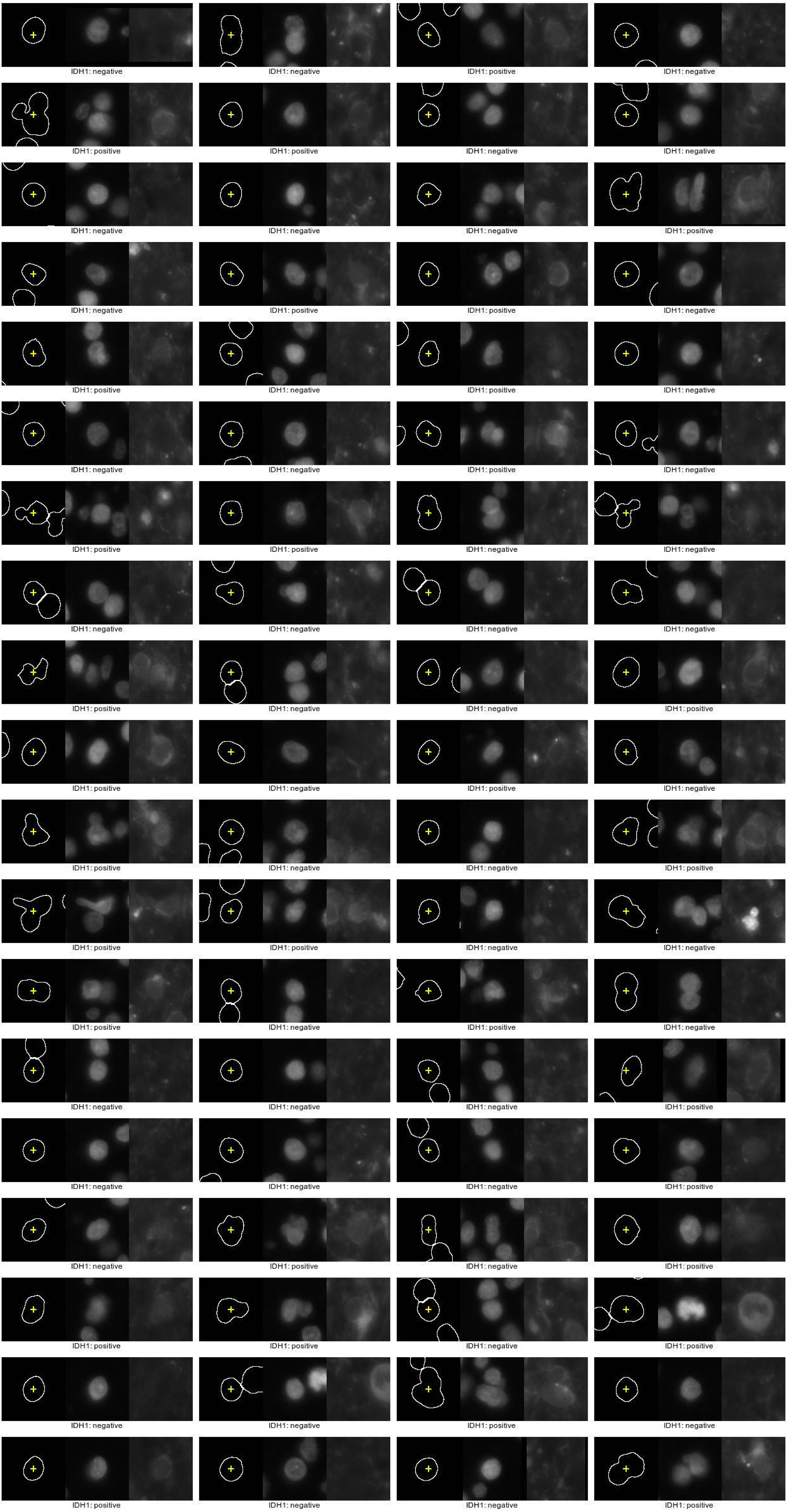

Case4_ROI_3 ZEB1 scoring

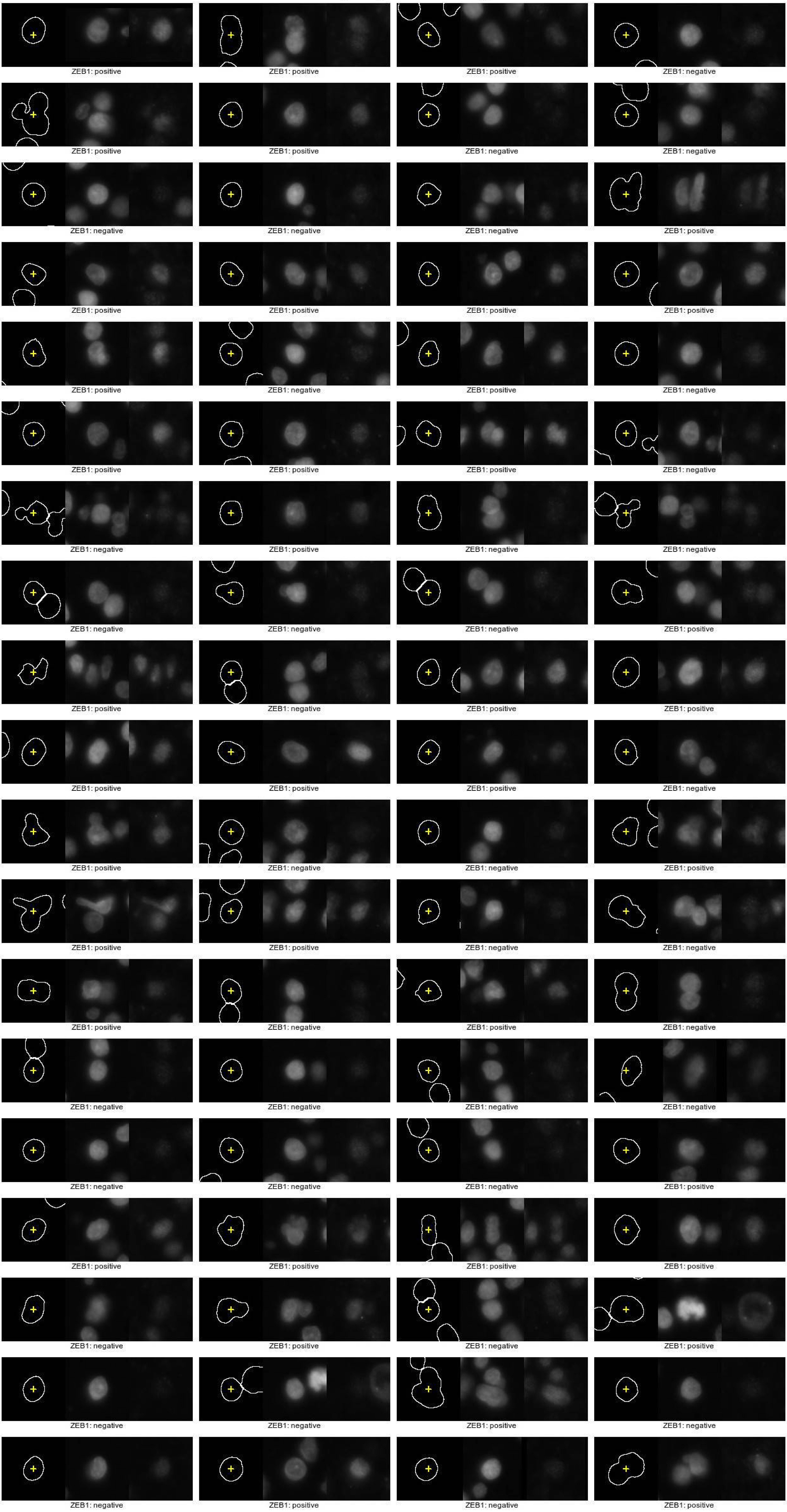

Case4_ROI_4 overview

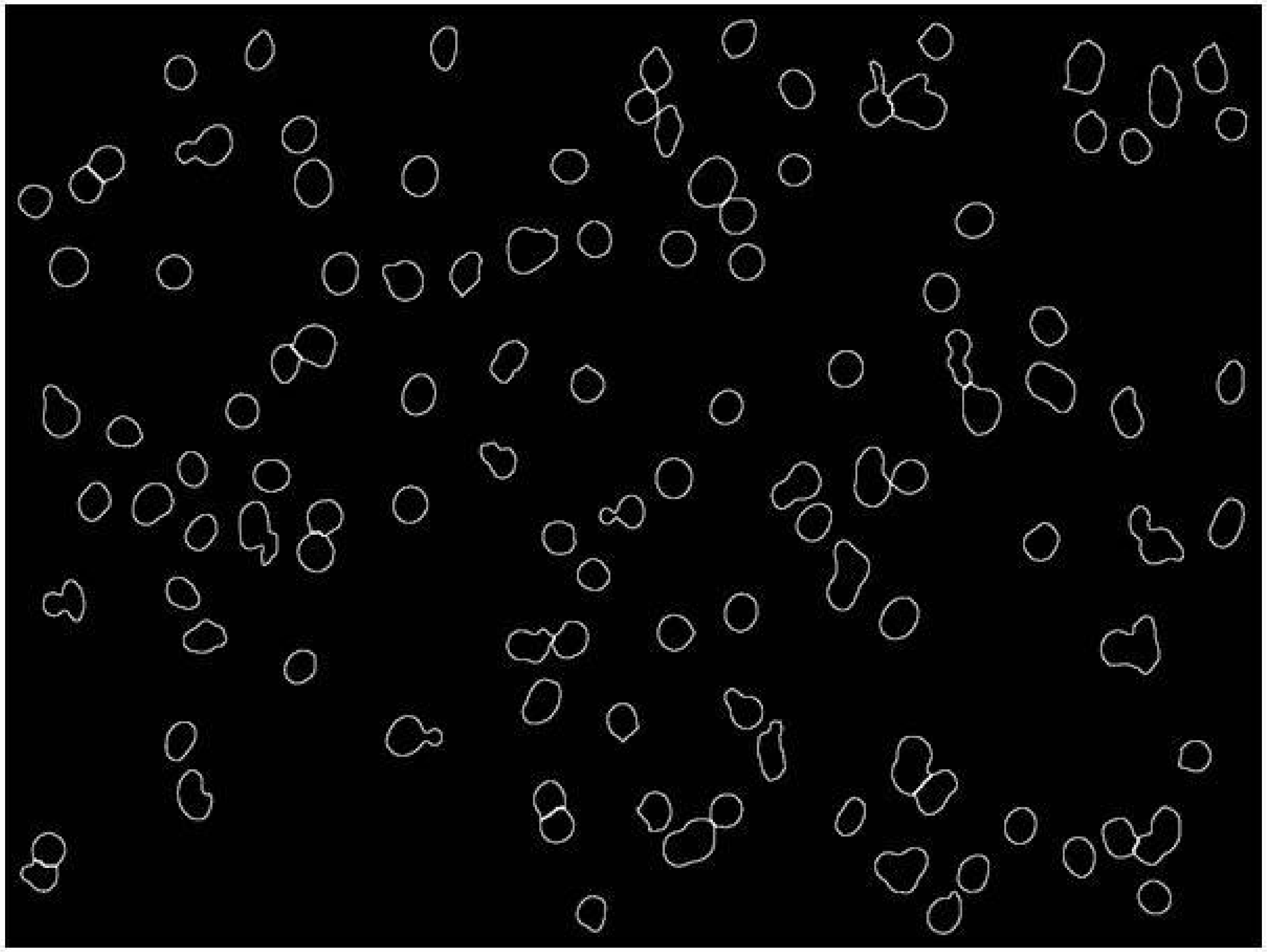

nuclei

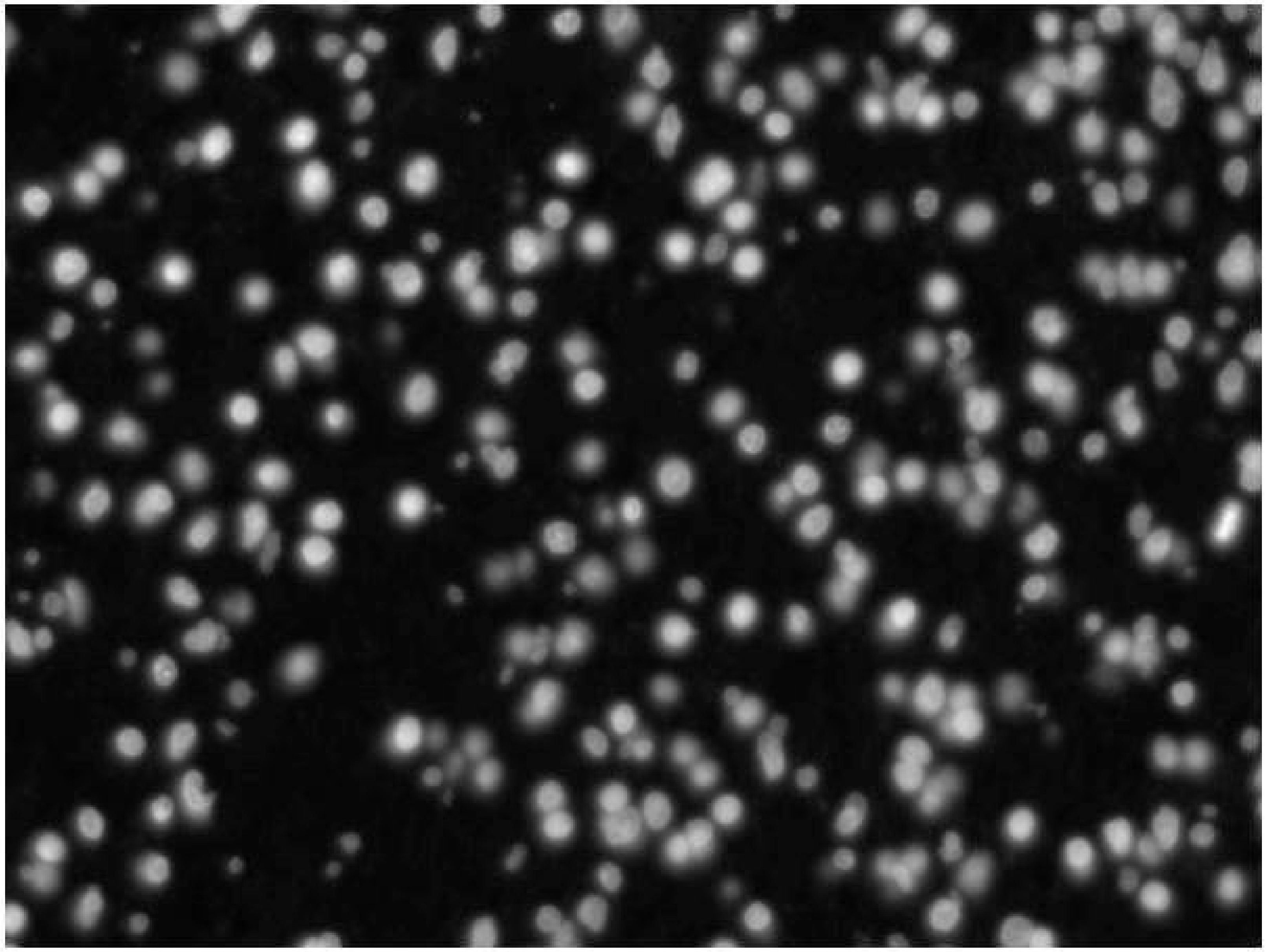

DAPI

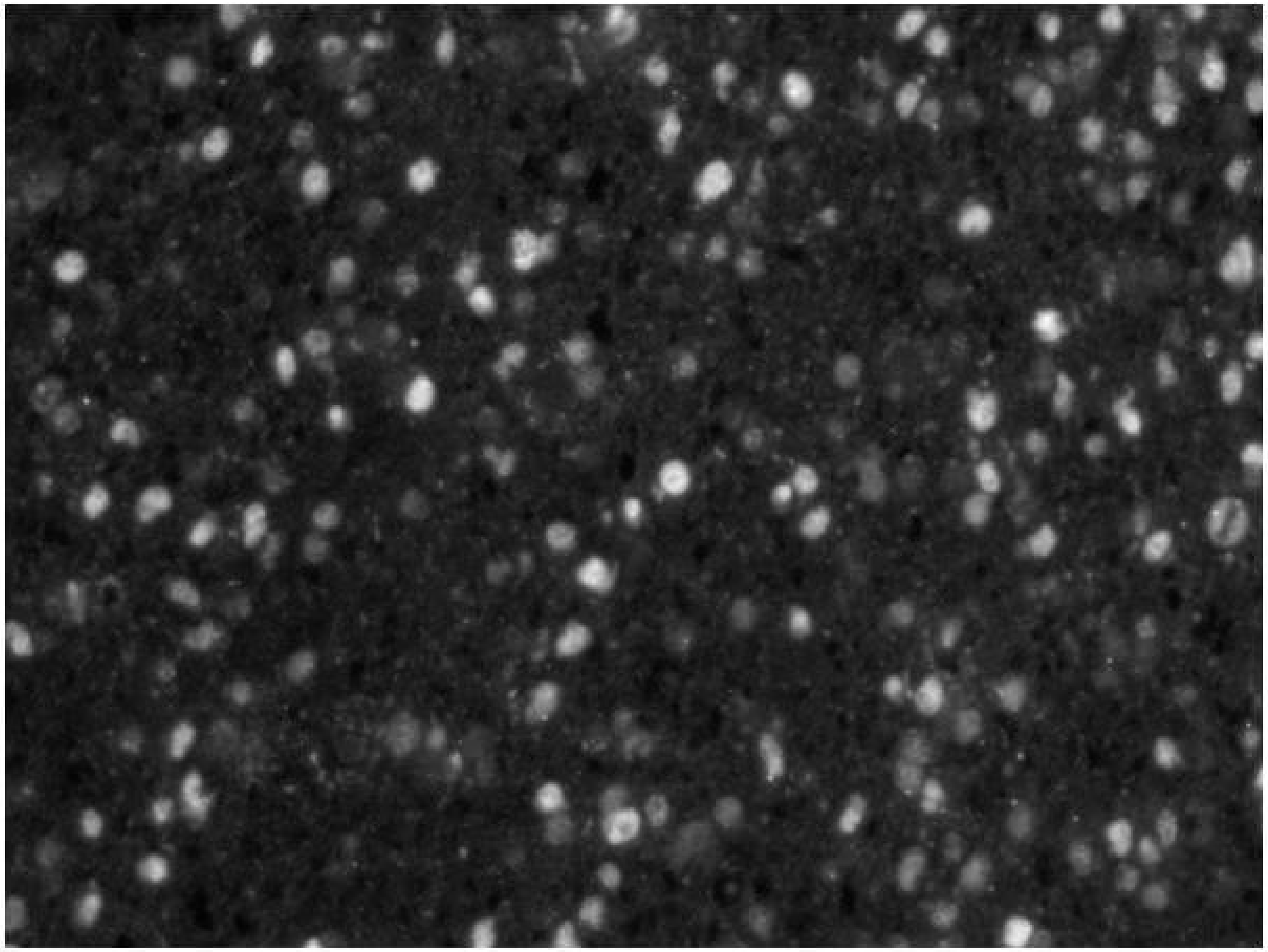

ZEB1

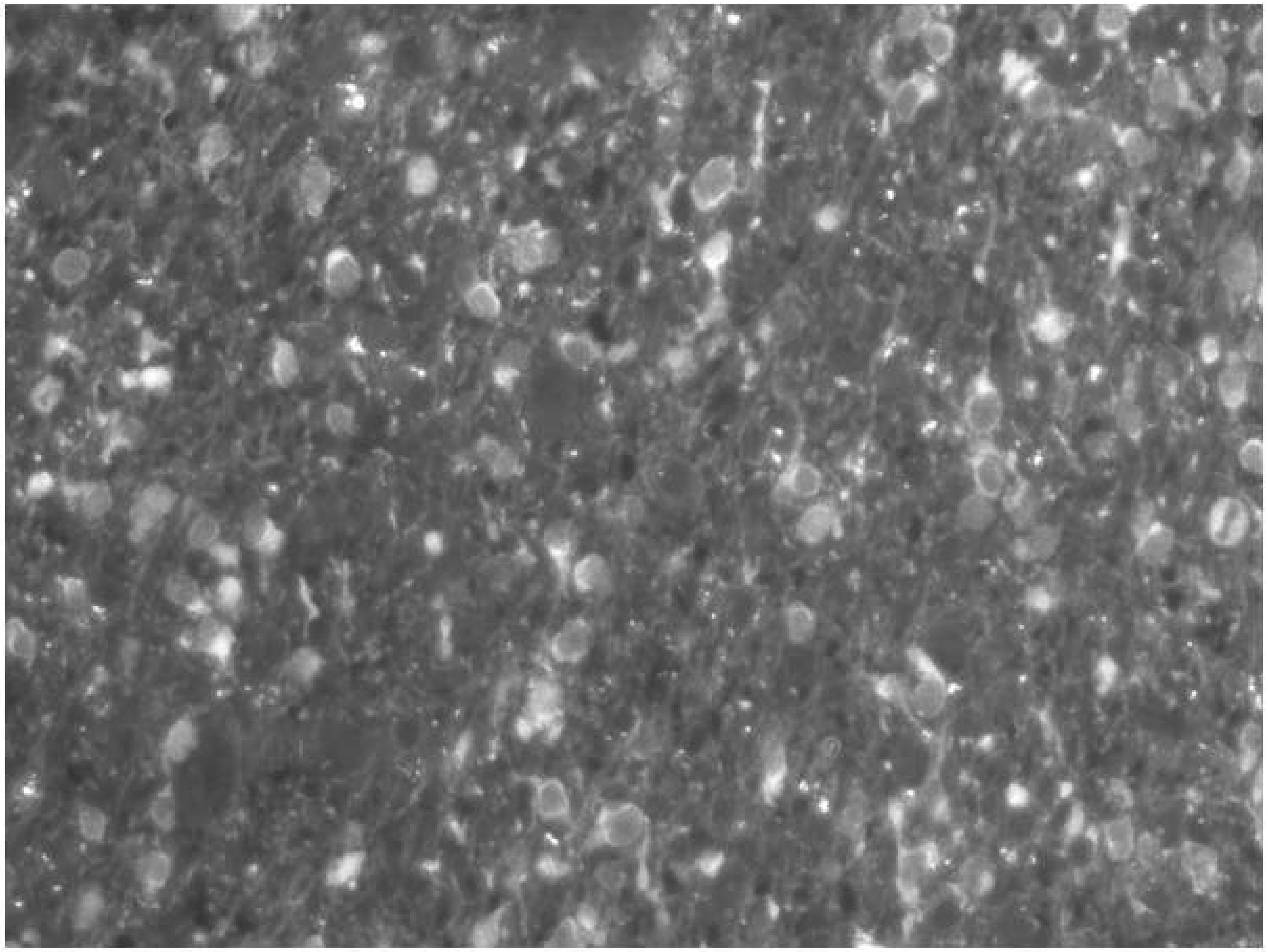

IDH1 R132H

Case4_ROI_4 IDH1 scoring

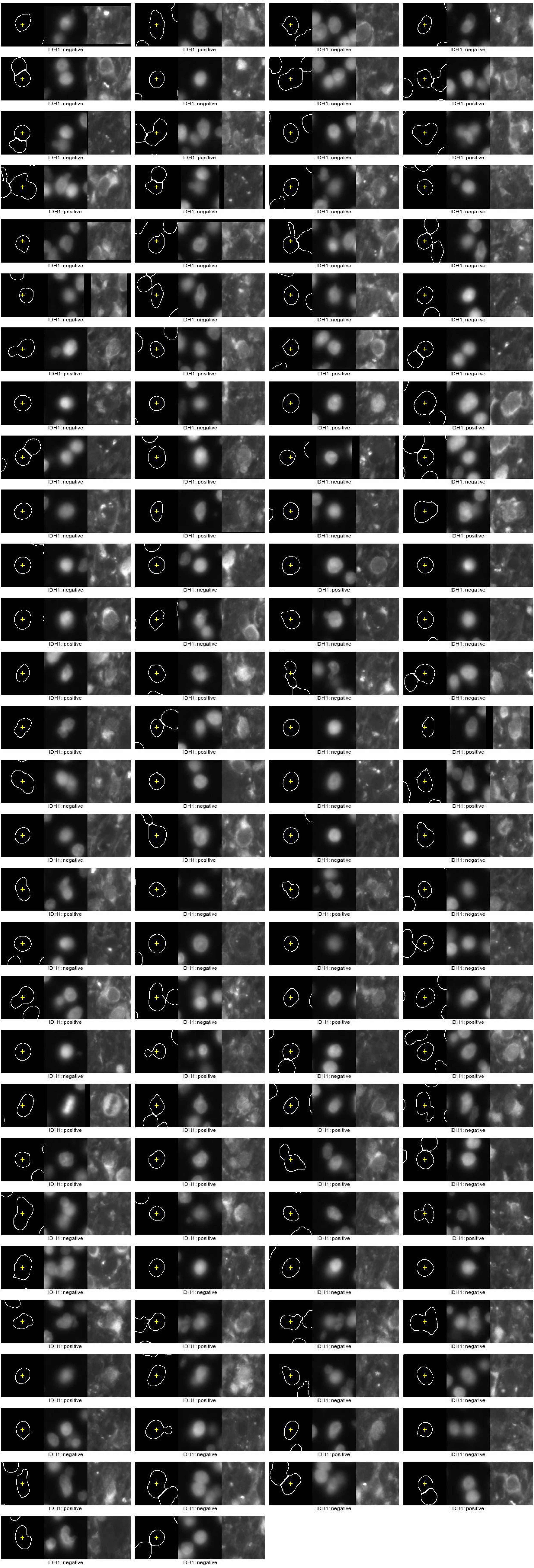

Case4_ROI_4 ZEB1 scoring

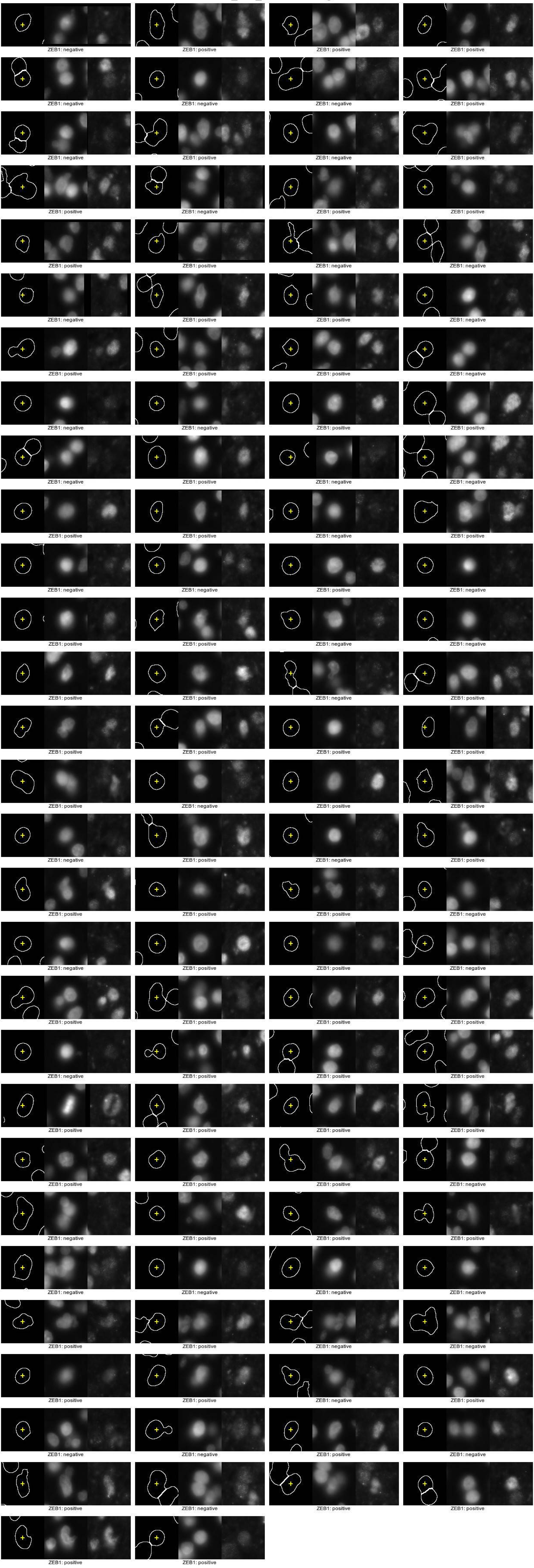

Case4_ROI_5 overview

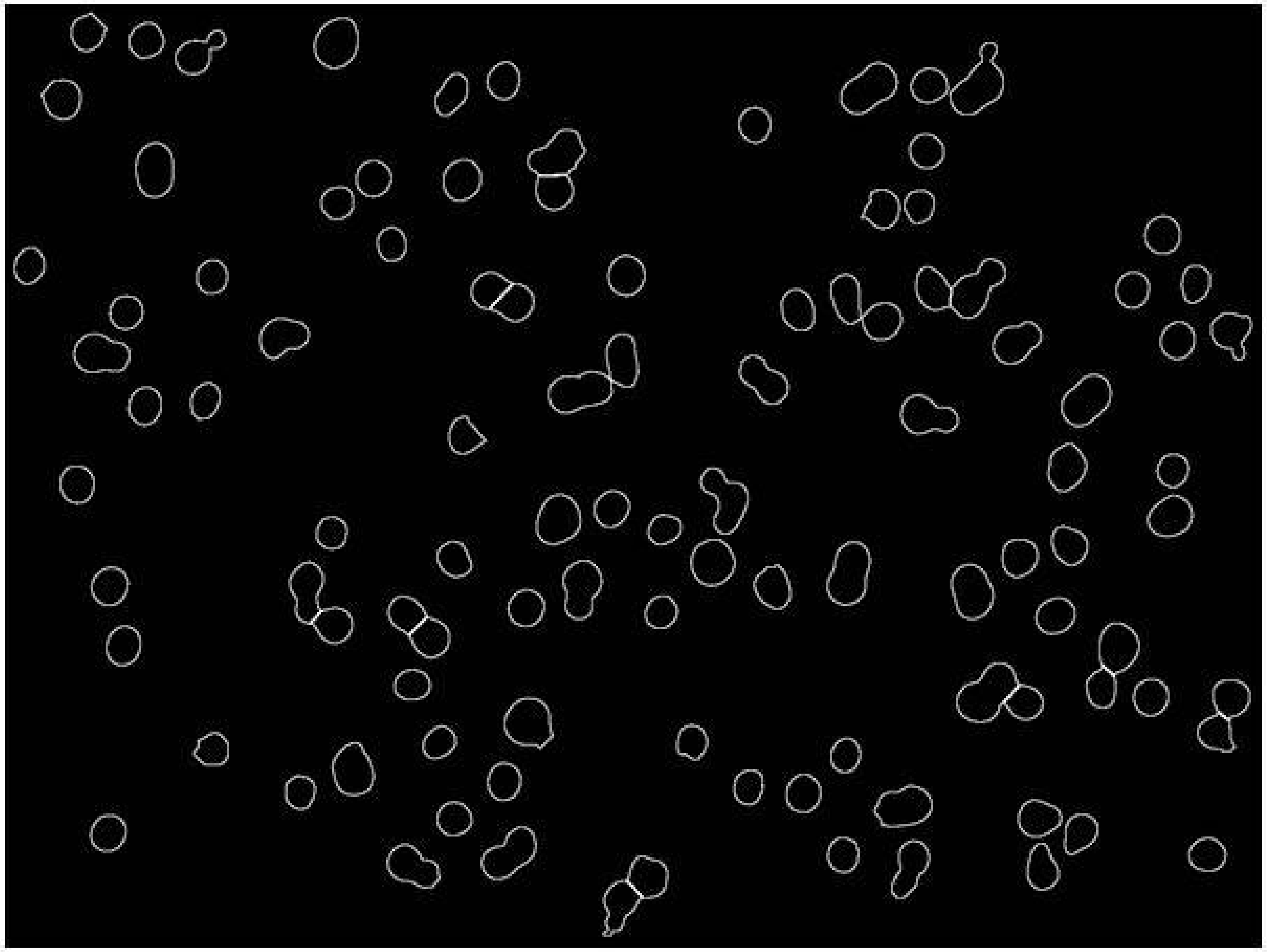

nuclei

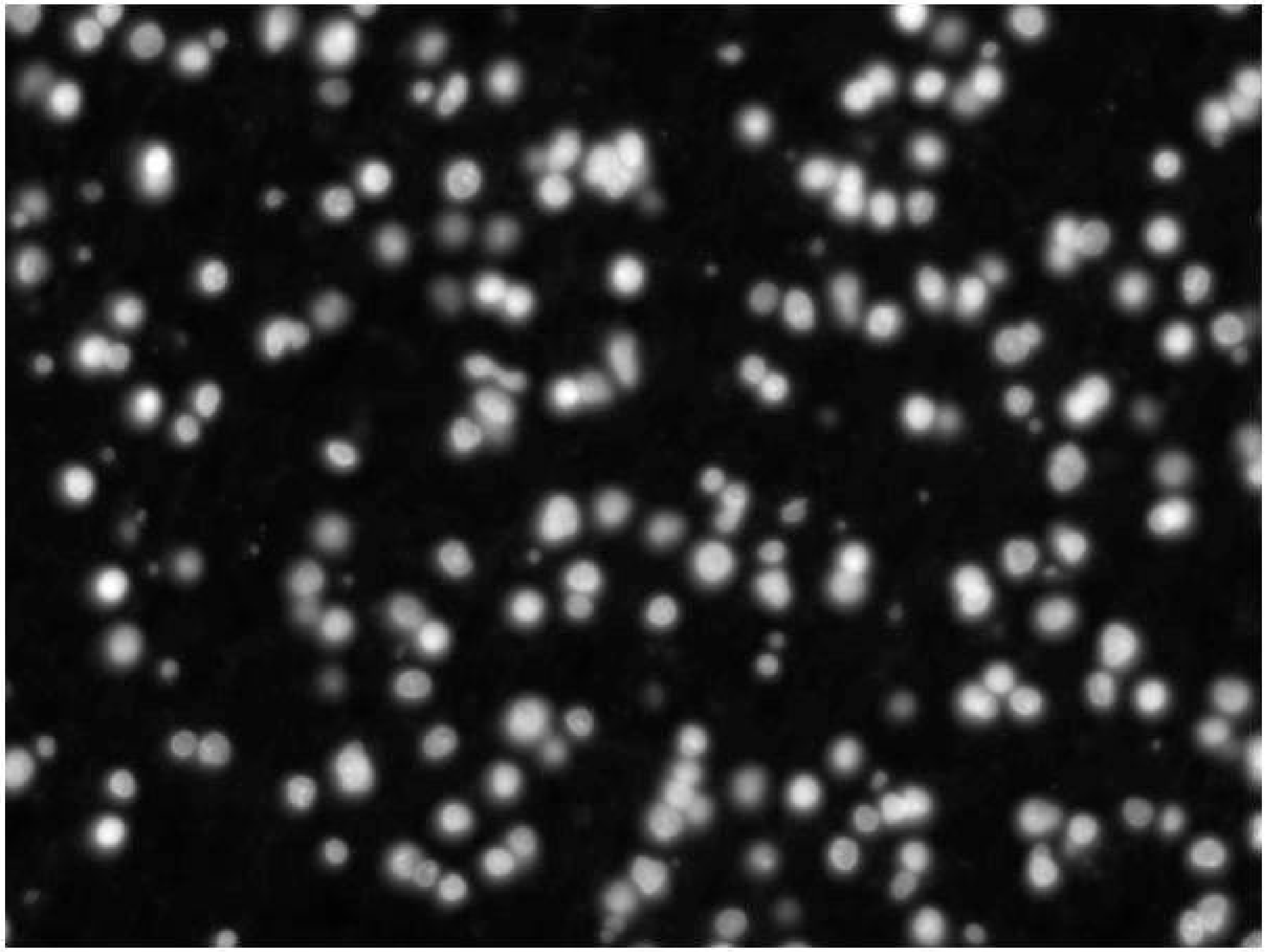

DAPI

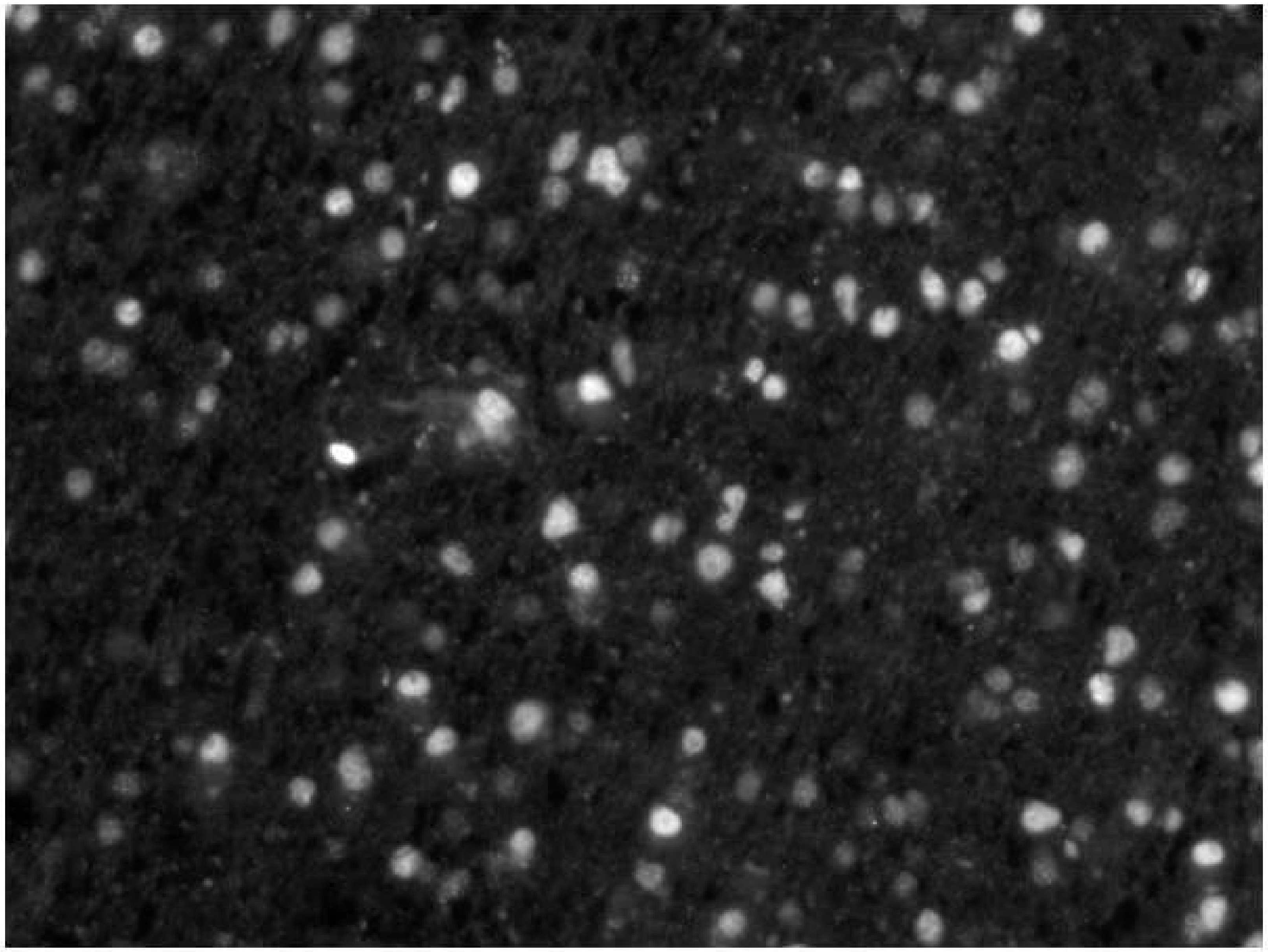

ZEB1

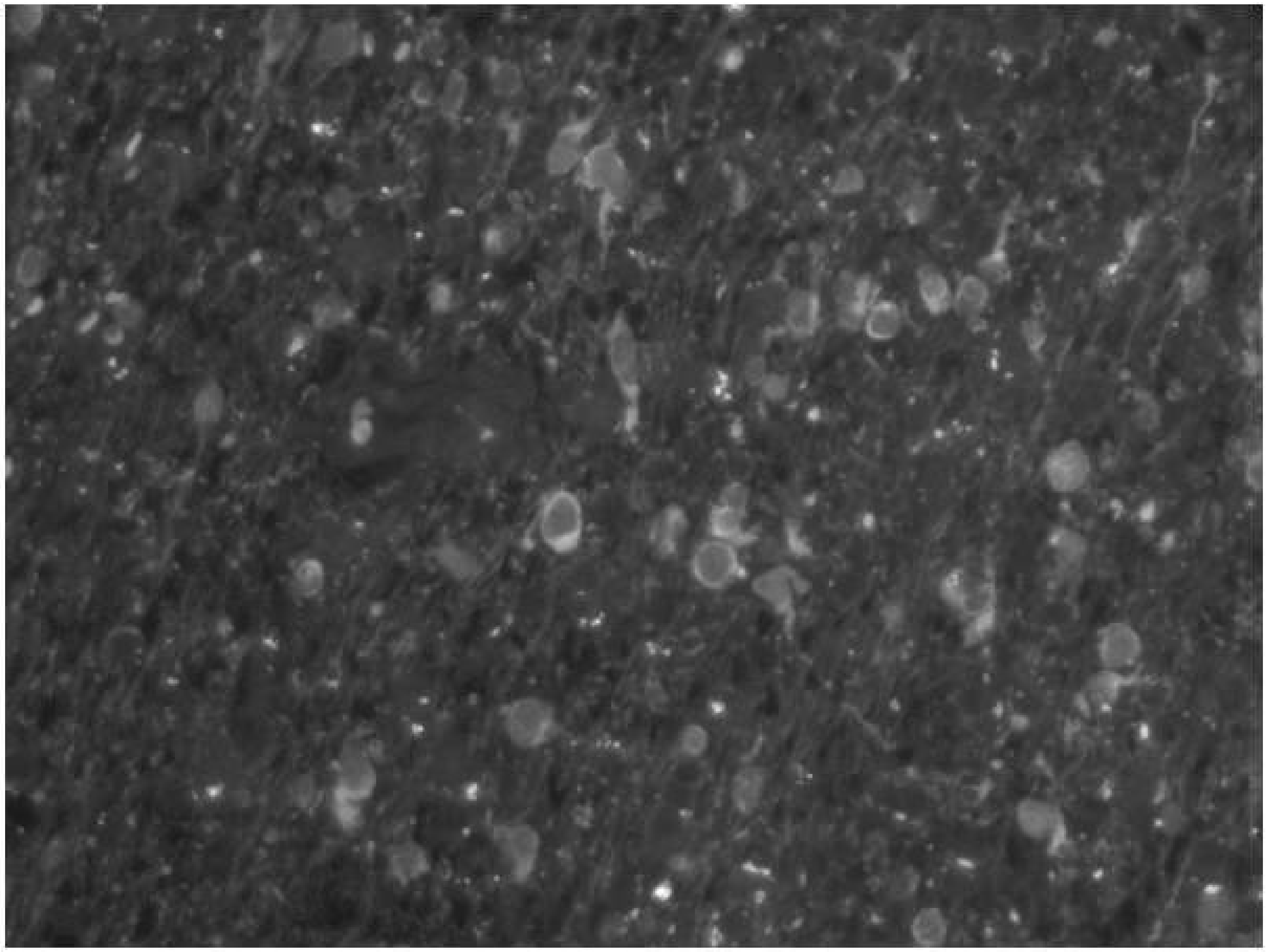

IDH1 R132H

Case4_ROI_5 IDH1 scoring

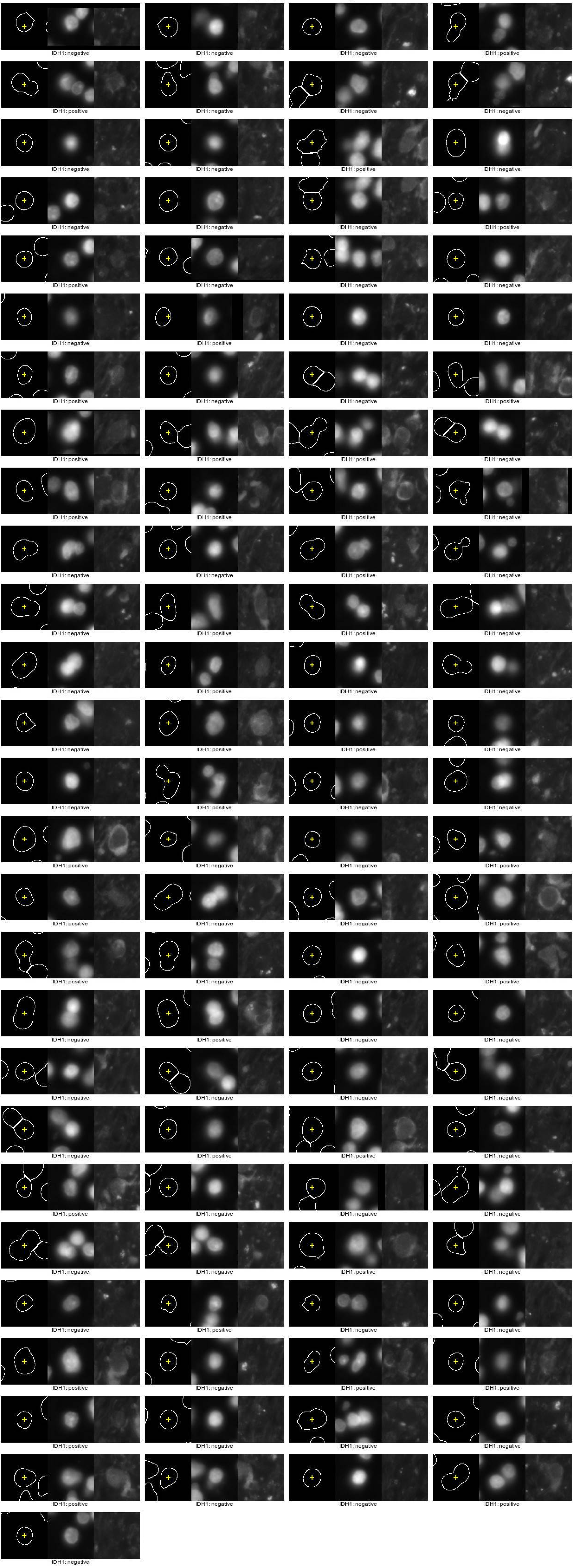

Case4_ROI_5 ZEB1 scoring

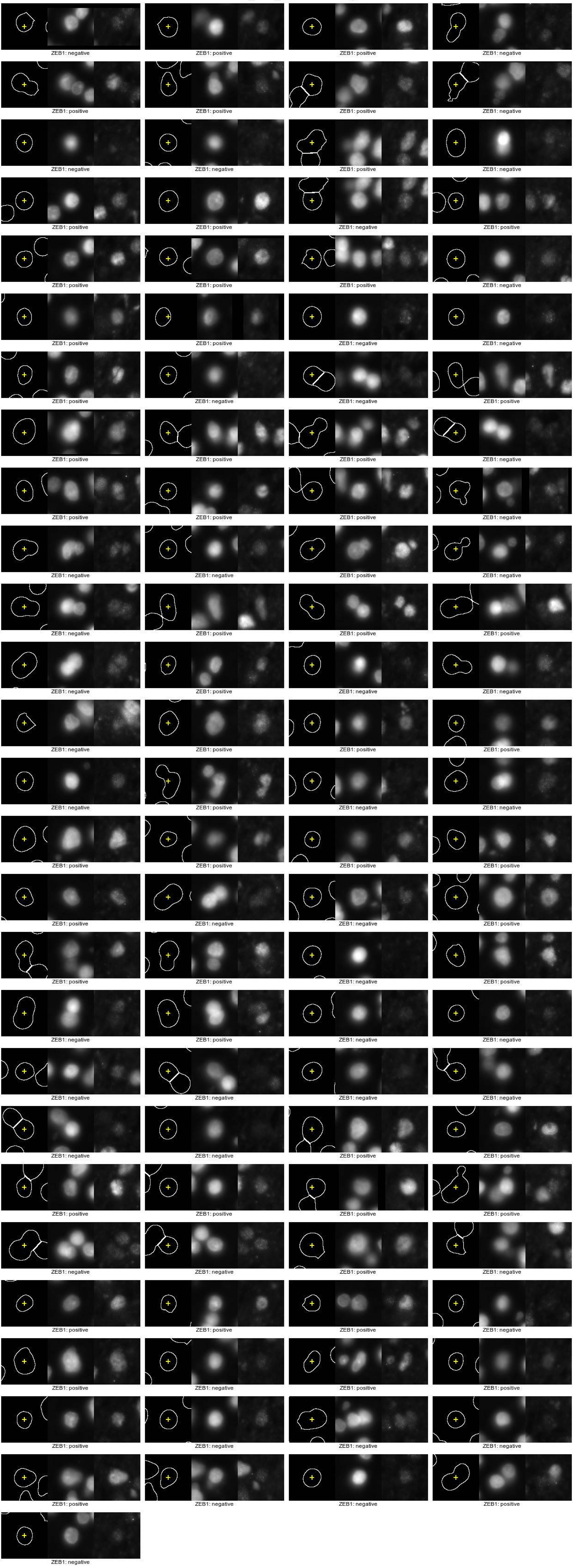

Case4_ROI_6 overview

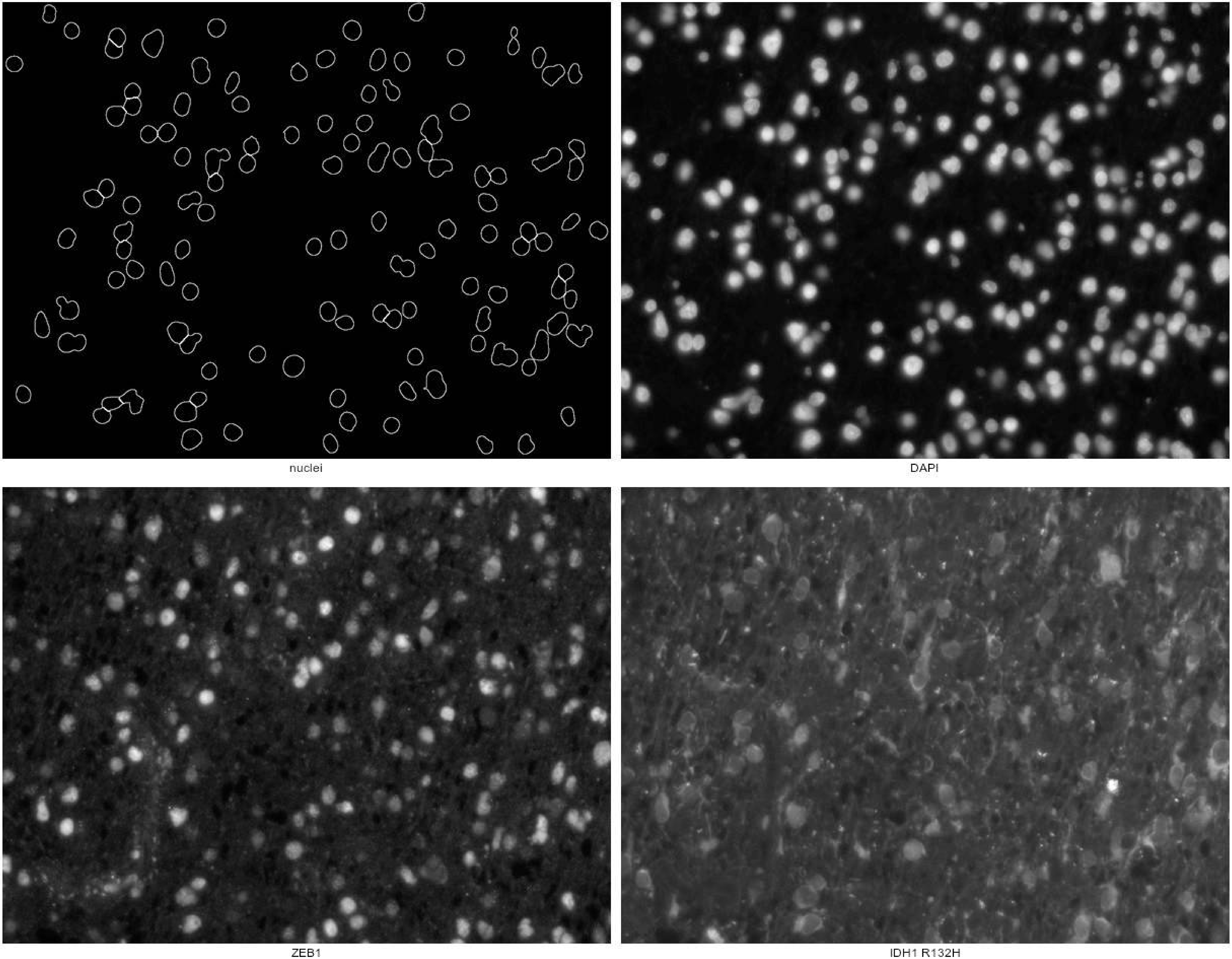

Case4_ROI_6 IDH1 scoring

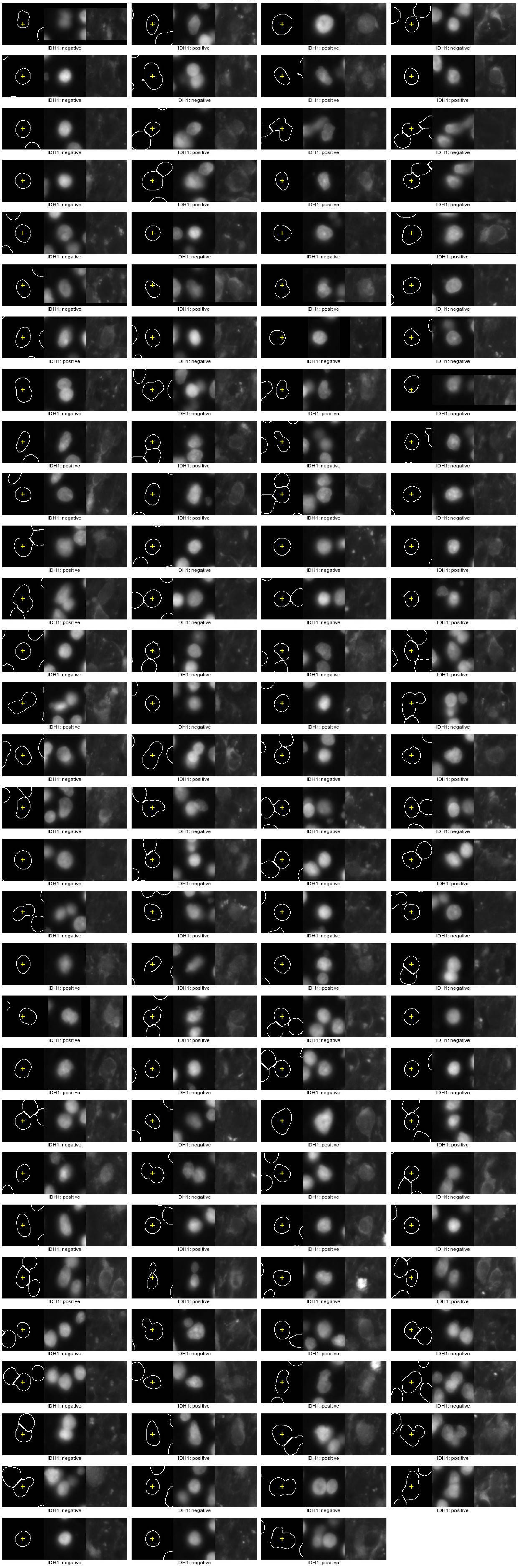

Case4_ROI_6 ZEB1 scoring

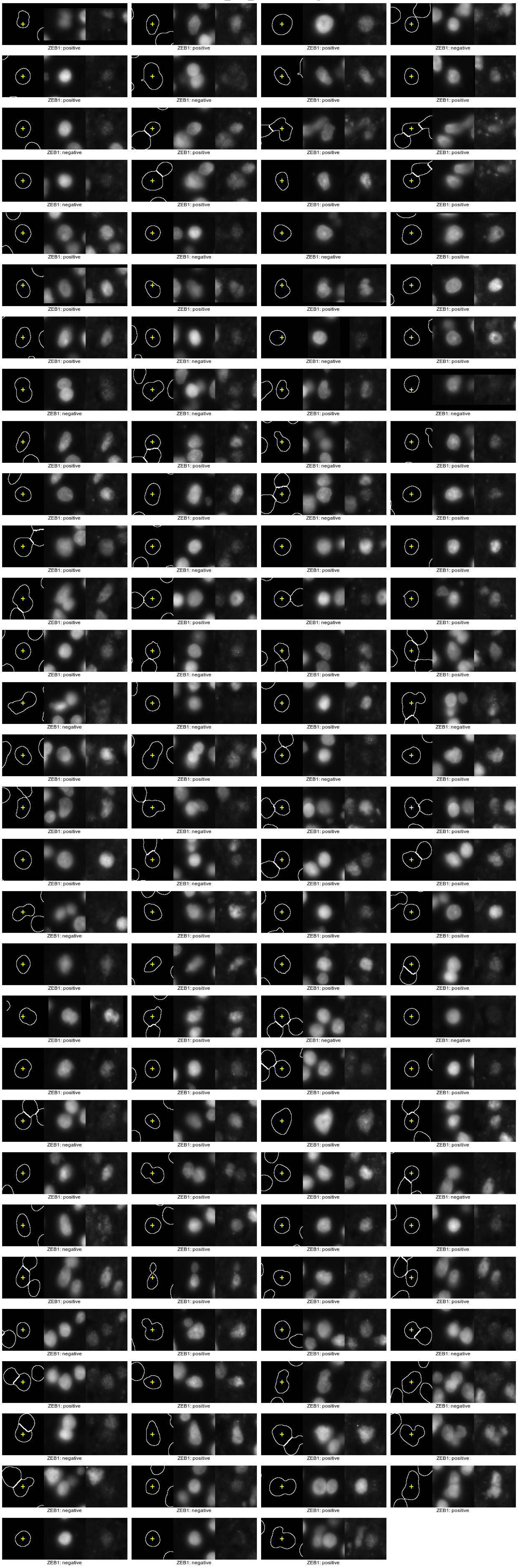

Case4_ROI_7 overview

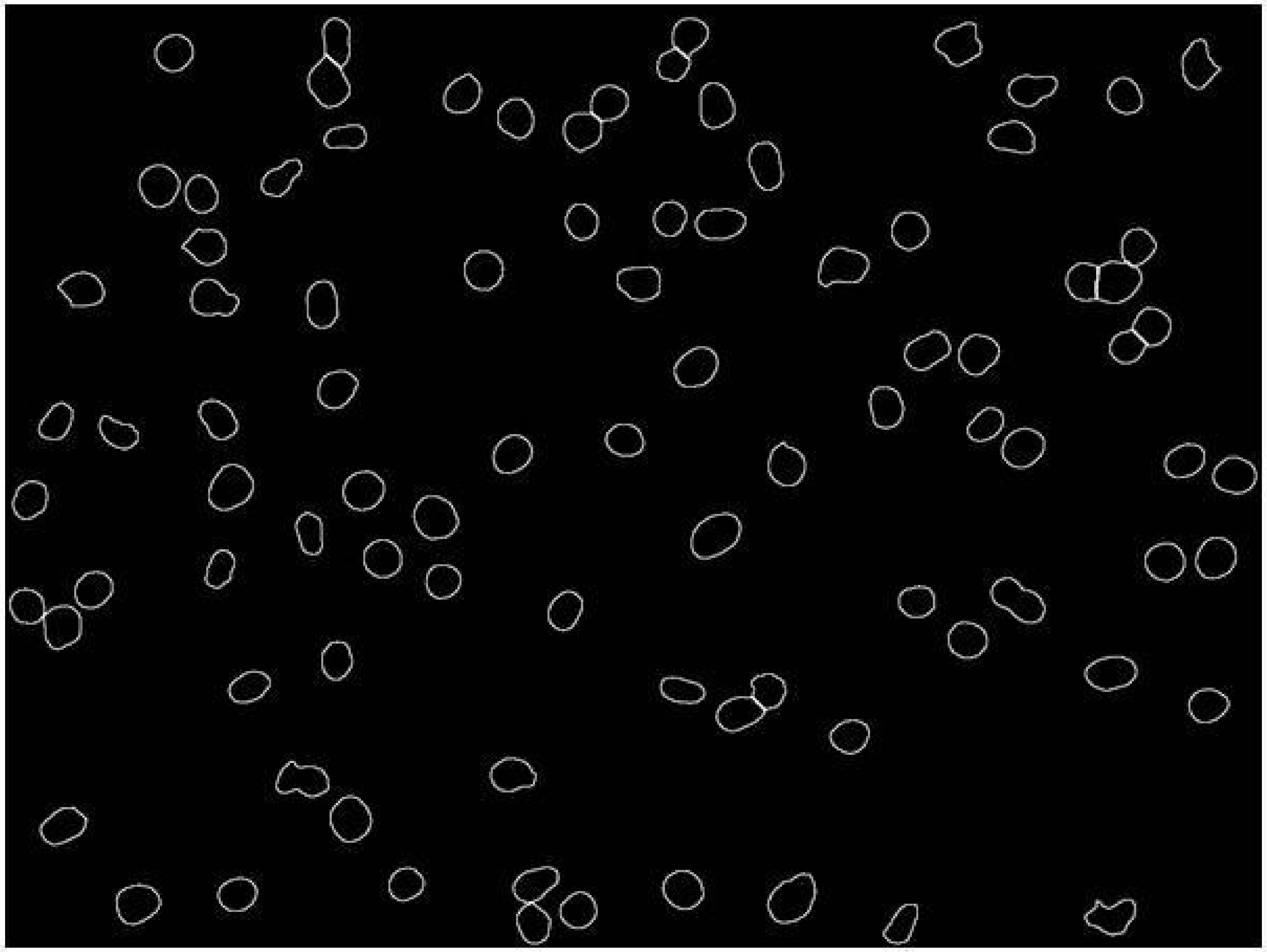

nuclei

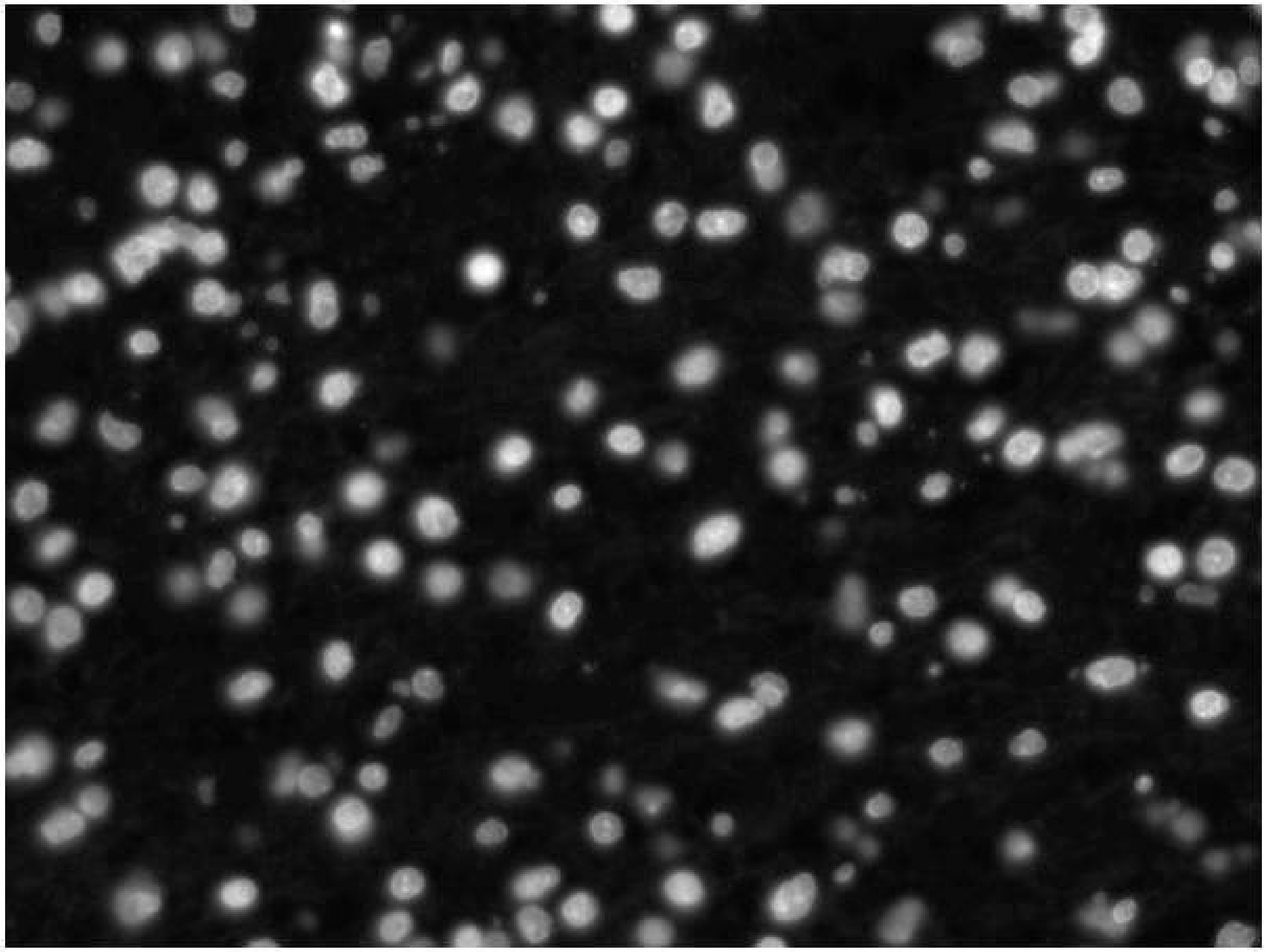

DAPI

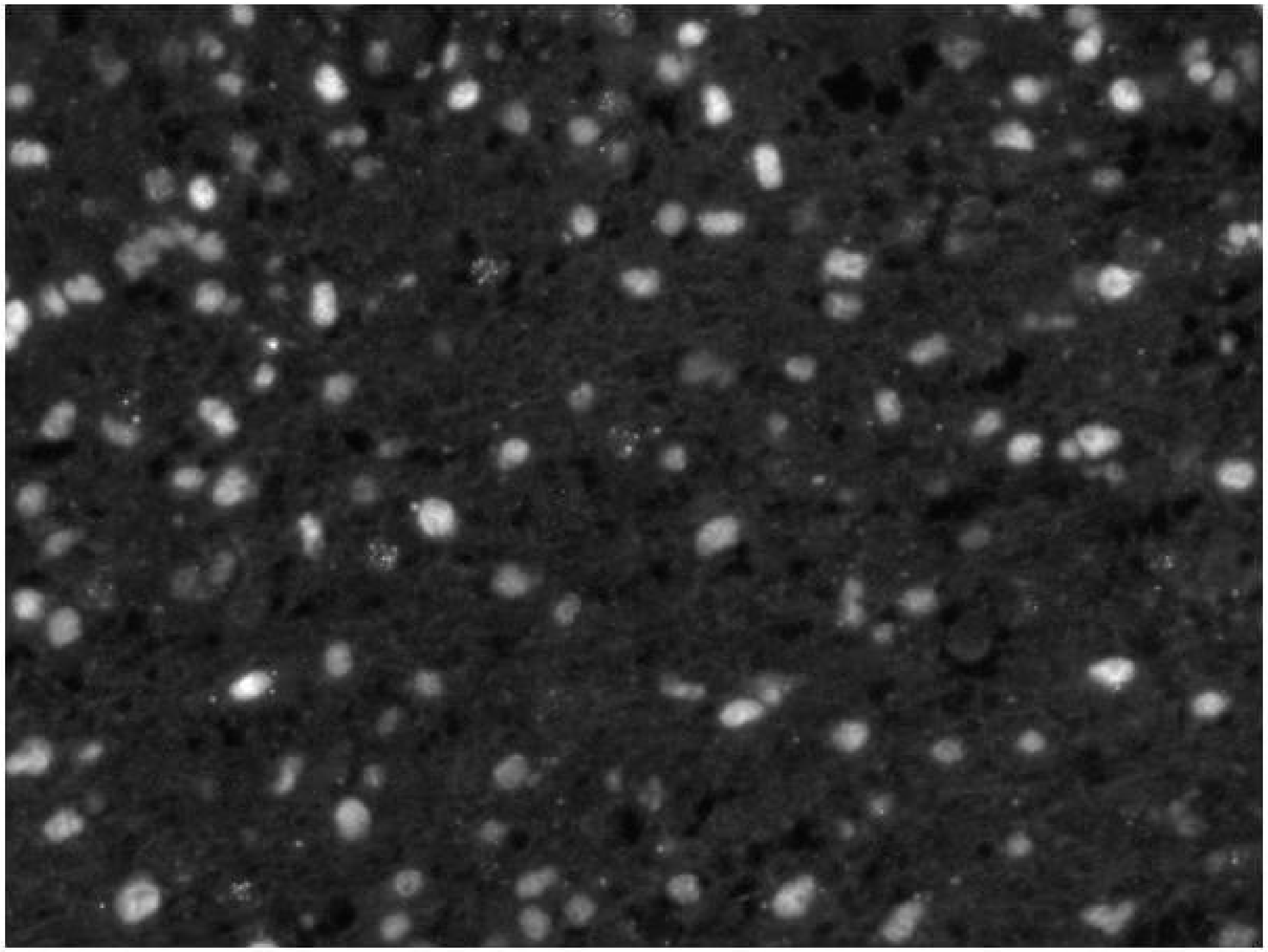

ZEB1

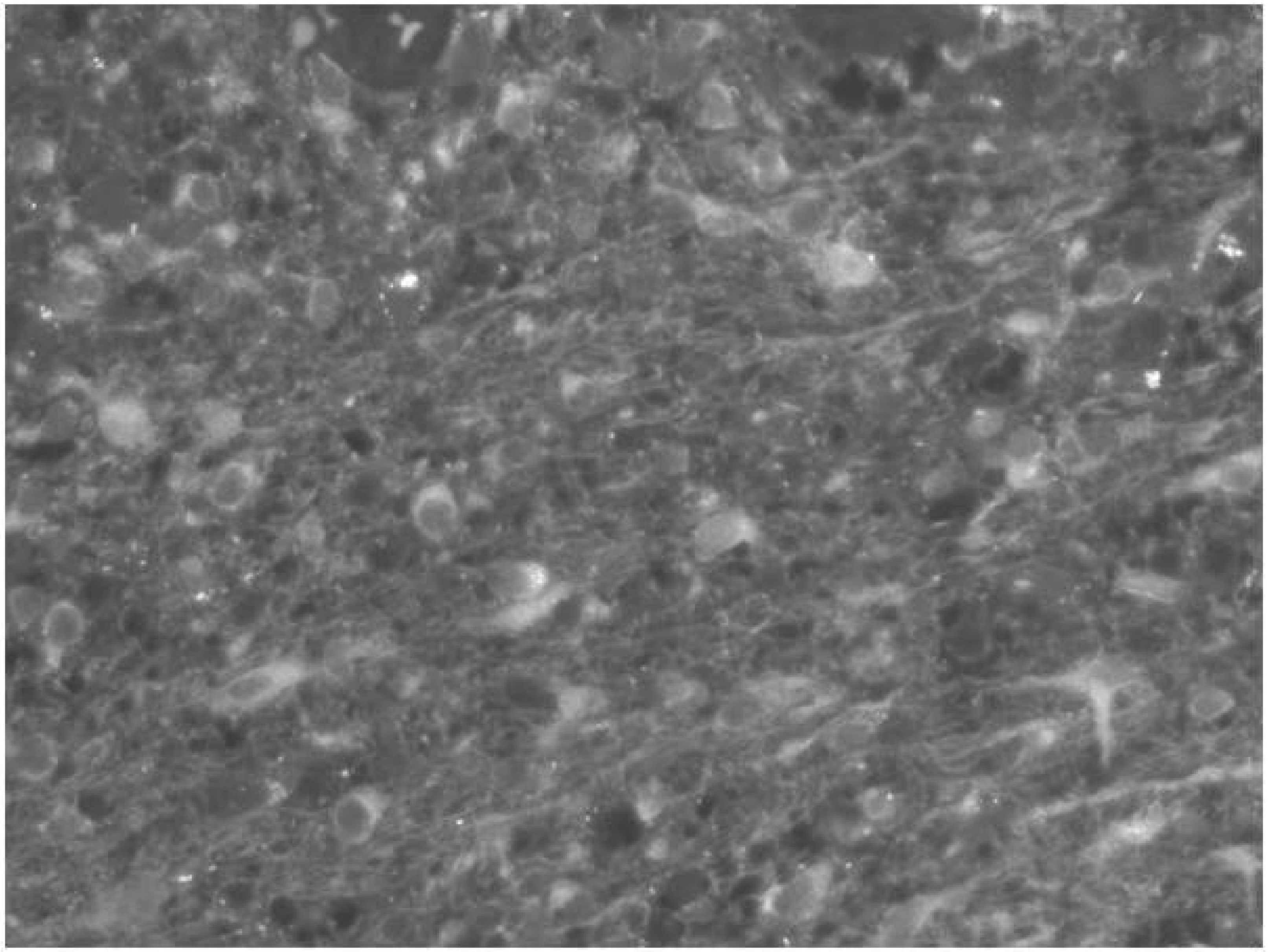

IDH1 R132H

Case4_ROI_7 IDH1 scoring

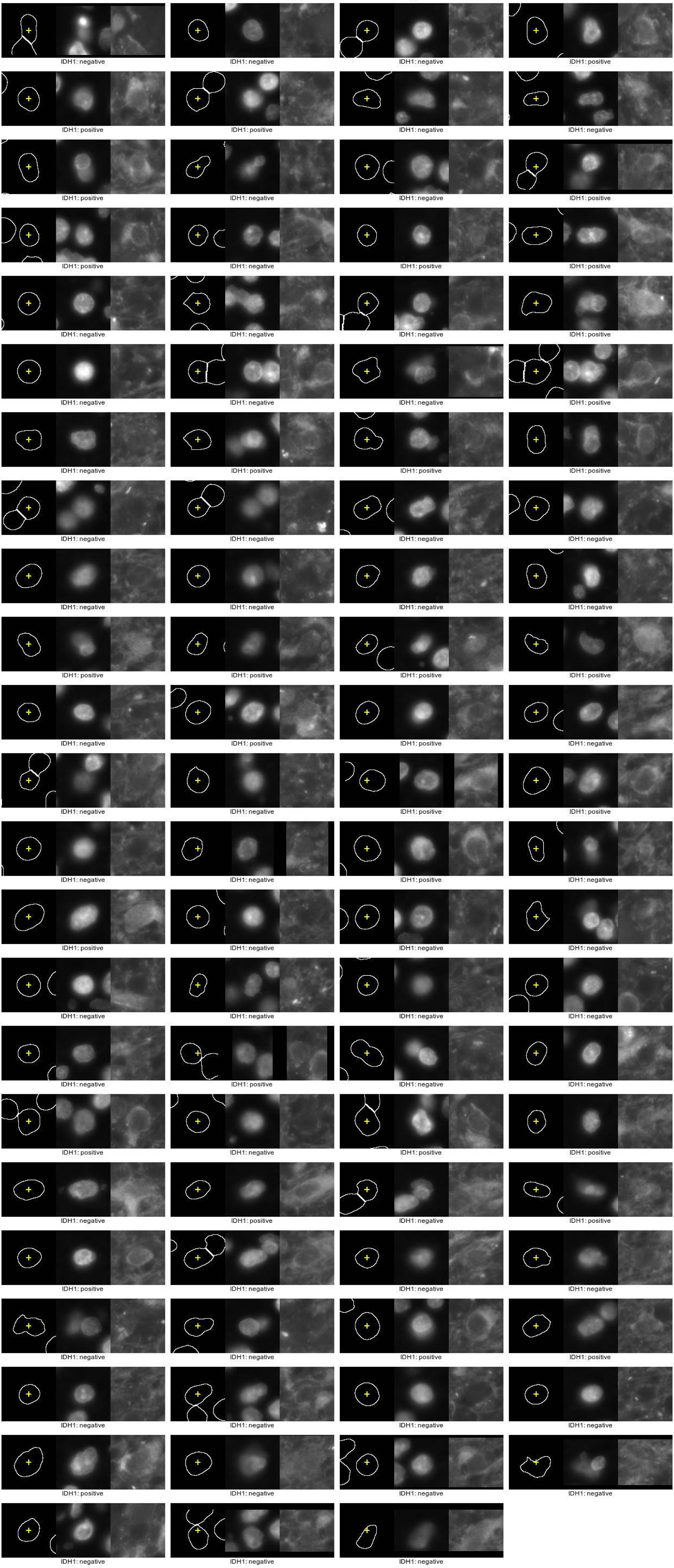

Case4_ROI_7 ZEB1 scoring

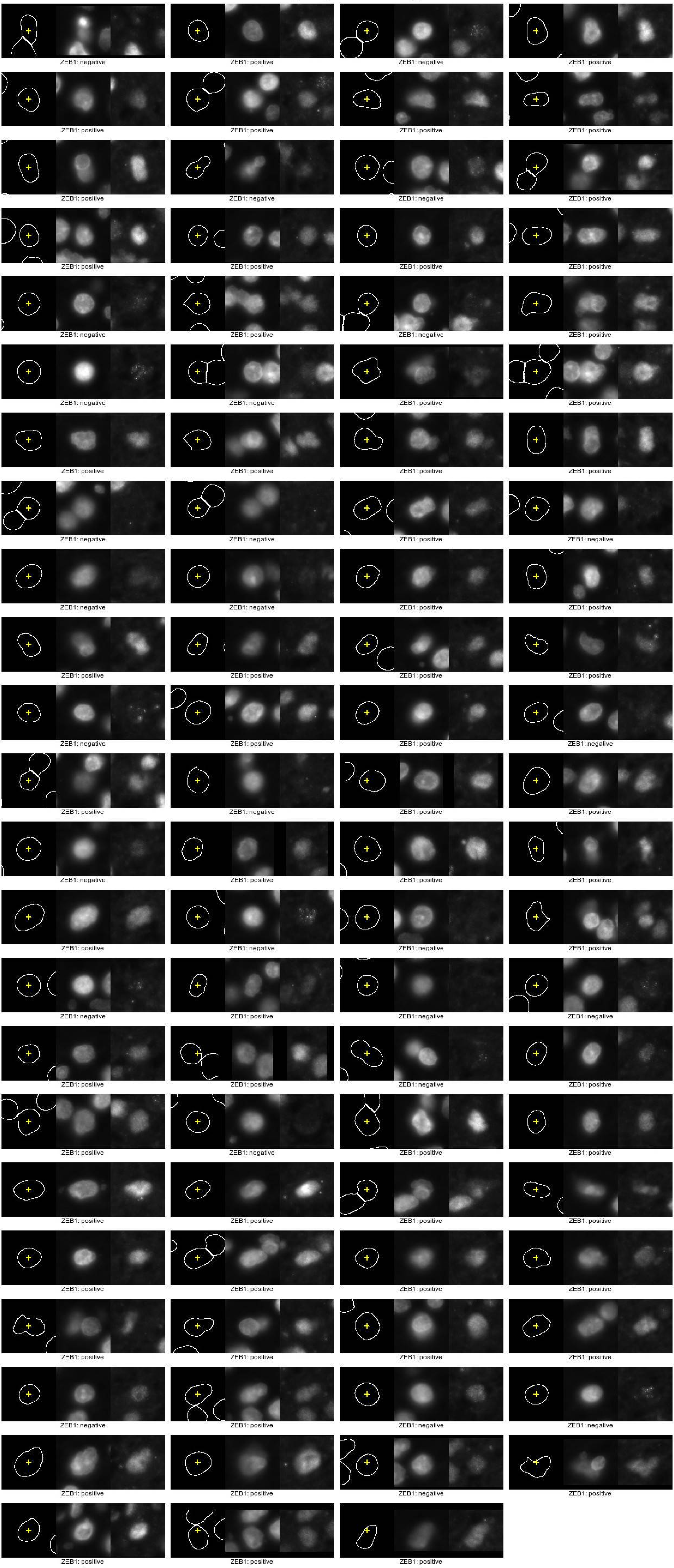

Case4_ROI_8 overview

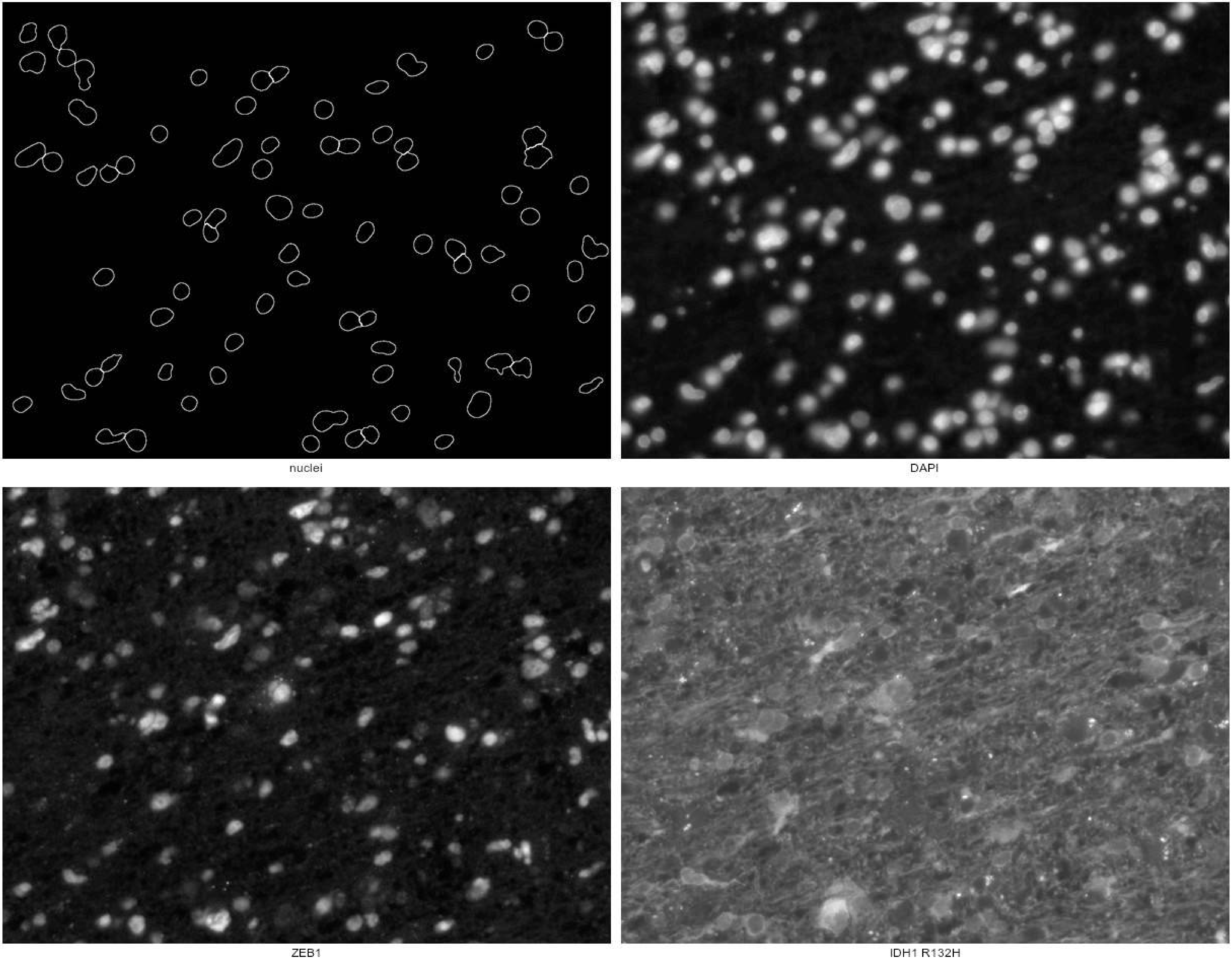

Case4_ROI_8 IDH1 scoring

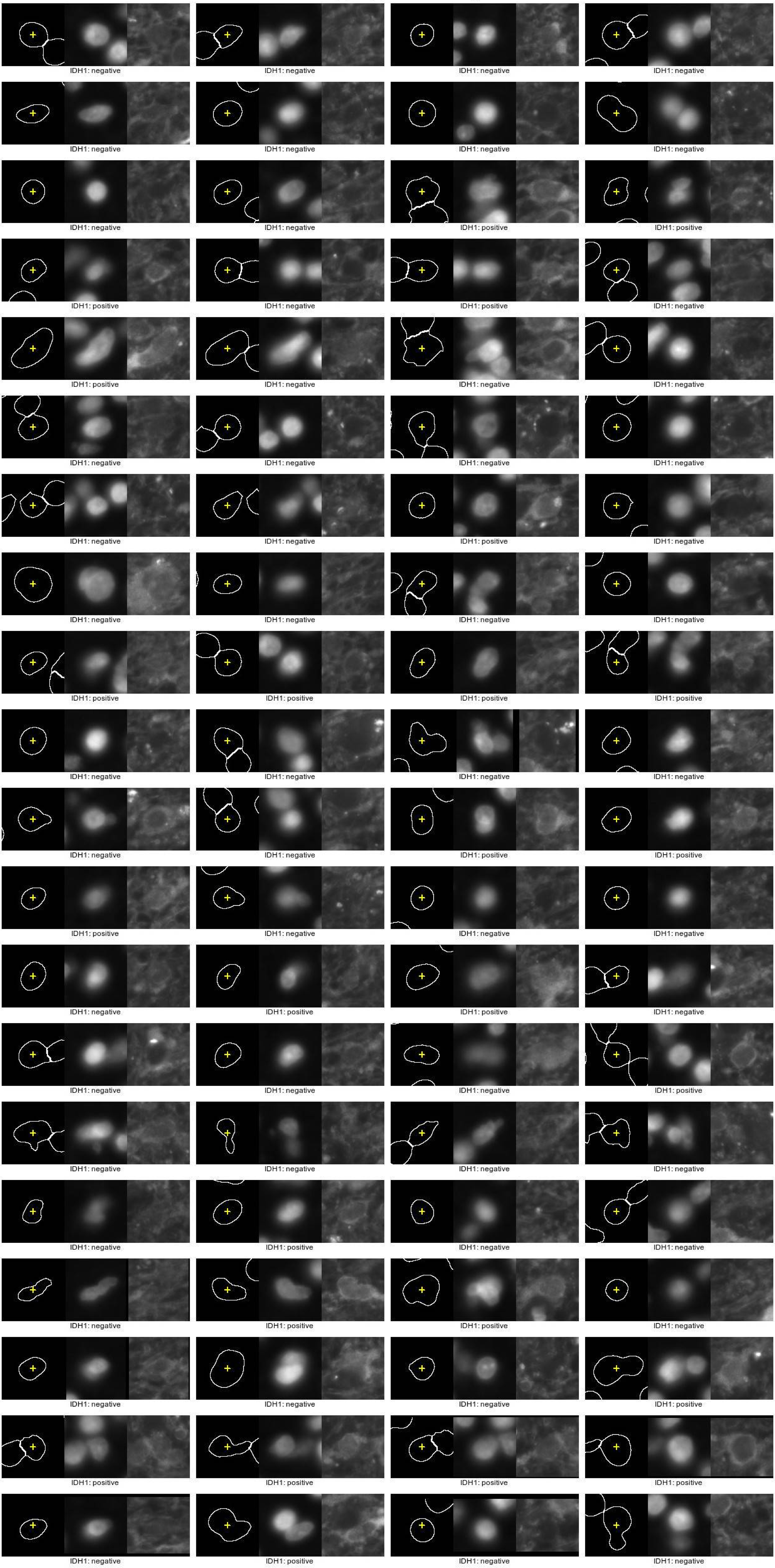

Case4_ROI_8 ZEB1 scoring

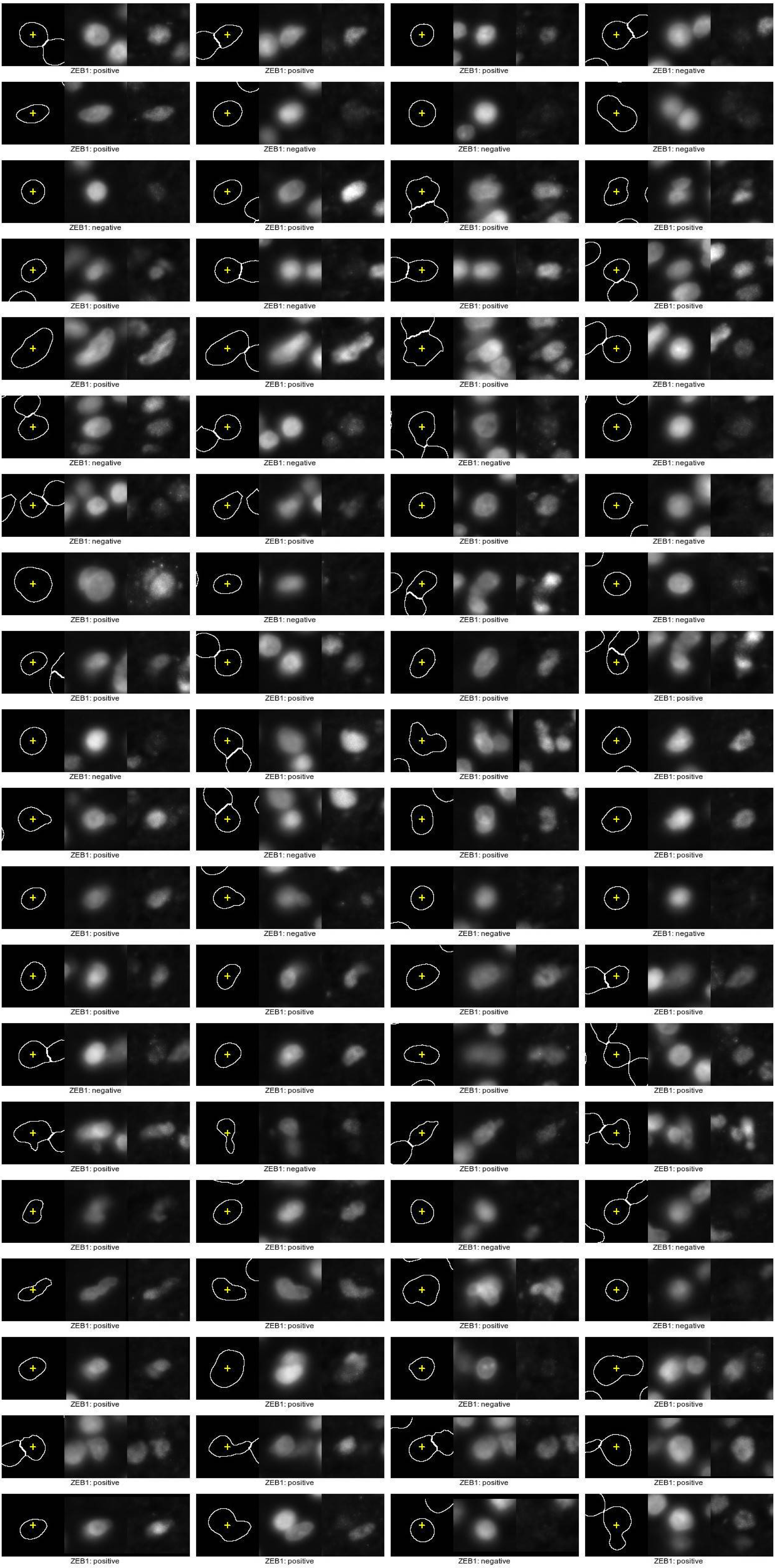

Case4_ROI_9 overview

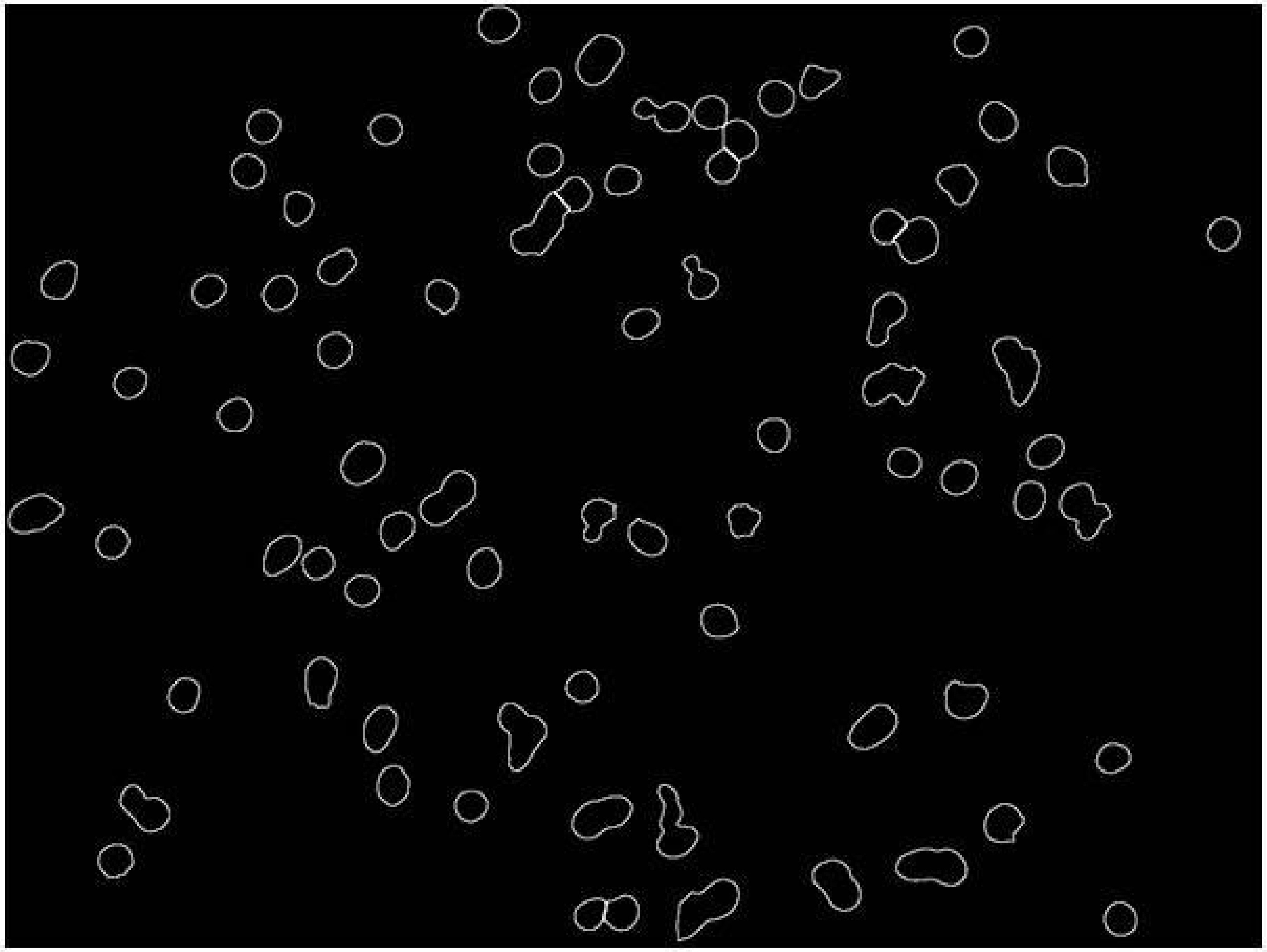

nuclei

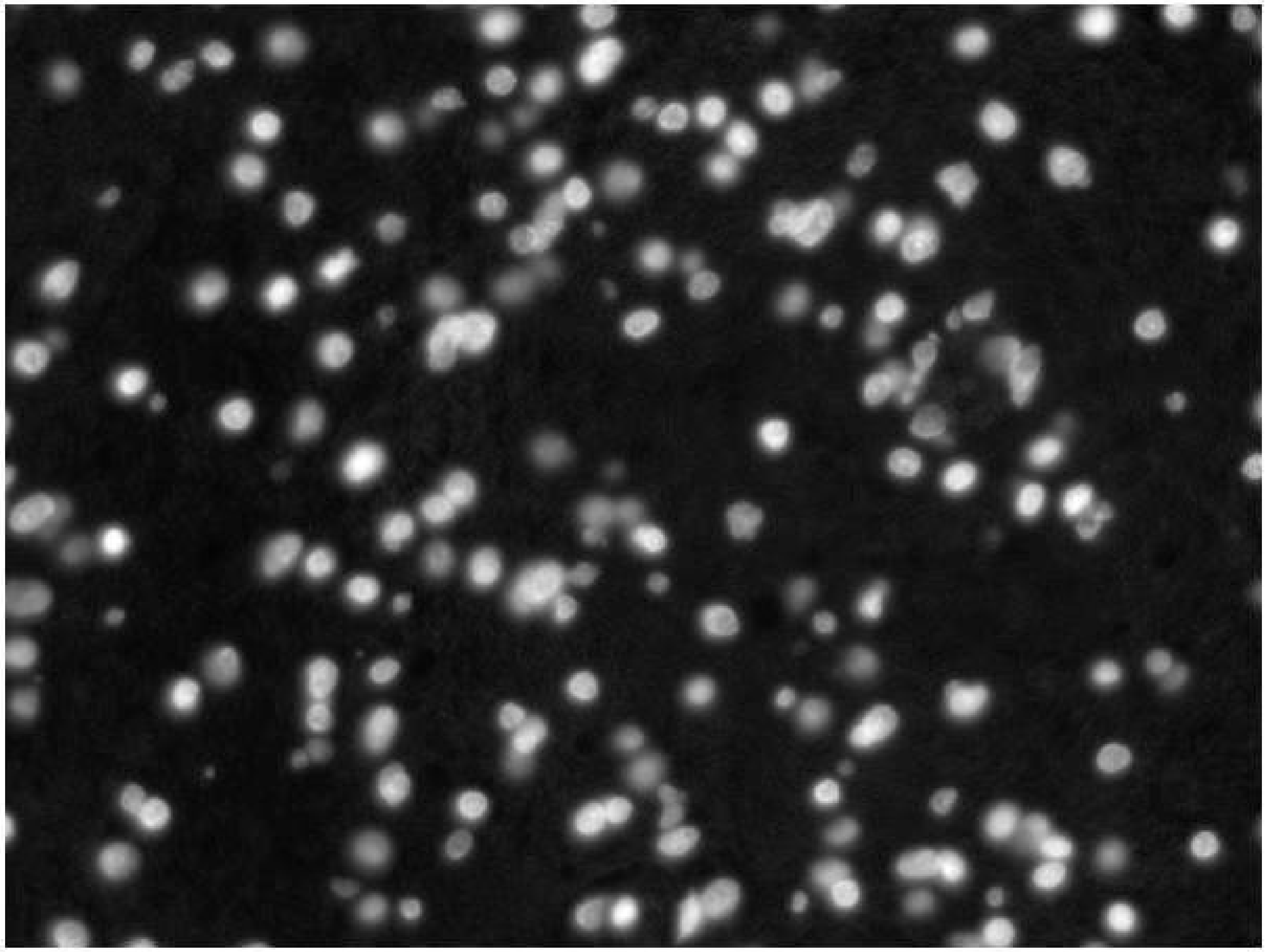

DAPI

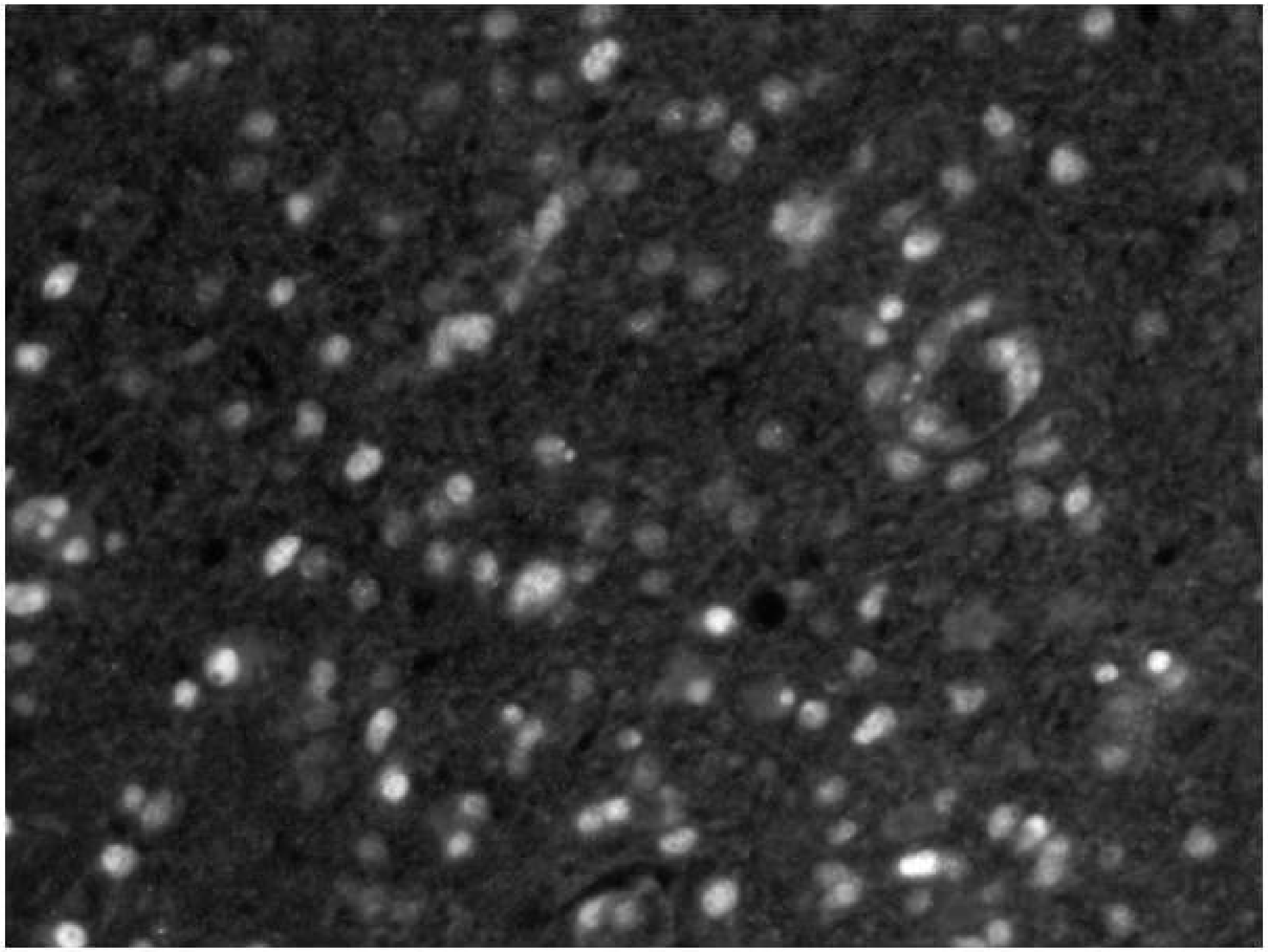

ZEB1

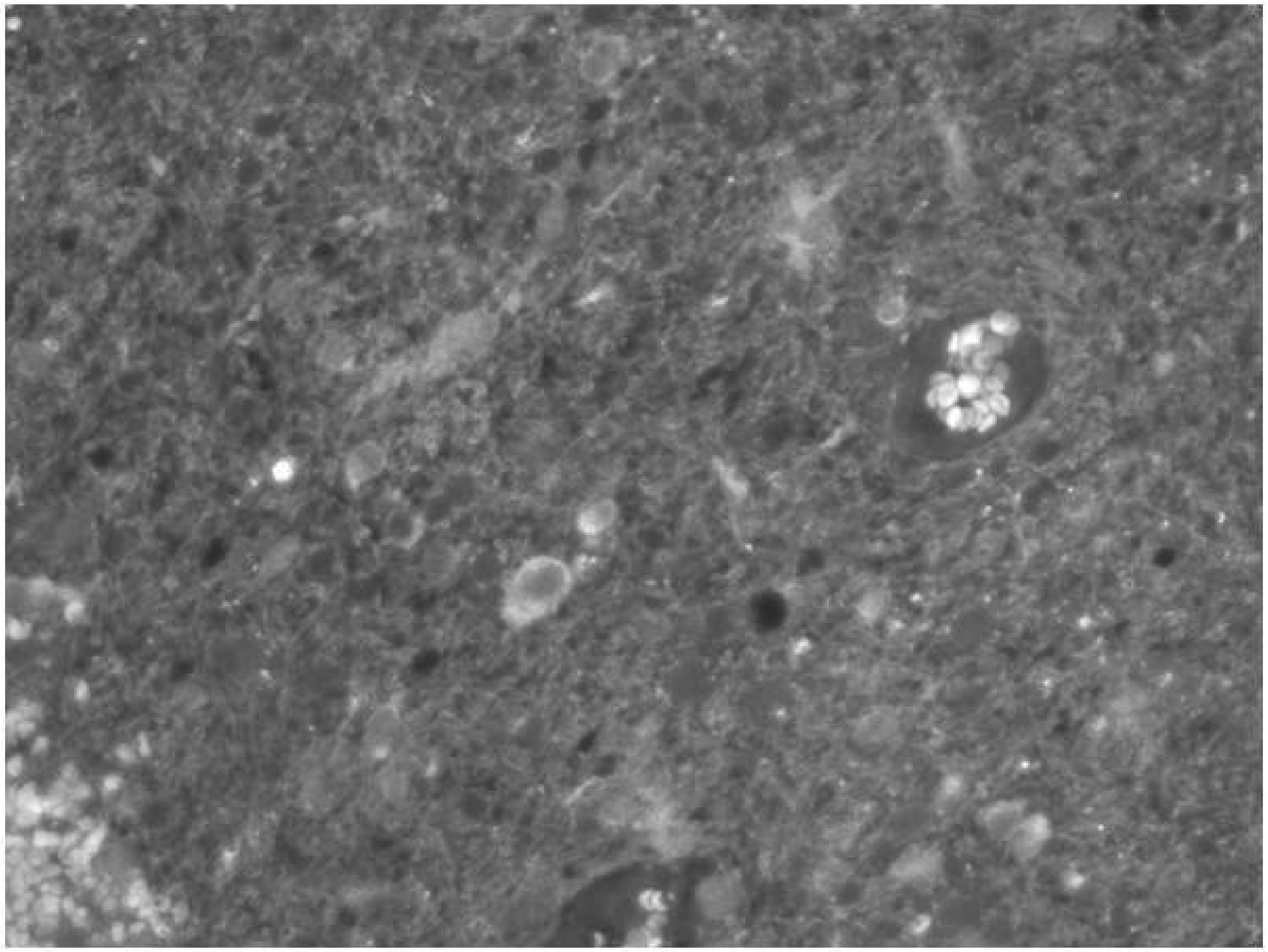

IDH1 R132H

Case4_ROI_9 IDH1 scoring

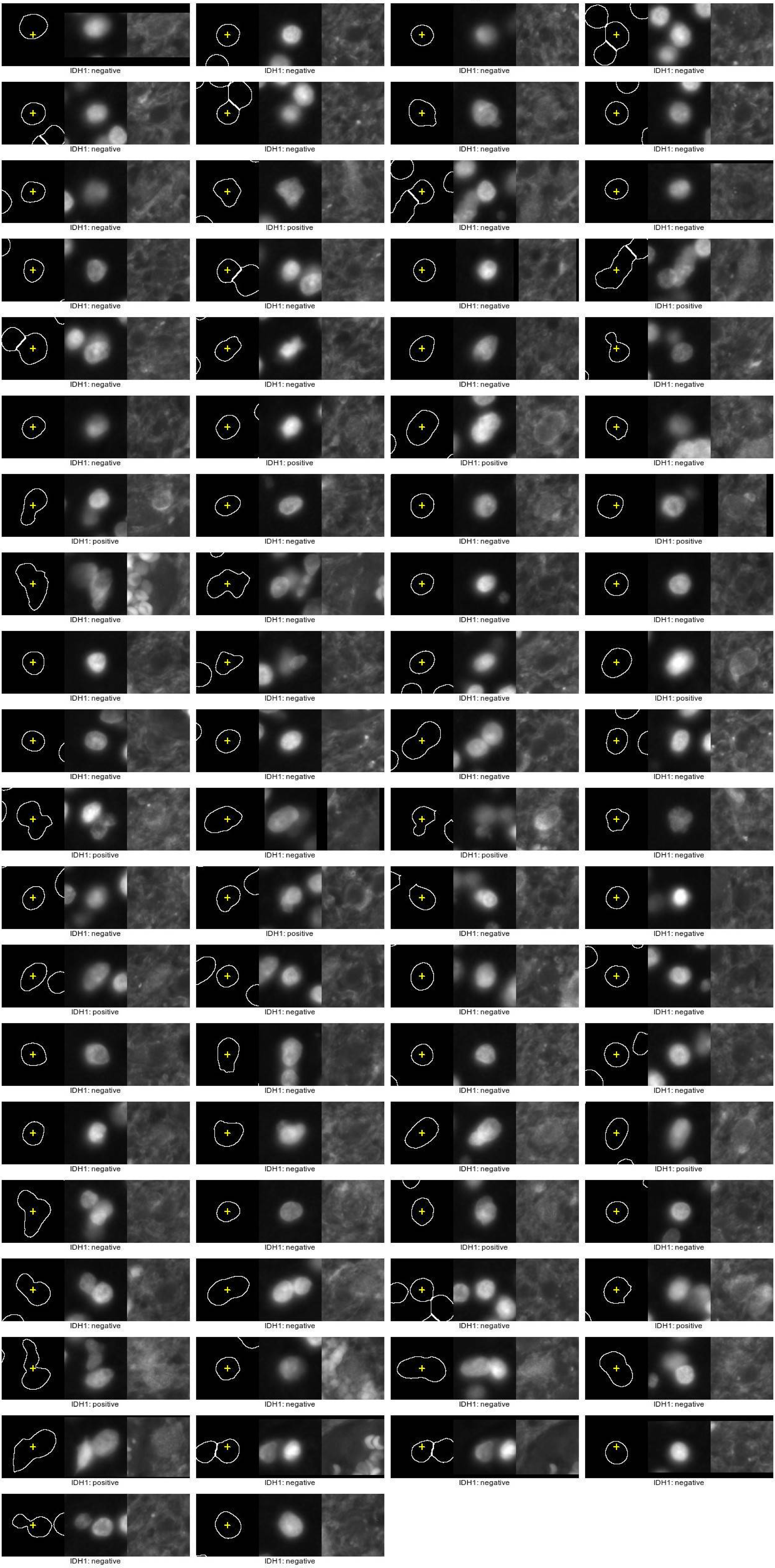

Case4_ROI_9 ZEB1 scoring

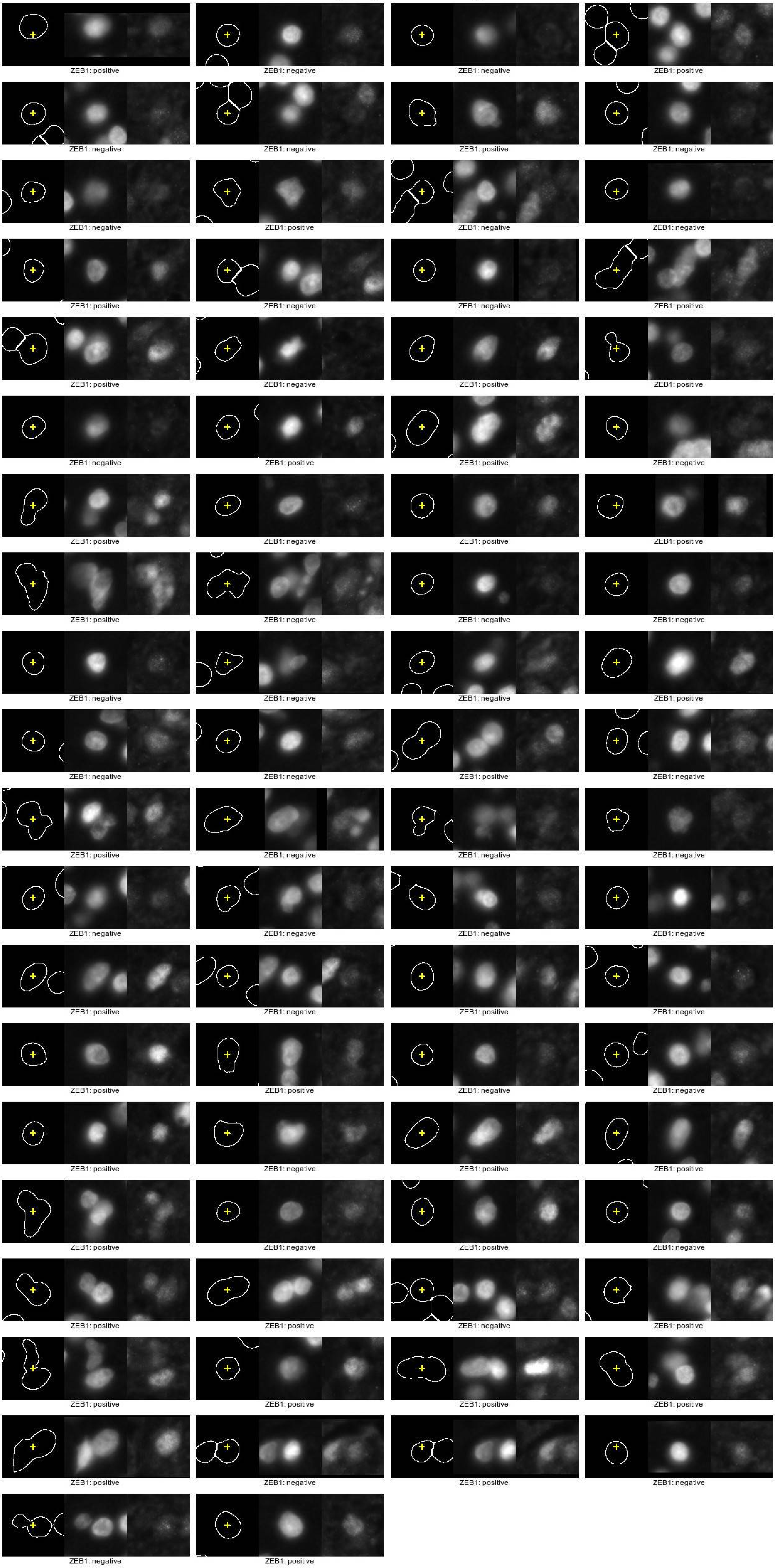

Supplement: S1 File — (PDF) [file pone.0185376.s004.pdf]
