# Supplementary material for: Cellular heterogeneity contributes to subtype-specific expression of ZEB1 in human glioblastoma
Source: PLoS One. 2017 Sep 25;12(9):e0185376. doi: 10.1371/journal.pone.0185376 (PMC5612763; doi:10.1371/journal.pone.0185376)
Supplement: S1 Table — (DOC) [file pone.0185376.s005.doc]

**Table S1. Overview of samples with quantifiable ZEB1 staining available.** Quantification of ZEB1 immunohistochemistry was possible for 245 out of a total of 266 cases.

| **diagnosis** | **no. of cases** | **ZEB1 labelling index (mean +/- SD)** |
| --- | --- | --- |
| ***full biopsy samples*** | | |
| astrocytoma, IDH-wildtype | 2 | 86 ± 12 |
| astrocytoma, IDH-mutant | 4 | 93 ± 4 |
| oligodendroglioma, IDH-mutant and 1p/19q-codeleted | 5 | 88 ± 12 |
| glioblastoma, IDH-wildtype | 11 | 72 ± 26 |
| glioblastoma, IDH-mutant | 1 | 94 |
| ***tissue microarray*** | | |
| anaplastic astrocytoma | 6 | 57 ± 30 |
| anaplastic oligodendroglioma | 1 | 54 |
| anaplastic oligoastrocytoma | 1 | 87 |
| glioblastoma (total)  IDH-wildtype  IDH-mutant | 210  197  13 | 51 ± 25  50 ± 25  65 ± 22 |
| gliosarcoma | 4 | 45 ± 34 |

Note: An integrated diagnosis according to WHO 2016 criteria was assigned to full biopsy cases. Histological grading was omitted for full biopsy samples due to the small size. In contrast, 1p/19q codeletion status was not available for the tissue microarray where the WHO 2007 histological diagnosis is given.
