# Supplementary material for: Cellular heterogeneity contributes to subtype-specific expression of ZEB1 in human glioblastoma
Source: PLoS One. 2017 Sep 25;12(9):e0185376. doi: 10.1371/journal.pone.0185376 (PMC5612763; doi:10.1371/journal.pone.0185376)
Supplement: S3 Table — (DOC) [file pone.0185376.s007.doc]

**Supplementary Table 3** *Multivariate analysis by mixed model linear regression of ZEB1 labelling index with respect to molecular and clinical properties (n = 166 cases).*

| **factor** | **regression coefficient** | **standard error** | **t value** | **p value** |
| --- | --- | --- | --- | --- |
| **EGFR expression** | **5.805e-01** | **1.247e-01** | **4.653** | **4.87e-06** |
| **IDH1 R132H mutation** | **2.643e-01** | **7.934e-02** | **3.331** | **0.00108** |
| p53 labelling index > 20% | 2.555e-02 | 2.088e-02 | 1.224 | 0.22197 |
| sex (female) | -3.153e-04 | 3.787e-02 | -0.008 | 0.99337 |
| age (years) | 1.512e-03 | 1.733e-03 | 0.873 | 0.38421 |
